# The world woodlouse flies (Diptera, Rhinophoridae)

**DOI:** 10.3897/zookeys.903.37775

**Published:** 2020-01-15

**Authors:** Pierfilippo Cerretti, Davide Badano, Silvia Gisondi, Giuseppe Lo Giudice, Thomas Pape

**Affiliations:** 1 Department of Biology and Biotechnologies ‘Charles Darwin’, Sapienza University of Rome, Piazzale A. Moro 5, I-00185, Rome, Italy; 2 DISTAV, University of Genoa, Corso Europa 26, I-16132, Genoa, Italy; 3 CUFA, Raggruppamento Carabinieri Biodiversità, Verona, Italy; 4 LaNaBIT – Laboratorio Nazionale Tassonomia e Biomonitoraggio Invertebrati, Verona, Italy; 5 Natural History Museum of Denmark, University of Copenhagen, Copenhagen, Denmark

**Keywords:** Catalogue, cladistic analysis, classification, distribution, new taxa, Oestroidea, parasitoids, systematics, zoological nomenclature

## Abstract

The world Rhinophoridae are catalogued, recognising 33 genera and 177 species. Nomenclatural information is provided for all genus-group and species-group names, including lists of synonyms and name-bearing type data. Species distributions are recorded by country. A key to the world genera is presented. Four new genera are erected to accommodate five new species, which do not fit within any of the current generic concepts in Rhinophoridae, according to the results of a morphology-based phylogenetic analysis: *Marshallicona* Cerretti & Pape with type species *Marshallicona quitu* Cerretti & Pape, **gen. et sp. nov.** (Ecuador); *Maurhinophora* Cerretti & Pape with type species *Maurhinophora indoceanica* Cerretti & Pape, **gen. et sp. nov.** (Mauritius); *Neotarsina* Cerretti & Pape with type species *Neotarsina caraibica* Cerretti & Pape, **gen. et sp. nov.** (Trinidad and Tobago) and *Neotarsina andina* Cerretti & Pape, **sp. nov.** (Peru); *Kinabalumyia* Cerretti & Pape with type species *Kinabalumyia pinax* Cerretti & Pape, **gen. et sp. nov.** (Malaysia, Sabah). The genus *Aporeomyia* Pape & Shima (type species *Aporeomyia antennalis* Pape & Shima), originally assigned to Tachinidae, is here reassigned to Rhinophoridae based on a reassessment of the homologies of the male terminalia. The following five species-group names, which were previously treated as junior synonyms or nomina dubia, are recognised as valid species names: *Acompomintho caucasica* (Villeneuve, 1908), **stat. rev.** [from nomen dubium to valid species]; *Acompomintho sinensis* (Villeneuve, 1936), **stat. rev.** [from nomen dubium to valid species]; *Stevenia bertei* (Rondani, 1865), **stat. rev.** [from nomen dubium to valid species]; *Stevenia sardoa* Villeneuve, 1920, **stat. rev.** [from junior synonym of *Rhinophora deceptoria* Loew, 1847 to valid species]; *Stevenia subalbida* (Villeneuve, 1911), **stat. rev.** [from junior synonym of *Rhinophora deceptoria* Loew, 1847 to valid species]. Reversal of precedence is invoked for the following case of subjective synonymy to promote stability in nomenclature: *Rhinophora lepida* (Meigen, 1824), **nomen protectum**, and *Musca parcus* Harris, 1780: 144, **nomen oblitum**. New generic and specific synonymies are proposed for the following two names: *Mimodexia* Rohdendorf, 1935, junior synonym of *Tromodesia* Rondani, 1856, **syn. nov.** and *Ptilocheta tacchetti* Rondani, 1865, junior synonym of *Stevenia obscuripennis* (Loew, 1847), **syn. nov.** The following new combinations are proposed: *Acompomintho sinensis* (Villeneuve, 1936), **comb. nov.** [transferred from *Tricogena* Robineau-Desvoidy, 1830]; *Tromodesia guzari* (Rohdendorf, 1935), **comb. nov.** [transferred from *Mimodexia* Rohdendorf, 1935]; *Tromodesia intermedia* (Rohdendorf, 1935), **comb. nov.** [transferred from *Mimodexia* Rohdendorf, 1935]; *Tromodesia lindneriana* (Rohdendorf, 1961), **comb. nov.** [transferred from *Mimodexia* Rohdendorf, 1935]; *Tromodesia magnifica* (Rohdendorf, 1935), **comb. nov.** [transferred from *Mimodexia* Rohdendorf, 1935]; *Tromodesia obscurior* (Rohdendorf, 1935), **comb. nov.** [transferred from *Mimodexia* Rohdendorf, 1935]; *Tromodesia pallidissima* (Rohdendorf, 1935), **comb. nov.** [transferred from *Mimodexia* Rohdendorf, 1935]; *Tromodesia setiventris* (Rohdendorf, 1935), **comb. nov.** [transferred from *Mimodexia* Rohdendorf, 1935] and *Tromodesia shachrudi* (Rohdendorf, 1935), **comb. nov.** [transferred from *Mimodexia* Rohdendorf, 1935].

## Introduction

Rhinophoridae are a small oestroid family with 33 genera and 177 species, recognised as of the present catalogue. The family was earlier considered by several authors to be entirely of Old World distribution and including very few native species outside the Palaearctic Region. This notion was at least partly due to the then superficially sampled tropical and southern hemisphere subtropical faunas, which are continuously revealing new taxa (Crosskey 1977, Colless 1994, Pape 1998a, Pape and Arnaud 2001, Cerretti and Pape 2012, Cerretti et al. 2014a).

**Figure 1.**
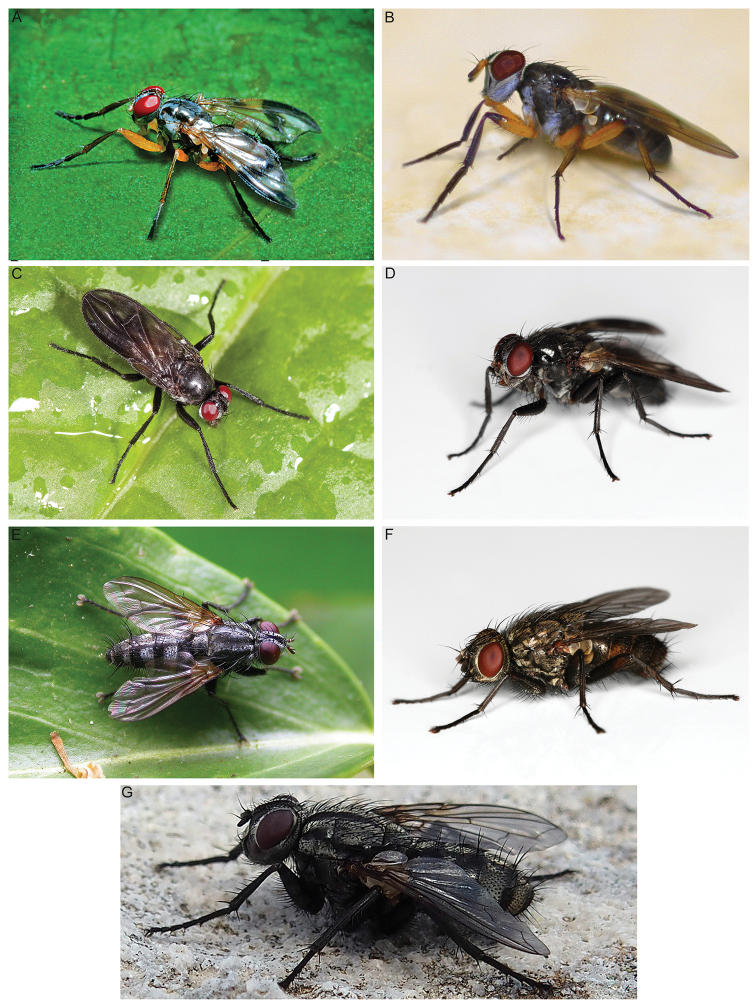
Rhinophoridae photographed in nature. **A***Bezzimyia* sp. (Ecuador) **B***Bixinia winkleri* (Australia) **C***Marshallicona quitu* (Ecuador) **D***Paykullia maculata* (Finland) **E***Stevenia* sp. (Italy) **F***Tricogena rubricosa* (Finland) **G***Phyto* sp. (Italy). Photographs by Steve Marshall (**A, C**), P.C. (**B, E, G**), Håkon Haraldseide (**D, F**).

Adult Rhinophoridae present no unique autapomorphies (Fig. 1), thus they are difficult to key out in conventional family-level keys (e.g., Crosskey 1977, Marshall 2012). However, their larvae are highly specialised woodlouse parasitoids and, to the extent they are known, provide unambiguous evidence for family affiliation and monophyly (Bedding 1973, Pape and Arnaud 2001, Cerretti et al. 2014a). The woodlouse-parasitising habit is unique not only within Diptera but within all Insecta. The distribution of Rhinophoridae largely matches that of woodlice, and the peak of rhinophorid diversity and abundance appears to be the Turano-Mediterranean area of the western Palaearctic (see below).

The family Rhinophoridae is a member of the Oestroidea, but its phylogenetic position within this clade is still unresolved (Pape 1992, Rognes 1997, Pape and Arnaud 2001, Kutty et al. 2010, Winkler et al. 2015, Cerretti et al. 2017, Cerretti et al. 2019, Stireman et al. 2019, Kutty et al. 2019). The most recent analyses based on morphology indicate a sister-group relationship to Rhiniidae (Pape and Arnaud 2001) or to the monotypic New Zealand family Mystacinobiidae, with Rhiniidae subordinate to these (Cerretti et al. 2017), which find most arguments from first instar larval morphology, which is very superficially known for the latter group (Ferrar 1987, Rognes 2002). Interestingly, the most recent analyses based on molecular data retrieve Rhiniidae as sister to Bengaliinae (Cerretti et al. 2017, Stireman et al. 2019, Kutty et al. 2019, Cerretti et al. 2019), and the extensive transcriptome data of Kutty et al. (2019) point to a phylogenetic grouping of Rhinophoridae with the macrolarviparous Helicoboscinae and Ameniinae, which is unexpected from a morphological as well as a biological point of view. The phylogenetic position of Rhinophoridae within Oestroidea remains ambiguous.

Peris and González-Mora (2007) compiled the first world catalogue for the family Rhinophoridae, but with insufficient distributional data and without data on type localities and types. Taking into account the substantial number of genera and species recently described from outside the Palaearctic, which have not been included in any regional catalogue, the significant recent exclusions (i.e., *Alvamaja chlorometallica* Rognes and the five species of the *Phyto carinata* species group now assigned to the genus *Morinia* Robineau-Desvoidy, see Cerretti et al. 2019) as well as a new inclusion (*Aporeomyia* Shima and Pape, as proposed herein) of taxa, we consider it both helpful and timely to provide a fully updated world catalogue of the entire family. We also take this opportunity to describe four new genera to accommodate five new species, produce a key to all genera, and perform a genus-level phylogenetic analysis of the Rhinophoridae in order to support the newly proposed genera in a cladistic framework.

## Materials and methods

### Systematics

Taxa, specimens

**Figure 2.**
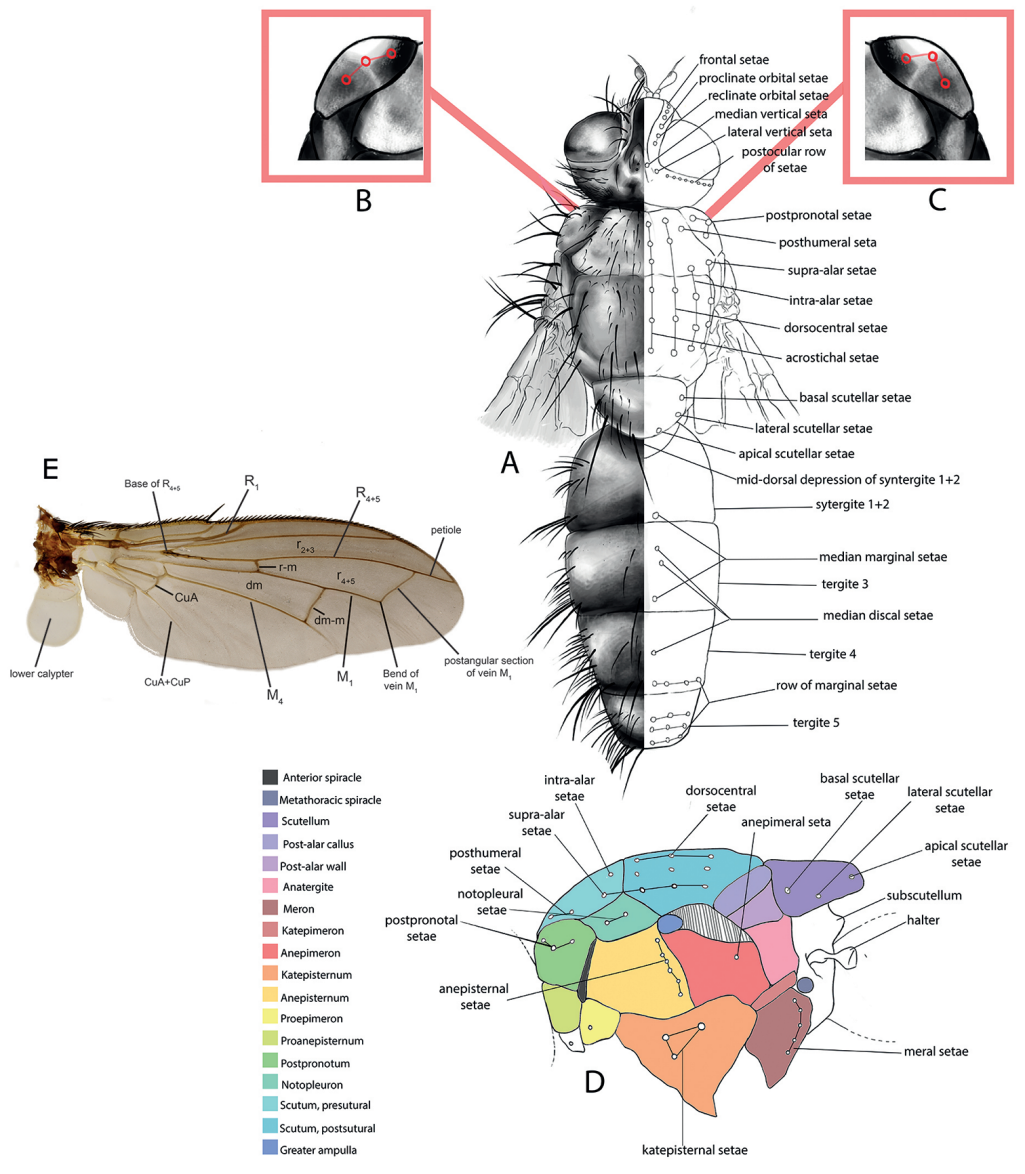
Morphological terminology of Rhinophoridae, head, thorax and abdomen. **A** Chaetotaxy of head, thorax and abdomen in dorsal view **B, C** arrangement of setae of postpronotum: **B** three setae arranged in shallow triangle **C** three setae standing in a nearly right-angled triangle **D** thorax, sclerites and chaetotaxy, in lateral view **E** wing veins and cells. Drawings by Giulia Bellanti.

**Figure 3.**
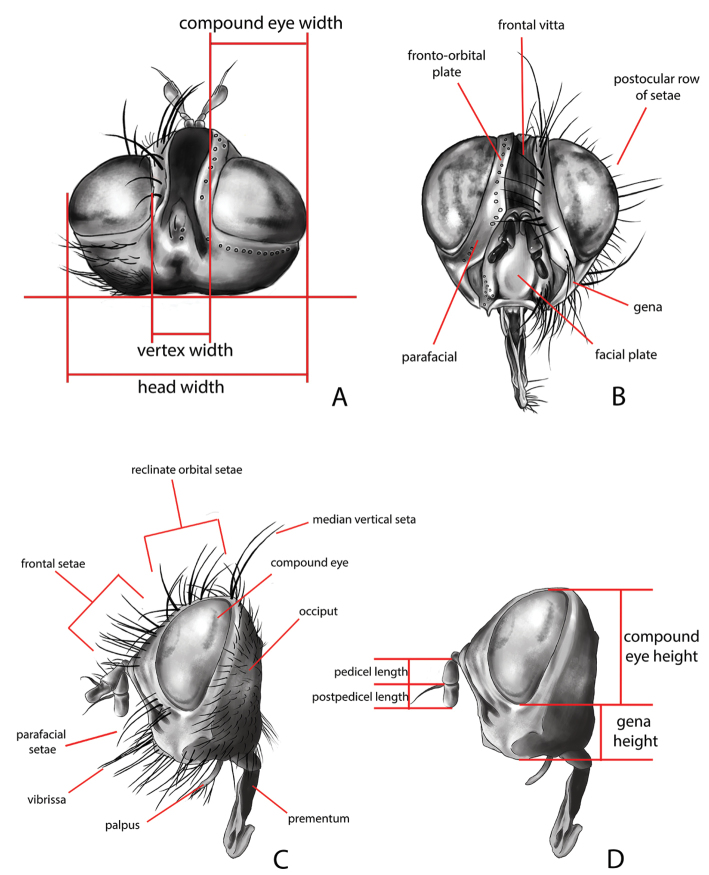
Morphological terminology of Rhinophoridae, head. **A** Standard measurements in dorsal view **B** head structures in frontal view **C** head structures in lateral view **D** standard measurements in lateral view. Drawings by Giulia Bellanti.

All genera were studied, based on an extensive representation of the included species, in order to construct a key to the genera of the world and perform a genus-level phylogenetic analysis. Dissections of male terminalia were performed according to the procedure described by Cerretti and Shima (2011). Briefly, this procedure involves the removal of the abdomen, partial clearing in 10% KOH, dissection and rinsing of terminalia, reattachment of the abdomen to the specimen, and final storage of the terminalia in a glycerine-filled microvial pinned with the source specimen. Figures 2 and 3 summarise the morphological terminology of the adults (except terminalia) and the measurements applied in the present work. Morphological terminology of terminalia (both male and female) and preimaginal instars follow Cumming and Wood (2017) and Pape and Arnaud (2001), respectively.

Data from each type specimen are given verbatim, with information for each line separated by a slash (/) ; in cases with more than one label, these are separated with a double slash (//). Additional information of relevance, but not appearing on the label(s), is given in brackets. Finally, the acronym of the repository is cited in parentheses.

Photography, SEM

Photographic images of habitus, head, wing, legs, abdomen and male terminalia were produced using a MZ 12.5 stereoscopic microscope (Leica, Germany) equipped with a DS-L1 Nikon digital camera (Nikon, Tokyo). Photographic images of terminalia were produced using a DM LS microscope (Leica, Germany) equipped with the camera described above. Focus stacking with the image stacking software CombineZM (Hadley, UK) was used to merge 15–45 photos of each specimen/structure, taken at different focal planes, into high-resolution images. Additional images were produced with a Hitachi TM1000 environmental scanning electron microscope (ESEM) from uncoated pinned specimens, using Adobe Photoshop for colouration of specific structural details.

Phylogenetic analysis

We adapted the data matrix of Cerretti et al. (2014a) to assess the most probable phylogenetic affinities and associated support of the newly proposed genera. The matrix consists of 99 morphological characters: 82 from adult stage (1–52: body of both sexes, except terminalia; 53–81: male terminalia; 82: female terminalia), 17 from first instar larva (Table 1); rhinophorid diversity is represented by a sample of 57 species (comprising at least one species for each genus), plus *Musca* spp. (Muscidae) and a selection of oestroid taxa as outgroups (Table 2; the nexus format of the data matrix is available from MorphoBank Project 3576). In the case of *Kinabalumyia pinax* gen. et sp. nov., described in the present paper based on two males from Sabah (Malaysia), female characters were scored from a female from Palawan (Philippines), which is here considered congeneric but is left unidentified (*Kinabalumyia* sp. 1, see below); the two sets of characters were concatenated and treated as a single operational taxonomic unit (OTU) in all our analyses, which is shown in the tree under the name of *Kinabalumyia pinax* for practical reasons.

**Table 1.** List of characters and states.

**ADULT (body parts, except terminalia)**	
1	**Arista, microtrichia length**: **(0)** shorter than maximum diameter of arista; **(1)** distinctly longer.
2	**Male arista, microtrichia**: **(0)** not bottlebrush-like; **(1)** bottlebrush-like.
3	**Arista, development**: **(0)** normally developed; **(1)** shortened.
4	**Arista, setae**: **(0)** absent; **(1)** present.
5	**Arista, thickening**: **(0)** at most on basal fourth; **(1)** at least on basal 3/4.
6	**First aristomere, length**: **(0)** short, at most as long as wide; **(1)** approx. 2–3 times as long as wide; **(2)** at least 4 times as long as wide.
7	**Lunule, setae**: **(0)** bare; **(1)** with setae.
8	**Female, groove between fronto-orbital plate and parafacial**: **(0)** absent; **(1)** present.
9	**Male, proclinate orbital setae**: **(0)** absent; **(1)** present, at least as long as frontal setae; **(2)** present, distinctly shorter than frontal setae.
10	**Female, proclinate orbital setae**: **(0)** absent; **(1)** present, at least as long as frontal setae; **(2)** present, distinctly shorter than frontal setae.
11	**Proclinate orbital setae, when present, number**: **(0)** one or two; **(1)** more than two.
12	**Vibrissal triangle**: **(0)** normal; **(1)** projected.
13	**Shape of lower facial margin, shape**: **(0)** not sunken; **(1)** deeply sunken (i.e., concave).
14	**Facial plate, shape**: **(0)** not sunken; **(1)** deeply sunken.
15	**Facial plate, median carina**: **(0)** absent; **(1)** present.
16	**Compound eye, posterior margin**: **(0)** not indented; **(1)** indented.
17	**Parafacial, setosity**: **(0)** bare in ventral half; **(1)** setose in ventral half.
18	**Parafacial setae, configuration**: **(0)** short, scattered and proclinate; **(1)** long, robust and medioclinate.
19	**Frontal setae, orientation of dorsal-most pair**: **(0)** converging in the middle and crossed; **(1)** sub-parallel.
20	**Mouthparts, development**: **(0)** normally developed (i.e., prementum between 2 and 6 times as long as wide); **(1)** both prementum and labella strongly reduced (vestigial).
21	**Palpus**: **(0)** normally developed; **(1)** reduced; **(2)** absent.
22	**Occiput, setae**: **(0)** black, normal; **(1)** pale and hair-like.
23	**Postpronotal setae, number**: **(0)** four (or more) setae; **(1)** three setae; **(2)** two setae; **(3)** one seta; **(4)** none.
24	**Postpronotal setae, if three or more, position**: **(0)** mid-basal seta in line, or nearly so, with inner and outer basal setae; **(1)** mid-basal seta displaced anteriorly to line between inner and outer basal setae (i.e., three robust, basal setae in an almost right-angled triangle).
25	**First postsutural supra-alar seta, size**: **(0)** present, approx. as long as notopleural setae; **(1)** absent or very short and hair-like; **(2)** strong, distinctly longer and thicker than notopleural setae.
26	**Subscutellum, development**: **(0)** flat or concave; **(1)** moderately swollen; **(2)** strongly swollen.
27	**Subscutellum, sclerotisation**: **(0)** not fully sclerotised; **(1)** entirely sclerotised.
28	**Scutellum, apical setae**: **(0)** present; **(1)** absent.
29	**Katepimeron, setae**: **(0)** with at least one seta anteriorly; **(1)** entirely bare.
30	**Metathoracic spiracular lappets**: **(0)** practically absent; **(1)** small, sub-equal in size and directed outwards; **(2)** unequal in size (posterior one distinctly larger) and both lappets closing the spiracle like an operculum.
31	**Male fore tarsus**: **(0)** normal; **(1)** laterally compressed.
32	**Female fore tarsus**: **(0)** normal; **(1)** laterally compressed.
33	**Shape of tibiae of mid and hind legs**: **(0)** normal; **(1)** laterally compressed and distinctly keeled dorsally.
34	**Leg chaetotaxy**: **(0)** not particularly modified; **(1)** reduced and with almost no (strong) setae.
35	**Hind coxa, posterodorsal margin**: **(0)** bare; **(1)** with (1–4) setae.
36	**Lower calypter, shape**: **(0)** semi-circular; **(1)** tongue-shaped.
37	**Long trichia along margin of lower calypter**: **(0)** absent; **(1)** present.
38	**Female wing, development**: **(0)** normally developed; **(1)** brachypterous; **(2)** micropterous.
39	**Costa, indentation at level of R_4+5_**: **(0)** absent; **(1)** present.
40	**Male wing pattern, three distinct whitish spots**: **(0)** absent; **(1)** present.
41	**Female wing pattern, posterodistal whitish spot**: **(0)** absent; **(1)** present.
42	**Vein R_1_, dorsal setae**: **(0)** absent; **(1)** with a row of setulae along whole length; **(2)** with 1–10 setae at most on apical fourth.
43	**Vein R_4+5_, dorsal setae**: **(0)** present; **(1)** absent.
44	**Vein R_4+5_, extent of dorsal setae, when present**: **(0)** only at base; **(1)** reaching at least crossvein r-m;
45	**Bend of vein M**_1_: **(0)** distinct; **(1)** not distinct.
46	**Vein M**_1_, **position of bend**: **(0)** well removed from wing margin; **(1)** very close to wing margin but distinct.
47	**Vein M**_1_, **extent of distal part**: **(0)** not vanishing on wing margin; **(1)** gradually vanishing on wing membrane.
48	**Vein M**_1_, **apical termination**: **(0)** joining R_4+5_ so that cell r_4+5_ is closed; **(1)** joining costa, so that cell r_4+5_ is open.
49	**Crossvein dm-m, inclination**: **(0)** forming a right angle with proximal section of M_4_; **(1)** forming an acute angle with proximal section of M_4_.
50	**CuA+CuP**: **(0)** not reaching wing margin; **(1)** reaching wing margin.
51	**Tergite 3, median marginal setae**: **(0)** present; **(1)** absent or very short and recumbent.
52	**Tergite 4, marginal setae**: **(0)** a regular row of more or less erect marginal setae; **(1)** marginal setae absent or not differentiated from general abdominal setulae.
**ADULT (male terminalia)**	
53	**Sternite 5, posteromedian notch**: **(0)** present; **(1)** absent.
54	**Sternite 5, shape of transversal section**: **(0)** U-shaped (i.e., folds up laterally); **(1)** almost flat.
55	**Sternite 5, median tooth-like apophysis on lateral lobe**: **(0)** absent; **(1)** present.
56	**Tergite 6, median marginal setae**: **(0)** present; **(1)** absent.
57	**Tergite 6, shape**: **(0)** normal (plate-like); **(1)** posteriorly indented; **(2)** divided into two hemitergites; **(3)** reduced, absent.
58	**Connection between tergite 6 and syntergosternite 7+8**: **(0)** membranous; **(1)** tergite 6 and syntergosternite 7+8 fused.
59	**Connection between sternite 6 and syntergosternite 7+8 on right side**: **(0)** membranous; **(1)** fused.
60	**Cerci, shape**: **(0)** normally developed; **(1)** short and sub-globular.
61	**Cerci, medial connection**: **(0)** not fused at all medially; **(1)** at least partly fused medially.
62	**Surstylus, shape**: **(0)** not divided; **(1)** with a large base distally divided into a lateral, wide, rounded lobe and into a postero-median finger-like apophysis.
63	**Surstylus, bifid inner median extension**: **(0)** absent; **(1)** present.
64	**Surstylus, setae on median extension**: **(0)** absent; **(1)** present.
65	**Bacilliform sclerite and laterobasal margin of surstylus, connection**: **(0)** articulated, not fused; **(1)** firmly fused.
66	**Surstylus and epandrium, connection**: **(0)** membranous; **(1)** surstylus and epandrium fused.
67	**Hypandrial arms, shape**: **(0)** firmly fused postero-medially, entirely encircling base of phallus; **(1)** more or less converging medially but not fused.
68	**Phallic guide and pregonites, connection**: **(0)** membranous (i.e., not fused); **(1)** sclerotised (i.e., fused).
69	**Postgonite, anterior seta**: **(0)** present; **(1)** absent.
70	**Extension(s) of dorsal sclerite of distiphallus**: **(0)** present; **(1)** absent.
71	**Extension(s) of dorsal sclerite of distiphallus, longitudinal division**: **(0)** divided into two hemisclerites or at least partly unfused medially (i.e., distally bifid); **(1)** entirely fused mid-dorsally into a single sclerotisation (i.e., distally not bifid).
72	**Extension(s) of dorsal sclerite of distiphallus, sclerotised connection with dorsal sclerite of distiphallus**: **(0)** present; **(1)** absent.
73	**Membranous flag distal to extension of dorsal sclerite of distiphallus**: **(0)** absent; **(1)** present.
74	**Median process of ventral sclerotisation of distiphallus**: **(0)** present; **(1)** absent.
75	**Median process of ventral sclerotisation of distiphallus, connection with ventral plate**: **(0)** not interrupted, running from the ventral plate to tip of phallus; **(1)** interrupted proximally and not connected to ventral plate.
76	**Median process of ventral sclerotisation of distiphallus, longitudinal division**: **(0)** not divided longitudinally; **(1)** divided longitudinally.
77	**Median process of ventral sclerotisation of distiphallus, length**: **(0)** normal; **(1)** very long (i.e., ending far beyond tip of acrophallus).
78	**Median process of ventral sclerite of distiphallus, asymmetry**: **(0)** absent; **(1)** present.
79	**Acrophallus, shape**: **(0)** simple, unmodified (with one opening); **(1)** distinctly tripartite (with three openings).
80	**Semi-cylindrical dorsal sclerite of acrophallus**: **(0)** absent; **(1)** present.
81	**Distiphallus, helmet-shaped, partly sclerotised envelope**: **(0)** absent; **(1)** present.
**ADULT (female terminalia)**	
82	**Ovipositor**: **(0)** long and telescopic; **(1)** shortened.
**LARVA (first instar)**	
83	**Labrum**: **(0)** well developed; **(1)** reduced.
84	**Locomotory behaviour**: **(0)** creeping (peristalsis); **(1)** leech-like; **(2)** somersaulting.
85	**Antenna**: **(0)** normally developed; **(1)** long and tapering.
86	**Posterior part of anal division modified as a terminal sucker**: **(0)** no; **(1)** yes.
87	**Mandibles**: **(0)** well developed; **(1)** reduced.
88	**Shape of mandible**: **(0)** normal, hook-like; **(1)** toothed or serrated.
89	**Labrum, connection with cephaloskeleton**: **(0)** not fused; **(1)** fused.
90	**Body shape**: **(0)** subfusiform; **(1)** slightly flattened dorsoventrally.
91	**Parastomal bar of cephaloskeleton**: **(0)** reduced or not elongated; **(1)** long and slender.
92	**Longitudinal incision on parastomal bar of cephaloskeleton**: **(0)** absent; **(1)** present.
93	**Fleshy protuberances (= prolegs) on segments**: **(0)** absent; **(1)** present.
94	**Shape of ventral part of pseudocephalon**: **(0)** normal (i.e., not elongated); **(1)** elongated.
95	**Longitudinal cuticular ridges posteroventrally on anal division**: **(0)** absent; **(1)** present.
96	**Tongue-like projection posterodorsally on anal division**: **(0)** absent; **(1)** present.
97	**Pair of more or less elongated or globular vesicles posteroventrally on anal division**: **(0)** absent; **(1)** present.
98	**Shape of posteroventral vesicles, is present**: **(0)** sub-globular; **(1)** long and tapering.
99	**Mandible, number of teeth (if present)**: **(0)** two; **(1)** three or more.

**Table 2.** List of material examined for the cladistic analysis. An asterisk (*) denotes taxa not included in the dataset of Cerretti et al. (2014a).

Family/subfamily	Genus/species	Country, State or Region [collection acronym]	References
Muscidae/Muscinae	*Musca* spp.	Italy, Latium [MZUR]	–
Calliphoridae/Ameniinae	*Amenia* sp.	Australia, Queensland [MZUR]	Crosskey (1965)
Calliphoridae/Bengaliinae	*Bengalia* sp.	Thailand, Chiang Mai [MZUR]	Rognes (2011)
Calliphoridae/Calliphorinae	*Calliphora vomitoria* (Linnaeus, 1758)	Italy [MZUR]	Rognes (1991) Rognes (1997)
Calliphoridae/Helicoboscinae	*Eurychaeta muscaria* (Meigen, 1826)	Italy, Latium and Veneto [MZUR]	Rognes (1986) Rognes 1991)
Calliphoridae/Luciliinae	*Lucilia sericata* (Meigen, 1826)	Italy, Sardinia [MZUR]	Rognes (1991)
Calliphoridae/Melanomyiinae	*Melinda gentilis* Robineau-Desvoidy, 1830	Italy, Sardinia [MZUR]	Rognes (1991)
Oestridae/Cuterebrinae	*Cuterebra austeni* Sabrosky, 1986	USA, New Mexico [MZUR]	–
Polleniidae	*Pollenia paupera* Rondani, 1862	Italy, Latium [MZUR]	–
Rhiniidae	*Rhyncomya impavida* (Rossi, 1790)	Italy, Latium [MZUR]	–
Tachinidae/Tachininae	*Macquartia tenebricosa* (Meigen, 1824)	Italy, Latium [MZUR]	–
Rhinophoridae	Acompomintho lobata*	Japan, Fukuoka [NHMD]	Pape (1986); Kato and Tachi (2016)
	*Apomorphyto inbio*	Costa Rica, Guanacaste [INBio]	–
	*Aporeomyia* sp.*	Malaysia, Sabah [CNC, NHMD]	Pape and Shima (1993)
	*Axinia disjuncta*	Australia, Queensland [ANIC]	Colless (1994)
	*Axinia lucaris*	Australia, Queensland [NHRS]	Colless (1994)
	*Axinia miranda*	Papua New Guinea [ANIC]	Colless (1994)
	*Azaisia* sp.*	Portugal, Madeira [NHMD]	–
	*Baniassa fascipennis*	Israel [TAU]	–
	*Bezzimyia barbarista*	Costa Rica, Alajuela [NHMD]	Pape and Arnaud (2001)
	*Bezzimyia bisecta*	Costa Rica: Monteverde and Guanacaste [NHMD]	Pape and Arnaud (2001) Pape 2010)
	*Bezzimyia busckii*	Costa Rica, Guanacaste [NHMD]	Pape and Arnaud (2001) Pape (2010)
	*Bezzimyia hansoni*	Costa Rica, Limón [NHMD]	Pape and Arnaud (2001) Pape (2010)
	*Bezzimyia yepezi*	Costa Rica, Puntarenas [NHMD]	Pape and Arnaud (2001)
	*Bixinia collessi*	Australia [ANIC]	–
	*Bixinia winkleri*	Australia, Queenslad [ANIC]	–
	*Comoromyia* sp. 1	Madagascar, Andringitra [MNHN]	–
	*Comoromyia* sp. 2	Madagascar, Andringitra [MNHN]	–
	Kinabalumyia pinax*	Malaysia, Sabah [NHMUK]	–
	Macrotarsina longimana*	Croatia [MZUR]	–
	*Malayia fuscinervis*	Malaysia, Pahang and Philippines, Palawan [NHMD]	–
	Marshallicona quitu*	Ecuador [NHMD and MZUR]	–
	Maurhinophora indoceanica*	Mauritius [NHMUK]	–
Rhinophoridae	Melanomyiodes capensis*	South Africa, Western Cape [NHMUK]	–
	*Melanophora asetosa*	Israel [TAU]	Pape (1986) Cerretti and Pape (2009)
	*Melanophora basilewskyi*	Kenya [NHMD, TAU, MZUR]	Crosskey (1977) Pape (1986) Cerretti and Pape (2009)
	*Melanophora chia*	Italy, Sardinia [MZUR]	Cerretti and Pape (2009)
	*Melanophora roralis*	Italy: Latium and Veneto [MZUR]	Crosskey (1977) Pape (1986) Pape (1998) Cerretti and Pape (2009)
	*Metoplisa carbonaria*	Israel [MZUR and TAU]	–
	Neotarsina andina*	Peru [NHMD]	–
	Neotarsina caraibica*	Trinidad and Tobago [NHMD and NMHUK]	–
	*Oplisa tergestina*	Italia: Sicilia, Trentino-Alto Adige [MZUR]	Tschorsnig (1985) Pape (1986)
	*Parazamimus congolensis*	Burundi, Kayanza [NMB]	–
	*Paykullia insularis*	France, Corse [NHMD]	–
	*Paykullia maculata*	Czech Republic [NHMD]	Tschorsnig (1985) Pape (1986)
	*Phyto adolescens*	Italy, Sicilia [NHMD]	–
	*Phyto angustifrons*	Italy, Marche [TAU]	–
	*Queximyia flavipes*	South Africa: Eastern Cape and KwaZulu-Natal [NHMD]	Crosskey (1977) Pape (1986)
	*Rhinodonia antiqua*	New Caledonia [MNHN]	–
	*Rhinodonia flavicera*	New Caledonia [INHS]	–
	*Rhinomorinia sarcophagina*	Italy, Latium [MZUR]	Tschorsnig (1985) Pape (1986)
	*Rhinopeza gracilis*	New Guinea [ANIC]	–
	*Rhinophora lepida*	Italy, Veneto [MZUR]	–
	Shannoniella cuspidata*	Brazil, São Paulo [CNC]	Nihei et al. (2016)
	*Shannoniella setinervis*	Brazil, São Paulo [CNC]	Nihei et al. (2016)
	Stevenia deceptoria*	Italy, Sicily [MZUR]	–
	*Stevenia palermitana*	Italy, Sicily [MZUR]	Cerretti and Pape (2007)
	Tricogena rubricosa*	Morocco [NHMD], Tunisia [NHMD]	–
	*Tromodesia angustifrons*	Israel [TAU], Greece [NHMD]	Kugler (1978) Pape (1986)
	*Trypetidomima fusca*	–	Nihei and Andrade (2014)
	Trypetidomima lutea*	Brasil, Rio de Janeiro [USNM]	–
	*Ventrops aethiopicus*	Ethiopia [TAU]	Cerretti and Pape (2012)
	*Ventrops freidbergi*	Tanzania [TAU]	Cerretti and Pape (2012)
	*Ventrops hannemariae*	Kenya [TAU], Malawi [TAU]	Pape (1987)
	*Ventrops incisus*	Tanzania [NHMD]	Pape (1987)
	*Ventrops intermedius*	Tanzania [TAU]	Pape (1987)
	*Ventrops milichioides*	Tanzania and Kenya [TAU]	Crosskey (1977) Tschorsnig (1985) Pape (1986) Pape (1987)
	*Ventrops stuckenbergi*	Namibia and South Africa [MZUR]	Cerretti and Pape (2012)

The data matrix was produced in Mesquite version 3.03 (Maddison and Maddison 2015). Inapplicable or unknown states were coded as ‘–’ and ‘?’, respectively (Table 3). Cladistic analyses were conducted with TNT version 1.5 (Goloboff and Catalano 2016). Heuristic searches were run under equal weights and under implied weighting (k-values: 3–10, 15, 20), with the ‘traditional search’ option under the following settings: General RAM of 1 GB, memory set to hold 1,000,000 trees, setting 1000 replicates with tree bisection-reconnection (TBR) branch swapping and saving 1000 trees per replicate. Multistate characters were treated as unordered. Character state changes (apomorphies) were optimised in WinClada version 1.00.08 (Nixon 2002) on the fittest tree obtained with a k-value of 4, using the unambiguous transformation algorithm. We chose the term “global apomorphies” for the uncontradicted and unreversed apomorphic character states, whereas “local apomorphies” was used for the homoplasious character states due to convergence or reversal.

**Table 3.** Data matrix (outgroups in grey font).

	**1**	**2**	**3**	**4**	**5**	**6**	**7**	**8**	**9**	**10**	**11**	**12**	**13**	**14**	**15**	**16**	**17**	**18**	**19**	**20**	**21**	**22**	**23**	**24**
***Musca* spp.**	1	0	0	0	1	0	0	0	0	–	–	0	0	0	0	0	0	–	1	0	0	0	1	0
***Amenia* sp.**	1	0	0	0	0	0	0	0	0	0	–	0	0	0	1	0	0	–	1	0	0	0	0	0
***Bengalia* sp.**	1	0	0	0	0	0	0	?	0	?	?	0	0	0	0	0	0	–	1	0	1	0	3	–
***Calliphora vomitoria***	1	0	0	0	0	0	0	0	0	1	0	0	0	0	0	0	0	–	0	0	0	1	0	0
***Melinda gentilis***	1	0	0	0	0	0	0	0	0	1	0	0	0	?	0	0	0	–	0	?	2	0	0	0
***Lucilia sericata***	1	0	0	0	0	0	0	0	0	?	0	0	0	0	0	0	0	–	0	0	0	0	0	0
***Cuterebra austeni***	1	0	0	0	0	0	0	0	0	0	–	0	0	0	1	0	0	–	–	1	2	–	4	–
***Pollenia paupera***	1	0	0	0	0	0	0	0	0	1	0	0	0	0	1	0	0	–	0	0	0	0	1	0
***Eurychaeta muscaria***	1	1	0	0	0	1	0	0	1	1	1	0	0	0	0	0	1	1	1	0	0	0	0	0
***Rhyncomya impavida***	0	0	0	0	0	0	0	0	0	1	1	0	0	0	0	0	0	–	0	0	0	1	1	0
***Macquartia tenebricosa***	0	1	0	0	0	0	0	0	0	1	1	0	0	0	0	0	0	–	0	0	0	0	1	0
***Acompomintho lobata***	0	0	0	0	1	0	0	0	1	1	0	0	0	0	0	0	1	1	0	0	0	0	2	–
***Apomorphyto inbio***	0	0	0	0	0	0	1	0	0	0	–	0	0	0	0	0	0/1	0	0	0	0	0	2	–
***Aporeomyia* sp.**	0	0	0	0	1	2	?	0	0	?	–	0	0	1	0	0	0	–	0	1	0	0	2	–
***Axinia disjuncta***	–	–	1	0	?	?	0	?	1	?	0	0	0	0	1	0	0	–	0	0	?	0	4	–
***Axinia lucaris***	0	0	0	0	1	0	0	0	1	1	0	0	0	1	1	0	1	0	0	0	0	0	2	–
***Axinia miranda***	0	0	0	0	?	0	0	?	2	?	0	0	0	1	1	0	0	–	0	0	?	0	3	–
***Azaisia* sp.**	0	0	0	0	0	0	0	0	1	1	0	0	0	0	0	0	0	?	0	0	0	0	2	–
***Baniassa fascipennis***	0	0	0	0	0	0	1	0	0	1	0	0	0	0	0	0	1	0	0	0	0	0	2	–
***Bezzimyia barbarista***	0	0	0	1	1	0	1	1	0	0	–	1	1	1	0	0	0	–	0	0	0	0	2	–
***Bezzimyia bisecta***	0	0	0	0	1	0	0	0	0	0	–	0	0	1	0	0	0	–	0	1	0	0	2	–
***Bezzimyia busckii***	0	0	0	0	1	0	1	1	0	0	–	1	1	1	0	0	0	–	0	0	0	0	2	–
***Bezzimyia hansoni***	0	0	0	0	1	0	0	0	0	0	–	0	0	1	0	0	0	–	0	1	0	0	2	–
***Bezzimyia yepezi***	0	0	0	0	0	0	0	0	0	0	?	0	0	0	0	0	0	–	0	0	0	0	2	–
***Bixinia collessi***	0	0	0	0	1	0	0	?	0	0	–	0	0	0	1	0	0	–	0	0	0	0	2	–
***Bixinia winckleri***	0	0	0	0	1	0	0	0	0	0	–	0	0	0	1	0	0	–	0	0	0	0	2	–
***Comoromyia* sp. 1**	0	0	0	0	0	0	0	0	1	1	0	0	0	0	0	0	0/1	0	0	0	0	0	2	–
***Comoromyia* sp. 2**	0	0	0	0	0	0	0	0	1	1	0	0	0	0	0	0	0	–	0	0	0	0	2	–
***Kinabalumyia pinax***	0	0	0	0	1	2	0	0	1	1	0	0	0	0	1	0	0	–	?	0	1	0	2	–
***Macrotarsina longimana***	0	0	0	0	0	0	0	0	1	1	0	0	0	0	0	0	0	?	0	0	0	0	2	–
***Malayia fuscinervis***	0	0	0	0	1	0	0	0	1	1	0	0	1	1	0	0	0/1	0	0/1	0	0	0	1	0
***Marshallicona quitus***	1	1	0	0	0	0	0	?	1	?	1	0	0	0	0	0	0	–	0	0	0	0	2	–
***Maurhinophora indoceanica***	0	0	0	0	0	0	0	0	?	1	0	0	1	1	0	0	0	–	0	0	2	0	1	1
***Melanomyiodes capensis***	0	0	0	0	0	0	0	0	0	1	0	0	0	0	0	0	0	–	0	0	0	0	2	–
***Melanophora asetosa***	1	1	0	0	0	0	0	0	1	1	1	0	0	0	0	0	0	–	1	1	?	0	2	–
***Melanophora basilewskyi***	1	1	0	0	0	0	0	?	1	1	1	0	0	0	0	0	0	–	1	1	?	0	3	–
***Melanophora chia***	1	1	0	0	0	0	0	0	1	1	1	0	0	0	0	0	0	–	1	0	0	0	2	–
***Melanophora roralis***	1	1	0	0	0	0	0	0	1	1	1	0	0	0	0	0	0	–	1	0	0	0	2	–
***Metoplisa carbonaria***	0	0	0	0	0	0	0	0	1	1	0/1	0	0	0	0	0	0	–	0	0	0	0	2	–
***Neotarsina andina***	0	0	0	0	0	0	0	1	0	0	?	0	0	0	0	0	0	–	0	0	1	0	2	–
***Neotarsina caraibica***	0	0	0	0	0	0	0	1	0	0	?	0	0	0	0	0	0	–	0	0	1	0	2	–
***Oplisa tergestina***	1	0	0	0	0	0	0	0	1	1	0	0	0	0	0	0	0	–	0	0	0	0	1	1
***Parazamimus congolensis***	0	0	0	0	0	0	1	0	1	1	0	0	0	0	0	0	1	0	0	0	0	0	2	–
***Paykullia insularis***	0	0	0	0	0	0	0	0	0	1	0	0	0	0	0	0	0	–	0	0	0	0	2	–
***Paykullia maculata***	0	0	0	0	0	0	0	0	1	1	0	0	0	0	0	0	0	–	0	0	0	0	2	–
***Phyto adolescens***	0	0	0	0	0	0	1	0	0	1	0	0	0	0	0	0	1	0	0	0	0	0	1	0
***Phyto angustifrons***	0	0	0	0	0	0	1	0	0	1	0	0	0	0	0	0	1	1	0	0	0	0	1	0
***Queximyia flavipes***	1	0	0	0	0	0	0	0	0	1	0	0	0	0	0	0	0	–	0	0	0	0	1	1
***Rhinodonia antiqua***	0	0	0	0	0	0	0	0	1	1	0	0	0	0	1	0	0	–	0	0	0	0	2	–
***Rhinodonia flavicera***	0	0	0	0	1	0	0	?	1	?	0	0	0	0	1	0	0	–	0	0	0	0	2	–
***Rhinomorinia sarcophagina***	1	0	0	0	0	0	0	0	0	1	0	0	0	0	0	0	0	–	0	0	0	0	1	1
***Rhinopeza gracilis***	0	0	0	0	1	0	0	?	0	?	?	0	0	0	0	0	0	–	0	1	0	0	3	–
***Rhinophora lepida***	0	0	0	0	0	0	1	0	0	1	0	0	0	0	0	0	1	0	1	0	0	0	2	–
***Shannoniella cuspidata***	0	0	0	0	1	1	0	0	2	2	1	1	1	1	0	0	0	–	0	0	0	0	2	–
***Shannoniella setinervis***	0	0	0	0	1	1	0	0	2	?	1	1	1	1	0	0	0	–	0	0	0	0	2	–
***Stevenia deceptoria***	0	0	0	0	0	0	0	0	1	1	0	0	0	0	0	0	1	1	0	0	0	0	0	1
***Stevenia palermitana***	0	0	0	0	0	0	0	0	0	1	0	0	0	0	0	0	1	1	0	0	0	0	0	1
***Tricogena rubricosa***	0	0	0	0	0	0	0	0	1	1	0	0	0	0	0	0	1	1	0	0	0	0	0	1
***Tromodesia angustifrons***	1	0	0	0	0	0	0	0	0	1	0	0	0	0	0	0	0	–	0	0	0	0	2	–
***Trypetidomima fusca***	1	0	0	0	0	0	0	0	0	0	–	0	0	0	0	0	0	–	0	0	0	0	2	–
***Trypetidomima lutea***	1	0	0	0	0	0	0	0	0	0	–	0	0	0	0	0	0	–	0	0	0	0	2	–
***Ventrops aethiopicus***	0	0	0	0	0	0	0	?	1	?	1	0	0	0	0	0	1	0	0	0	0	0	1	0
***Ventrops freidbergi***	0	0	0	0	0	0	0	?	1	?	1	0	0	0	0	0	1	0	1	0	0	0	1	0
***Ventrops hannemariae***	0	0	0	0	0	0	0	0	1	1	1	0	0	0	0	0	1	0	0	0	0	0	2	–
***Ventrops incisus***	1	0	0	0	0	0	0	0	1	1	1	0	0	0	0	1	0	–	0	0	0	0	2	–
***Ventrops intermedius***	0	0	0	0	0	0	0	0	1	1	1	0	0	0	0	0	1	0	0	0	0	0	2	–
***Ventrops milichioides***	1	0	0	0	0	0	0	0	1	1	1	0	0	0	0	1	0	–	0	0	0	0	2	–
***Ventrops stuckenbergi***	0	0	0	0	0	0	0	?	1	?	1	0	0	0	0	0	1	1	1	0	0	0	1	0
	**25**	**26**	**27**	**28**	**29**	**30**	**31**	**32**	**33**	**34**	**35**	**36**	**37**	**38**	**39**	**40**	**41**	**42**	**43**	**44**	**45**	**46**		
***Musca* spp.**	0	0	0	0	1	0	0	0	0	0	0	0	0	0	0	0	0	0	1	–	0	0		
***Amenia* sp.**	0	0	0	0	0	2	0	0	0	0	1	0	0	0	0	0	0	0	0	0	0	0		
***Bengalia* sp.**	1	0	0	0	1	2	0	?	0	0	0	0	0	?	0	0	?	0	0	0	0	0		
***Calliphora vomitoria***	0	0	?	0	0	2	0	0	0	0	0	0	0	0	0	0	0	0	0	0	0	0		
***Melinda gentilis***	0	0	0	0	1	2	0	0	0	0	0	0	?	0	0	0	0	0	0	0	0	0		
***Lucilia sericata***	0	0	0	0	0	2	0	0	0	0	0	0	?	0	0	0	0	0	0	0	0	0		
***Cuterebra austeni***	1	0	0	1	1	2	0	0	0	0	0	1	0	0	0	0	0	0	1	–	0	0		
***Pollenia paupera***	0	0	0	0	1	1	0	0	0	0	0	0	0	0	0	0	0	0	0	0	0	0		
***Eurychaeta muscaria***	0	0	0	0	1	2	0	0	0	0	1	0	0	0	0	0	0	0	0	0	0	0		
***Rhyncomya impavida***	0	0	?	0	0	2	0	0	0	0	0	1	0	0	0	0	0	0	0	0	0	0		
***Macquartia tenebricosa***	0	2	0	0	1	2	0	0	0	0	0	0	0	0	0	0	0	0	0	0	0	0		
***Acompomintho lobata***	0	1	0	0	1	1	0	0	0	0	0	1	0	0	0	0	0	0	0	0	0	0		
***Apomorphyto inbio***	1	1	0	0	1	1	0	1	0	0	0	1	0	0	0	0	0	0	1	–	0	0		
***Aporeomyia* sp.**	1	1	0	0	1	0	0	?	0	0	0	1	0	?	0	0	?	0	1	–	1	–		
***Axinia disjuncta***	1	1	0	0	1	0	0	?	0	1	0	1	0	?	0	0	?	0	1	–	1	–		
***Axinia lucaris***	1	1	0	0	1	0	0	1	0	1	0	1	0	0	0	0	0	0	1	–	1	–		
***Axinia miranda***	1	1	0	0	1	0	?	?	0	1	0	1	0	?	0	0	?	0	1	–	1	–		
***Azaisia* sp.**	1	1	0	0	1	1	1	?	0	0	0	1	0	0	0	0	0	0	0	0	0	0		
***Baniassa fascipennis***	0/1	1	0	0	0	2	1	1	0	0	0	1	0	0	0	0	0	0	0	0	0	0		
***Bezzimyia barbarista***	1	1	0	0	1	1	1	1	0	0	0	1	0	0	0	0	0	1	1	–	–	–		
***Bezzimyia bisecta***	1	1	0	1	1	1	0	1	0	0	1	1	0	?	0	0	?	0	0	0	–	–		
***Bezzimyia busckii***	1	1	0	0	1	1	1	1	0	0	0	1	0	0	0	0	0	1	1	–	–	–		
***Bezzimyia hansoni***	1	1	0	1	1	1	0	?	0	0	1	1	0	2	0	0	?	0	0	0	–	–		
***Bezzimyia yepezi***	1	1	0	0	1	2	0	0	0	0	1	1	0	0	0	0	0	0	0	0	–	–		
***Bixinia collessi***	1	1	1	0	1	1	0	?	0	1	1	1	0	0	0	0	0	0	1	–	0	1		
***Bixinia winckleri***	1	1	1	0	1	1	1	1	0	1	1	1	0	0	0	0	0	0	1	–	0	1		
***Comoromyia* sp. 1**	2	1	0	1	1	1	0	0	0	0	0	1	0	0	0	0	0	0	0	0	0	0		
***Comoromyia* sp. 2**	2	1	0	1	1	1	0	0	0	0	0	1	0	0	0	0	0	0	0	0	0	0		
***Kinabalumyia pinax***	1	1	0	0	1	0	0	?	0	0	0	1	0	?	0	0	?	0	1	–	1	–		
***Macrotarsina longimana***	1	1	0	0	1	1	1	0	0	0	0	1	0	0	0	0	0	0	0	0	0	0		
***Malayia fuscinervis***	1	1	0	0	1	1	0	0	0	0	1	1	0	0	0	0	0	0	0	0	0	0		
***Marshallicona quitus***	1	1	0	0	1	0	0	?	0	0	0	1	0	?	0	0	?	2	1	–	0	0		
***Maurhinophora indoceanica***	2	1	0	0	0	2	?	0	0	0	0	1	0	0	0	?	0	1	0	1	0	0		
***Melanomyiodes capensis***	1	1	0	0	1	1	0	0	0	0	0	1	0	0	0	0	0	0	0	0	0	0		
***Melanophora asetosa***	1	1	0	1	1	1	0	0	0	0	0	1	0	0	0	0	0	0	0	0	–	–		
***Melanophora basilewskyi***	1	1	0	1	1	0	0	?	0	1	0	1	0	?	0	0	0	1	0	1	–	–		
***Melanophora chia***	1	1	0	0	1	1	0	0	0	0	0	1	0	0	1	0	1	0	0	0	0	0		
***Melanophora roralis***	1	1	0	0	1	1	0	0	0	0	0	1	0	0	1	0	1	0	0	0	0	0		
***Metoplisa carbonaria***	1	1	0	0	1	1	0	0	0	0	0	1	0	0	0	0	0	0	0	0	0	0		
***Neotarsina andina***	1	1	0	0	1	0	1	1	1	0	0	1	0	0	0	0	0	2	1	–	0	–		
***Neotarsina caraibica***	1	1	0	0	1	1	1	1	1	0	0	1	0	0	0	0	0	0	1	–	0	–		
***Oplisa tergestina***	0	1	0	0	1	1	0	0	0	0	0	1	0	0	0	0	0	0	0	0	0	0		
***Parazamimus congolensis***	1	1	0	0	0	1	0	0	0	0	0	1	0	0	0	0	0	0	0	0	0	0		
***Paykullia insularis***	1	1	0	0	1	1	0	0	0	0	0	1	0	0	0	0	0	0	0	0	0	0		
***Paykullia maculata***	1	1	0	0	1	1	0	0	0	0	0	1	0	0	0	0	0	0	0	0	0	0		
***Phyto adolescens***	0	1	0	0	0	1	0	0	0	0	0	1	0	0	0	0	0	0	0	0	0	0		
***Phyto angustifrons***	0	1	0	0	0	1	0	0	0	0	0	1	0	0	0	0	0	0	0	0	0	0		
***Queximyia flavipes***	2	1	0	0	0	1	0	0	0	0	0	1	0	0	0	0	0	0	0	0	0	0		
***Rhinodonia antiqua***	0	1	0	0	1	1	0	0	0	0	0	1	0	0	0	0	0	0	1	–	1	–		
***Rhinodonia flavicera***	0	1	0	0	1	1	0	?	0	0	0	1	0	?	0	0	?	0	1	–	1	–		
***Rhinomorinia sarcophagina***	0	1	0	0	1	1	0	0	0	0	0	1	0	0	0	0	0	0	0	0	0	0		
***Rhinopeza gracilis***	1	1	0	0	1	0	0	?	0	1	0	1	0	?	0	0	?	0	0	0	1	–		
***Rhinophora lepida***	1	1	0	0	1	1	0	0	0	0	0	1	0	0	0	0	0	0	0	0	0	0		
***Shannoniella cuspidata***	1	1	0	1	1	0	1	1	0	0	0	1	1	0	0	1	0	1	0	1	0	0		
***Shannoniella setinervis***	1	1	0	1	1	0	1	?	0	0	0	1	1	0	0	1	?	1	0	1	0	0		
***Stevenia deceptoria***	1	1	0	0	1	1	0	0	0	0	0	1	0	0	0	0	0	0	0	0	0	0		
***Stevenia palermitana***	0	1	0	0	1	1	0	0	0	0	0	1	0	0	0	0	0	0	0	0	0	0		
***Tricogena rubricosa***	1	1	0	0	1	1	0	0	0	0	0	1	0	0	0	0	0	0	0	0	0	0		
***Tromodesia angustifrons***	1	1	0	0	1	1	0	0	0	0	0	1	0	0	0	0	0	0	0	0	0	0		
***Trypetidomima fusca***	0	1	0	0	1	1	0	1	0	0	0	1	1	0	0	1	0	2	0	0	0	0		
***Trypetidomima lutea***	1	1	0	0	1	1	0	1	0	0	0	1	1	0	0	1	0	2	0	0	0	0		
***Ventrops aethiopicus***	1	1	0	0	1	1	0	0	0	0	0	1	0	?	0	0	?	0	0	0	–	–		
***Ventrops freidbergi***	1	1	0	0	1	1	0	?	0	0	0	1	0	?	0	0	?	0	0	0	–	–		
***Ventrops hannemariae***	1	1	0	0	1	1	0	0	0	0	0	1	0	0	0	0	0	0	0	0	0	0		
***Ventrops incisus***	1	1	0	0	1	1	0	0	0	0	0	1	0	0	0	0	?	0	0	0	0	0		
***Ventrops intermedius***	1	1	0	0	1	1	0	0	0	0	0	1	0	0	0	0	?	0	0	0	0	0		
***Ventrops milichioides***	1	1	0	0	1	1	0	0	0	0	0	1	0	0	0	0	0	0	0	0	0	0		
***Ventrops stuckenbergi***	1	1	0	0	1	1	0	0	0	0	0	1	0	?	0	0	?	0	0	0	0	0		
	**47**	**48**	**49**	**50**	**51**	**52**	**53**	**54**	**55**	**56**	**57**	**58**	**59**	**60**	**61**	**62**	**63**	**64**	**65**	**66**	**67**	**68**		
***Musca* spp.**	0	1	0	0	0	1	?	?	?	?	?	?	?	–	0	0	0	–	1	0	1	–		
***Amenia* sp.**	0	1	0	0	0	0	0	0	0	0	0	0	0	0	0	0	0	–	0	0	1	0		
***Bengalia* sp.**	0	1	0	0	1	1	1	1	0	–	–	–	–	0	0	0	0	–	1	0	1	0		
***Calliphora vomitoria***	0	1	0	0	1	0	0	0	0	0	0	0	0	0	0	0	0	0	0	0	1	0		
***Melinda gentilis***	0	1	0	0	1	0	0	0	0	0	0	0	0	0	0	0	0	–	1	0	1	0		
***Lucilia sericata***	0	1	0	0	1	0	0	0	0	?	?	?	?	0	0	0	0	–	1	0	1	0		
***Cuterebra austeni***	0	1	0	0	1	1	1	1	0	1	0	0	0	1	0	0	0	–	0	0	1	–		
***Pollenia paupera***	0	0	0	0	1	1	?	?	?	?	?	?	?	0	0	0	0	–	0	0	1	0		
***Eurychaeta muscaria***	0	1	0	0	0	0	0	0	0	0	?	0	0	0	0	0	0	–	0	0	1	1		
***Rhyncomya impavida***	0	1	0	0	1	1	0	0	0	?	3	0	0	0	0	0	0	0	0	0	1	0		
***Macquartia tenebricosa***	0	1	0	0	0	0	?	?	?	?	?	?	?	0	0	0	0	–	0	0	1	0		
***Acompomintho lobata***	0	0	0	0	0	0	0	0	0	?	?	0	?	0	0	0	0	0	1	0	1	?		
***Apomorphyto inbio***	0	1	0	0	1	1	0	1	0	0	0	0	0	0	0	0	0	0	1	1	1	0		
***Aporeomyia* sp.**	0	1	0	1	0	0	0	1	0	0	0	1	0	0	1	0	0	1	0	0	1	0		
***Axinia disjuncta***	0	1	0	0	1	1	0	0	0	0	0	0	0	0	1	1	0	0	1	1	0	1		
***Axinia lucaris***	0	1	0	0	1	1	0	0	1	0	0	0	1	0	1	1	0	0	0	1	1	1		
***Axinia miranda***	0	1	0	0	1	1	0	0	0	0	0	0	1	0	1	1	0	0	1	1	1	?		
***Azaisia* sp.**	0	1	0	0	0	0	0	0	0	0	0	0	0	0	0	0	0	0	1	1	1	1		
***Baniassa fascipennis***	0	0	0	0	1	0	0	0	0	0	0	0	0	0	0	0	0	1	0	0	1	0		
***Bezzimyia barbarista***	1	–	0	0	1	1	0	0	0	0	0	0	0	0	0	0	0	0	0	1	1	0		
***Bezzimyia bisecta***	1	–	1	0	0	0	0	1	0	0	0	0	0	0	0	0	0	0	1	1	1	0		
***Bezzimyia busckii***	1	–	0	0	1	1	0	0	0	0	1	0	0	0	0	0	0	0	0	1	1	0		
***Bezzimyia hansoni***	1	–	1	0	0	0	0	1	0	0	0	0	0	0	0	0	0	0	1	1	1	0		
***Bezzimyia yepezi***	1	–	0	0	0	0	0	0	0	0	0	1	0	0	0	0	0	1	1	1	1	0		
***Bixinia collessi***	0	1	0	0	1	1	0	0	0	1	0	0	0	0	0	0	0	0	1	0	1	0		
***Bixinia winckleri***	0	1	0	1	1	1	0	0	0	1	0	0	0	0	0	0	0	0	1	0	1	0		
***Comoromyia* sp. 1**	0	1	0	0	0	0	0	0	0	0	0	0	0	0	0	0	1	1	0	0	1	0		
***Comoromyia* sp. 2**	0	1	0	0	1	0	0	0	0	0	0	0	0	0	0	0	1	1	0	0	1	0		
***Kinabalumyia pinax***	0	1	0	1	0	0	1	0	0	0	0	1	1	0	1	0	0	0	0	0	1	0		
***Macrotarsina longimana***	0	1	0	0	0	0	0	0	0	0	0	0	0	0	0	0	0	0	1	0	1	1		
***Malayia fuscinervis***	0	1	0	1	0	0	0	0	0	0	0	0	0	0	0	0	0	0	0	0	1	0		
***Marshallicona quitus***	0	1	1	0	1	1	0	0	0	1	2	0	0	0	0	0	0	0	1	0	1	1		
***Maurhinophora indoceanica***	0	1	0	0	0	0	?	?	?	?	?	?	?	?	?	?	?	?	?	?	?	?		
***Melanomyiodes capensis***	0	0	0	0	1	0	0	0	0	0	0	0	0	0	0	0	0	1	1	0	1	0		
***Melanophora asetosa***	1	–	0	0	1	0	0	0	0	0	0	0	0	0	0	0	0	0	0	0	1	0		
***Melanophora basilewskyi***	1	–	0	0	1	1	0	0	0	0	0	0	0	0	1	0	0	1	0	1	1	0		
***Melanophora chia***	0	0	0	0	0	0	0	0	0	0	0	0	0	0	0	0	0	0	0	0	1	0		
***Melanophora roralis***	0	0	0	0	0	0	0	0	0	0	0	0	0	0	0	0	0	0	0	0	1	0		
***Metoplisa carbonaria***	0	1	0	0	1	0	0	0	0	0	2	0	0	0	0	0	0	0	1	0	0	0		
***Neotarsina andina***	0	1	0	0	1	1	0	0	0	0	0	0	1	0	0	0	0	0	0	1	1	0		
***Neotarsina caraibica***	0	1	0	0	1	1	0	0	0	0	1	0	1	0	0	0	0	0	0	1	1	0		
***Oplisa tergestina***	0	1	0	0	0	0	0	0	0	0	0	0	0	0	0	0	0	0	1	0	1	1		
***Parazamimus congolensis***	0	1	0	1	1	0	0	0	0	0	0	0	0	0	0	0	0	0	0	0	1	0		
***Paykullia insularis***	0	0	0	0	0	0	1	1	0	0	0	0	0	0	0	0	0	0	0	0	1	0		
***Paykullia maculata***	0	0	0	0	0	0	1	1	0	0	0	0	0	0	0	0	0	0	0	0	1	0		
***Phyto adolescens***	0	0	0	0	0	0	0	0	0	0	0	0	0	0	0	0	0	0	0	0	1	0		
***Phyto angustifrons***	0	0	0	0	0	0	0	0	0	0	0	0	0	0	0	0	0	0	0	0	1	0		
***Queximyia flavipes***	0	1	0	0	0	0	0	0	0	?	3	0	0	0	0	0	0	0	1	0	0	0		
***Rhinodonia antiqua***	0	1	0	0	1	0	0	0	0	0	0	0	0	1	1	0	0	0	0	1	1	0		
***Rhinodonia flavicera***	0	1	0	0	1	0	0	0	0	0	0	0	0	1	1	0	0	0	0	1	1	0		
***Rhinomorinia sarcophagina***	0	1	0	0	0	0	0	0	0	0	0	0	0	0	0	0	0	1	1	0	0	0		
***Rhinopeza gracilis***	0	1	0	0	0	0	0	0	0	?	?	?	?	0	0	0	0	0	0	1	1	0		
***Rhinophora lepida***	0	0	0	0	0	0	0	0	0	1	0	0	0	0	0	0	0	1	0	0	1	0		
***Shannoniella cuspidata***	0	1	1	0	1	1	0	0	0	0	2	0	0	0	0	0	0	0	1	1	1	0		
***Shannoniella setinervis***	0	1	1	0	0	0	0	0	0	0	2	0	0	0	0	0	0	0	1	1	1	0		
***Stevenia deceptoria***	0	0	0	0	0	0	0	0	0	0	0	0	0	0	0	0	0	0	1	0	1	1		
***Stevenia palermitana***	0	0	0	0	0	0	0	0	0	0	0	0	0	0	0	0	0	0	1	0	1	1		
***Tricogena rubricosa***	0	1	0	0	0	0	0	0	0	0	0	0	0	0	0	0	0	0	1	0	1	1		
***Tromodesia angustifrons***	0	1	0	0	0	0	0	0	0	0	0	0	0	0	0	0	0	1	1	0	1	0		
***Trypetidomima fusca***	0	1	1	0	0	0	0	0	0	?	3	?	0	0	0	0	0	0	1	1	1	?		
***Trypetidomima lutea***	0	1	1	0	0	0	0	0	0	?	3	?	0	0	0	0	0	0	1	1	1	?		
***Ventrops aethiopicus***	1	–	0	0	0	0	0	0	0	0	0	0	0	0	0	0	0	0	1	0	1	0		
***Ventrops freidbergi***	1	–	0	0	0	0	0	0	0	0	0	0	0	0	0	0	0	0	1	0	1	0		
***Ventrops hannemariae***	0	1	0	0	0	0	0	0	0	1	0	0	0	0	0	0	0	0	1	0	1	0		
***Ventrops incisus***	0	1	0	0	0	0	0	0	0	1	2	0	0	0	0	0	0	0	1	0	1	0		
***Ventrops intermedius***	0	1	0	0	0	0	0	0	0	1	0	0	0	0	0	0	0	0	1	0	1	0		
***Ventrops milichioides***	0	1	0	0	0	0	0	0	0	1	2	0	0	0	0	0	0	0	1	0	1	0		
***Ventrops stuckenbergi***	0	0	0	0	0	0	0	0	0	1	0	0	0	0	0	0	0	0	1	0	1	0		
	**69**	**70**	**71**	**72**	**73**	**74**	**75**	**76**	**77**	**78**	**79**	**80**	**81**	**82**	**83**	**84**	**85**	**86**	**87**	**88**	**89**	**90**	**91**	
***Musca* spp.**	1	–	–	–	–	–	–	–	–	–	–	–	–	?	0	?	0	0	0	0	0	0	1	
***Amenia* sp.**	0	0	0	0	0	1	–	–	–	–	0	0	0	?	?	?	?	?	?	?	?	?	?	
***Bengalia* sp.**	0	0	0	1	0	1	–	–	–	–	–	–	–	?	0	?	0	0	0	0	0	0	1	
***Calliphora vomitoria***	0	0	0	0	0	0	0	0	0	0	0	0	0	0	0	0	0	0	0	0	0	0	0	
***Melinda gentilis***	0	0	0	0	0	1	–	–	–	–	0	0	0	0	1	?	0	0	0	0	1	0	0	
***Lucilia sericata***	0	0	0	0	0	0	0	0	0	0	0	0	0	?	0	?	0	0	0	0	0	0	1	
***Cuterebra austeni***	–	0	0	0	0	1	–	–	–	–	0	0	0	?	?	?	?	?	?	?	?	?	?	
***Pollenia paupera***	0	0	0	0	0	0	1	0	0	0	0	0	0	?	?	?	0	0	0	0	0	0	0	
***Eurychaeta muscaria***	0	0	0	0	0	0	0	0	0	0	0	0	0	?	?	?	?	?	?	?	0	?	?	
***Rhyncomya impavida***	0	0	1	0	0	0	0	0	0	0	0	0	0	0	?	0	?	?	?	?	?	?	?	
***Macquartia tenebricosa***	–	–	–	–	–	–	–	–	–	–	–	–	–	?	?	?	?	?	?	?	?	?	?	
***Acompomintho lobata***	0	0	0	0	0	0	1	0	0	0	1	0	0	0	?	?	?	?	?	?	?	?	?	
***Apomorphyto inbio***	0	0	0	0	0	0	1	0	0	0	0	0	0	0	?	?	?	?	?	?	?	?	?	
***Aporeomyia* sp.**	1	0	0	0	0	1	–	–	–	–	0	0	0	?	?	?	?	?	?	?	?	?	?	
***Axinia disjuncta***	1	0	1	0	0	0	0	0	0	0	0	0	0	?	?	?	?	?	?	?	?	?	?	
***Axinia lucaris***	1	0	1	0	0	0	0	0	0	0	0	0	0	0	?	?	?	?	?	?	?	?	?	
***Axinia miranda***	?	1	?	?	0	0	0	0	0	0	0	0	0	?	?	?	?	?	?	?	?	?	?	
***Azaisia* sp.**	0	0	0	0	1	0	0	0	0	0	1	0	?	0	?	?	?	?	?	?	?	?	?	
***Baniassa fascipennis***	0	0	0	0	0	0	0	0	0	0	0	0	0	0	?	?	?	?	?	?	?	?	?	
***Bezzimyia barbarista***	0	0	0	0	0	1	?	?	?	?	0	0	0	1	?	?	?	?	?	?	?	?	?	
***Bezzimyia bisecta***	1	0	1	0	0	1	?	?	?	?	0	0	0	0	?	?	?	?	?	?	?	?	?	
***Bezzimyia busckii***	0	0	0	0	0	0	1	0	0	0	0	0	0	?	?	?	?	?	?	?	?	?	?	
***Bezzimyia hansoni***	1	0	1	0	0	1	?	?	?	?	0	0	0	0	?	?	?	?	?	?	?	?	?	
***Bezzimyia yepezi***	1	0	1	0	0	1	?	?	?	?	0	0	0	1	1	1	1	1	0	1	?	0	1	
***Bixinia collessi***	1	0	0	0	0	0	0	0	0	0	0	0	0	0	?	?	?	?	?	?	?	?	?	
***Bixinia winckleri***	1	0	0	0	0	0	0	0	0	0	0	0	0	0	?	?	?	?	?	?	?	?	?	
***Comoromyia* sp. 1**	0	0	0	0	0	0	1	0	0	0	0	0	0	0	?	?	?	?	?	?	?	?	?	
***Comoromyia* sp. 2**	0	0	0	0	0	0	1	0	0	0	0	0	0	0	?	?	?	?	?	?	?	?	?	
***Kinabalumyia pinax***	1	0	0	0	0	0	1	1	0	0	0	0	1	1	?	?	?	?	?	?	?	?	?	
***Macrotarsina longimana***	0	0	0	0	0	0	0	0	0	0	1	0	0	0	?	?	?	?	?	?	?	?	?	
***Malayia fuscinervis***	0	0	0	0	0	1	?	?	?	?	0	0	0	1	?	?	?	?	?	?	?	?	?	
***Marshallicona quitus***	1	0	1	0	0	0	0	1	0	0	0	0	0	?	?	?	?	?	?	?	?	?	?	
***Maurhinophora indoceanica***	?	?	?	?	?	?	?	?	?	?	?	?	0	0	?	?	?	?	?	?	?	?	?	
***Melanomyiodes capensis***	?	0	0	0	0	0	0	0	0	0	0	0	0	?	?	?	?	?	?	?	?	?	?	
***Melanophora asetosa***	0	0	1	0	0	0	0	0	0	0	0	0	0	1	?	?	?	?	?	?	?	?	?	
***Melanophora basilewskyi***	1	1	?	?	0	0	0	0	0	0	0	0	0	?	?	?	?	?	?	?	?	?	?	
***Melanophora chia***	0	0	1	0	0	0	0	0	0	0	0	0	0	0	?	?	?	?	?	?	?	?	?	
***Melanophora roralis***	0	0	1	0	0	0	0	0	0	0	0	0	0	1	1	2	1	1	0	1	?	0	1	
***Metoplisa carbonaria***	0	0	0	0/1	1	0	1	0	0	0	1	0	0	0	?	?	?	?	?	?	?	?	?	
***Neotarsina andina***	1	0	0	1	0	0	0	0	0	0	0	0	0	0	?	?	?	?	?	?	?	?	?	
***Neotarsina caraibica***	1	0	0	1	0	0	0	0	0	0	0	0	0	0	?	?	?	?	?	?	?	?	?	
***Oplisa tergestina***	0	0	0	0	1	0	0	0	0	0	1	0	0	0	1	1	1	1	0	1	?	1	1	
***Parazamimus congolensis***	1	0	0	0	0	0	1	0	0	0	0	0	0	0	?	?	?	?	?	?	?	?	?	
***Paykullia insularis***	1	0	0	1	0	0	0	0	0	0	0	0	0	1	1	2	1	1	0	1	?	0	1	
***Paykullia maculata***	0	0	0	1	0	0	0	0	0	0	0	0	0	1	1	2	1	1	0	1	?	0	1	
***Phyto adolescens***	0	0	0	0	0	0	1	0	0	0	0	0	0	0	1	2	1	1	0	1	?	0	1	
***Phyto angustifrons***	0	0	0	0	0	0	1	0	0	0	0	0	0	0	1	2	1	1	0	1	?	0	1	
***Queximyia flavipes***	0	0	1	0	0	0	0	0	0	0	1	0	0	0	?	?	?	?	?	?	?	?	?	
***Rhinodonia antiqua***	0	0	1	0	0	0	0	0	0	0	0	0	0	0	?	?	?	?	?	?	?	?	?	
***Rhinodonia flavicera***	0	0	1	0	0	0	0	0	0	0	0	0	0	?	?	?	?	?	?	?	?	?	?	
***Rhinomorinia sarcophagina***	0	0	0	0	1	0	0	0	0	0	1	0	0	0	1	1	1	1	0	1	?	1	1	
***Rhinopeza gracilis***	0	0	0	0	0	0	1	0	0	0	0	0	0	?	?	?	?	?	?	?	?	?	?	
***Rhinophora lepida***	0	0	1	0	0	0	0	0	0	0	0	0	0	0	1	1	1	1	0	1	?	1	1	
***Shannoniella cuspidata***	0	0	1	0	0	0	0	1	0	0	0	0	0	?	?	?	?	?	?	?	?	?	?	
***Shannoniella setinervis***	0	0	1	0	0	0	0	1	0	0	0	0	0	?	?	?	?	?	?	?	?	?	?	
***Stevenia deceptoria***	0	0	0	0	1	0	1	0	0	0	1	0	0	0	1	1	1	1	0	1	?	1	1	
***Stevenia palermitana***	0	0	0	0	1	0	1	0	0	0	1	0	0	0	1	1	1	1	0	1	?	1	1	
***Tricogena rubricosa***	0	0	0	0	1	0	1	0	0	0	1	0	0	0	1	1	1	1	0	1	?	1	1	
***Tromodesia angustifrons***	0	0	0	0	0	0	0	0	0	0	0	0	0	0	?	?	?	?	?	?	?	?	?	
***Trypetidomima fusca***	0	0	1	0	0	0	0	1	0	0	0	0	0	0	?	?	?	?	?	?	?	?	?	
***Trypetidomima lutea***	0	0	1	0	0	0	0	1	0	0	0	0	0	0	?	?	?	?	?	?	?	?	?	
***Ventrops aethiopicus***	0	0	1	0	0	0	0	0	0	0	0	1	0	?	?	?	?	?	?	?	?	?	?	
***Ventrops freidbergi***	0	0	1	0	0	0	0	0	0	0	0	1	0	?	?	?	?	?	?	?	?	?	?	
***Ventrops hannemariae***	0	0	1	0	0	0	0	1	1	1	0	1	0	0	?	?	?	?	?	?	?	?	?	
***Ventrops incisus***	0	0	1	0	0	0	0	0	0	0	0	1	0	0	?	?	?	?	?	?	?	?	?	
***Ventrops intermedius***	0	0	1	0	0	0	0	1	1	1	0	1	0	0	?	?	?	?	?	?	?	?	?	
***Ventrops milichioides***	0	0	1	0	0	0	0	0	0	0	0	1	0	0	?	?	?	?	?	?	?	?	?	
***Ventrops stuckenbergi***	0	0	1	0	0	0	0	1	1	0	0	1	0	?	?	?	?	?	?	?	?	?	?	
	**92**	**93**	**94**	**95**	**96**	**97**	**98**	**99**									**92**	**93**	**94**	**95**	**96**	**97**	**98**	**99**
***Musca* spp.**	0	0	0	0	0	1	0	–	***Melanophora asetosa***								?	?	?	?	?	?	?	?
***Amenia* sp.**	?	?	?	?	?	?	?	?	***Melanophora basilewskyi***								?	?	?	?	?	?	?	?
***Bengalia* sp.**	0	0	0	?	0	1	0	–	***Melanophora chia***								?	?	?	?	?	?	?	?
***Calliphora vomitoria***	0	0	0	0	0	0	?	?	***Melanophora roralis***								1	0	0	1	1	0	0	1
***Melinda gentilis***	1	0	0	?	0	?	?	1	***Metoplisa carbonaria***								?	?	?	?	?	?	?	?
***Lucilia sericata***	0	0	0	?	0	0	–	–	***Neotarsina andina***								?	?	?	?	?	?	?	?
***Cuterebra austeni***	?	?	?	?	?	?	?	?	***Neotarsina caraibica***								?	?	?	?	?	?	?	?
***Pollenia paupera***	0	0	0	?	0	?	?	–	***Oplisa tergestina***								0	1	0	0	0	1	0	0
***Eurychaeta muscaria***	?	?	?	?	?	?	?	?	***Parazamimus congolensis***								?	?	?	?	?	?	?	?
***Rhyncomya impavida***	?	?	?	?	?	?	?	?	***Paykullia insularis***								1	0	0	1	1	0	0	1
***Macquartia tenebricosa***	?	?	?	?	?	?	?	?	***Paykullia maculata***								1	0	0	1	1	0	0	1
***Acompomintho lobata***	?	?	?	?	?	?	?	?	***Phyto adolescens***								1	0	0	1	1	0	0	1
***Apomorphyto inbio***	?	?	?	?	?	?	?	?	***Phyto angustifrons***								1	0	0	1	1	0	0	1
***Aporeomyia* sp.**	?	?	?	?	?	?	?	?	***Queximyia flavipes***								?	?	?	?	?	?	?	?
***Axinia disjuncta***	?	?	?	?	?	?	?	?	***Rhinodonia antiqua***								?	?	?	?	?	?	?	?
***Axinia lucaris***	?	?	?	?	?	?	?	1	***Rhinodonia flavicera***								?	?	?	?	?	?	?	?
***Axinia miranda***	?	?	?	?	?	?	?	?	***Rhinomorinia sarcophagina***								0	1	0	0	0	1	0	0
***Azaisia* sp.**	?	?	?	?	?	?	?	?	***Rhinopeza gracilis***								?	?	?	?	?	?	?	?
***Baniassa fascipennis***	?	?	?	?	?	?	?	?	***Rhinophora lepida***								0	1	0	0	0	1	0	0
***Bezzimyia barbarista***	?	?	?	?	?	?	?	?	***Shannoniella cuspidata***								?	?	?	?	?	?	?	?
***Bezzimyia bisecta***	?	?	?	?	?	?	?	?	***Shannoniella setinervis***								?	?	?	?	?	?	?	?
***Bezzimyia busckii***	?	?	?	?	?	?	?	?	***Stevenia deceptoria***								0	1	0	0	0	1	0	0
***Bezzimyia hansoni***	?	?	?	?	?	?	?	?	***Stevenia palermitana***								0	1	0	0	0	1	0	0
***Bezzimyia yepezi***	0	1	1	0	0	1	1	0	***Tricogena rubricosa***								0	1	0	0	0	1	0	0
***Bixinia collessi***	?	?	?	?	?	?	?	?	***Tromodesia angustifrons***								?	?	?	?	?	?	?	?
***Bixinia winckleri***	?	?	?	?	?	?	?	?	***Trypetidomima fusca***								?	?	?	?	?	?	?	?
***Comoromyia* sp. 1**	?	?	?	?	?	?	?	?	***Trypetidomima lutea***								?	?	?	?	?	?	?	?
***Comoromyia* sp. 2**	?	?	?	?	?	?	?	?	***Ventrops aethiopicus***								?	?	?	?	?	?	?	?
***Kinabalumyia pinax***	?	?	?	?	?	?	?	?	***Ventrops freidbergi***								?	?	?	?	?	?	?	?
***Macrotarsina longimana***	?	?	?	?	?	?	?	?	***Ventrops hannemariae***								?	?	?	?	?	?	?	?
***Malayia fuscinervis***	?	?	?	?	?	?	?	?	***Ventrops incisus***								?	?	?	?	?	?	?	?
***Marshallicona quitus***	?	?	?	?	?	?	?	?	***Ventrops intermedius***								?	?	?	?	?	?	?	?
***Maurhinophora indoceanica***	?	?	?	?	?	?	?	?	***Ventrops milichioides***								?	?	?	?	?	?	?	?
***Melanomyiodes capensis***	?	?	?	?	?	?	?	?	***Ventrops stuckenbergi***								?	?	?	?	?	?	?	?

### Catalogue

Format

The present catalogue lists all nominal genera and species of Rhinophoridae, providing details about name-bearing types and with known distributions updated from both recent literature and our own identifications of museum specimens.

Valid taxa are arranged hierarchically and alphabetically, according to genus and species (subfamilial and tribal classification is considered premature given the difficulties in interpreting adult homologies and defining monophyletic groupings). Synonyms, including unjustified emendations and incorrect original and subsequent spellings, are listed chronologically for all names.

Each genus-group name is listed with the following formatting and information: genus name (in square brackets if unavailable, italics if available, bold + italics if valid), author, year, page, type species with author and date, form of type fixation with author and date. Each type species is given in its original binomen (Recommendation 67B of the “International Code of Zoological Nomenclature”, henceforth “the Code”, ICZN 1999), followed by its valid name, if different, in square brackets. Incorrect original spellings are given *teste* their First Reviser. Incorrect subsequent spellings encountered during this study are cited from their earliest occurrence.

Species are listed alphabetically by valid name followed by synonyms, nomina nuda, unjustified emendations and incorrect spellings listed chronologically. The genus *Bezzimyia* Townsend is likely polyphyletic and the species have been grouped into two species groups (namely Group A and Group B) and listed alphabetically within each group. The valid specific epithet is given in bold and italics followed by author and year. Each available name is given in italics in its original combination and spelling followed by author, year (with letter if applicable, to match with References), and page. Given next is the type locality in modern spelling, followed by information about the name-bearing type, consisting of status (holotype, lectotype, neotype or syntypes), sex, and acronym of type repository. Additional information may be given under “Remarks”. Distribution is given hierarchically and alphabetically according to biogeographical region and by country, but with larger countries separated into states/provinces and offshore islands listed separately from the mainland. Archipelagos may be listed by island when data are available. European distribution follows Fauna Europaea (https://fauna-eu.org/, see Pape et al. 2015).

Type localities are cited from largest to smallest geographic area or place. Country and state/province names are given only in their modern equivalents. Coordinates given in an original publication are cited as an integral part of the type locality, in their original format.

For data on the number and sex of name-bearing types other than an unambiguous fixation of a holo-, lecto- or neotype, we follow the format proposed by O’Hara and Cerretti (2016), slightly modified as explained below.

Type(s), male: One or more males. This citation is used for a species described from the male sex without indication whether the type series comprised a single male (i.e., a holotype) or more than one male (i.e., syntypes).

Type(s), female: One or more females.

Type(s), unspecified sex: One or more specimens with no indication of sex.

Syntypes, [number] male[s] and [number] female[s] (e.g., “3 males and 2 females”): Species described from both sexes, with the exact number of males and females specified and without a designated holotype.

Syntypes, males and females: Species described from both sexes, with more than one specimen of each sex but without specified numbers and without a designated holotype.

Syntypes, male(s) and female(s): Species described from both sexes, with no indication of the number of specimens of either sex, neither the exact number nor whether only one or more than one.

Syntypes, males: Species described from more than one male, without indication of the specific number of males and without a designated holotype.

Syntypes, females: Species described from more than one female, without indication of the specific number of females and without a designated holotype.

Syntypes, unspecified number and sex: Species described from more than one specimen but without indication of sex or number of specimens and without a designated holotype.

Avoidance of assumption of holotype and lectotypifications

Recommendation 73F of the Code (ICZN 1999), “Avoidance of assumption of holotype”, recommends, “where appropriate” and when it is possible that the nominal species-group taxon was based on more than one specimen, to designate a lectotype rather than assume a holotype; and Article 74.6 of the Code deems that an assumed monotypy where the original description neither implies nor requires that there were syntypes, is deemed to be a lectotype designation if it is considered subsequently that the original description was based on more than one specimen. We follow O’Hara et al. (2009) and O’Hara and Cerretti (2016) in using the term “lectotype designation” for an explicit lectotypification where the author used either the term “lectotype” or an exact translation or equivalent expression (e.g., “the type”), and the term “lectotype fixation” for an implicit lectotypification by inference of holotype as well as for cases where the original work reveals that the taxon had been based on more than one specimen none of which were designated as holotype and an author subsequently used the term “holotype” in a way that explicitly indicated that he or she was selecting from the type series that particular specimen to serve as the name-bearing type.

Distributional data

Distributions are cited for each valid species based on published records, examination of specimens in collections, and material collected by the authors or made available by colleagues. New country records are followed by “[**new record**]”, and the label data of the relevant specimen(s) upon which the new record is based are given in Table 4. The primary sources for distributions were Cerretti (2002, 2003, 2007), Colless (1994), Crosskey (1977), Draber-Mońko (2007), Ebejer (2011), Ginn (2012), Guimarães (1971), Herting (1961, 1993), Kugler (1978), Martínez and Nihei (2018), Mulieri et al. (2010), O’Hara et al. (2015), Pape (1998a, 1998b, 2010), Peris (1963), Rognes and Hansen (1996), Sabrosky and Arnaud (1965), Verves (2005, 2010, 2012), Verves and Khrokalo (2006, 2010), Verves et al. (2019), Wood (1987), and Zeegers (2008, 2011).

**Table 4.** New country records.

Species	Locality	Source / repository
*Acompomintho sinensis*	Tajikistan, Gorno-Badachšan, Rŭshan	NHMD
*Macrotarsina longimana*	Italy, Sicily, Palermo province, Bisacquino, Riserva Naturale Monte Genuardo 900 m, 12.VIII.2000, P. Cerretti leg.	MZUR
*Melanophora roralis*	Canada, Ontario	http://bugguide.net/node/view/443145
	USA, Ohio	http://www.inaturalist.org/observations/101858
	British Virgin Islands, St Thomas, Charlotte Amalie	AMNH
*Oplisa aterrima*	Italy, Sicily, Palermo province, Bosco della Ficuzza 600–1000 m, Torretta Torre, 18.V.2004, P. Cerretti leg.	MZUR
	Portugal, Coimbra, Buçaco Forest, 5.vii.1990, V. Michelsen (NHMD)	NHMD
*Paykullia braueri*	Croatia, Ličko-senjaska Co., 4 km NW Rudelić Draga, 280m, 44°28'41.92"N, 15°8'57.34"E, 14.vi.2012, E. Buenaventura, T. Pape, D. Whitmore	NHMD
*Paykullia maculata*	Spain, Granada, Trevélez (S Mulhacén), 1480m, 9.vii.1993, V. Michelsen	NHMD
*Phyto abbreviata*	Tunisia, 25 km E Gafsa, 11–13.iii.1986, Zool. Mus. Copenhagen Exp.	NHMD
*Phyto adolescens*	Greece, Pelopónnisos, Taïyetos Mts, 950–1800 m, 15–19.v.1990, Zool. Mus. Copenhagen Exp.	NHMD
*Phyto discrepans*	Malta, Buskett Garden, Rabat, 4–11.vi.1988, Stig Andersen	NHMD
	Morocco, 600 m, Checheouèn, 22.iv.1989, Zool. Mus. Copenhagen Exp.	NHMD
	Morocco, 300 m, Ouezzane, 21–22.iv.1989, Zool. Mus. Copenhagen Exp.	NHMD
	Morocco, 1150 m, 40 km N Fès, 20.iv.1989, Zool. Mus. Copenhagen Exp.	NHMD
	Portugal, Coimbra, Buçaco Forest, 5.vii.1990, V. Michelsen	NHMD
	Tunisia, 25 km SE Ain Draham, 10–16.v.1988, Zool. Mus. Copenhagen Exp.	NHMD
	Tunisia, Tabarka area, 7–18.v.1998, Zool. Mus. Copenhagen Exp.	NHMD
	Tunisia, 40 km, W Jendouba 17.v.1988, Zool. Mus. Copenhagen Exp.	NHMD
*Phyto discrepans*	Tunisia, El Kef area, 14.v.1988, Zool. Mus. Copenhagen Exp.	NHMD
	Tunisia, 15 km NW Kebili, 17.iii.1986, Zool. Mus. Copenhagen Exp.	NHMD
*Phyto melanocephala*	Croatia, Ličko-senjaska Co., 2.8 km SSE Sveti Juraj, 380m, 44°54'28.38"N, 14°56'26.84"E, 12.vi.2012, E. Buenaventura, T. Pape, D. Whitmore	NHMD
	Croatia, Ličko-senjaska Co., nr. Sušanj Cesarički, 850 m, 44°31'51.39"N, 15°7'37.46"E, 13.vi.2012, E. Buenaventura, T. Pape, D. Whitmore	NHMD
	Greece, Makedonia/Tessalia, Olympos 700–2100 m, 21–26.v.1990, Zool. Mus. Copenhagen Exp.	NHMD
	Sweden, Möckelmossen, Vickelby, 1.vi.2006, V. Michelsen	NHMD
	Turkey, Pamphylia, W of Alanya, 2–13.vi.1991, B. Petersen leg.	NHMD
*Rhinophora lepida*	Italy, Trentino-Alto Adige, Trento prov., Strada da Sdruzzinà a Villaggio san Michele, 810 m, 45°43'31"N, 10°58'01"W, 26–28.VIII.2015, D. Corcos	MZUR
	Italy, Veneto, Belluno prov., Misurina, 1400 m, 46°32'54.08"N, 12°14'51.08"W, 30.VIII–1.IX.2015, D. Corcos	MZUR
	Italy, Veneto, Belluno prov., Misurina, 1500 m, 46°33'21.26"N, 12°14'48.26"W, 30.VII–1.VIII.2015, D. Corcos	MZUR
	Italy, Veneto, Belluno prov., Misurina, 1600 m, 46°33'43.96"N, 12°14'41.61"W, 30.VII–1.VIII.2015, D. Corcos	MZUR
	Italy, Veneto, Belluno prov., Cortina-Passo Falzarego, 1300 m, 46°32'7.30"N, 12°7'25.25"W, 30.VII–1.VIII.2015, D. Corcos	MZUR
	Italy, Veneto, Belluno prov., Cortina-Passo Falzarego, 2100 m, 46°31'8.67"N, 12°0'40.55"W, 30.VII–1.VIII.2015, D. Corcos	MZUR
	Italy, Veneto, Belluno prov., Cortina-Passo Giau, 1800 m, 46°29'51.45"N, 12°4'33.87"W, 30.VII–1.VIII.2015, D. Corcos	MZUR
	Italy, Veneto, Belluno prov., Cortina-Passo Giau, 1900 m, 46°29'32.44"N, 12°4'20.13"W, 30.VII–1.VIII.2015, D. Corcos	MZUR
*Stevenia atramentaria*	Greece, Pelopónnisos, Taïyetos Mts, 950–1800 m, 15–19.v.1990, Zool. Mus. Copenhagen Exp.	NHMD
*Stevenia bertei*	Croatia, Ličko-senjaska Co., nr. Sušanj Cesarički, 850 m, 44°31'51.39"N, 15°7'37.46"E, 13.vi.2012, E. Buenaventura, T. Pape, D. Whitmore	NHMD
	Croatia, Ličko-senjaska Co., nr. Baške Oštarije, 920m, 44°31'34.50"N, 15°10'5.50"E, 13.vi.2012, E. Buenaventura, T. Pape, D. Whitmore	NHMD
	Croatia, Ličko-senjaska Co., nr. Podoštra, 665m, 44°31'48.63"N, 15°19'35.92"E, 15.vi.2012, E. Buenaventura, T. Pape, D. Whitmore	NHMD
*Stevenia hirtigena*	Israel, Negev, Ein Avdat NP, 25.v.2004, K. Szpila	NHMD
*Stevenia signata*	Turkey, Pamphylia, W of Alanya, 2–13.vi.1991, B. Petersen leg.	NHMD
*Stevenia umbratica*	Tunisia, Ain Drahanm area, 5–18.v.1988, Zool. Mus. Copenhagen Exp.	NHMD
*Tromodesia setiventris*	Pakistan, Normal (nr. Skardu), NWFP. 1988.8.16, T. Hayashi	NHMD

### Collections

**AMNH**American Museum of Natural History, New York, USA.

**ANIC**Australian National Insect Collection, CSIRO, Canberra, Australia.

**NBCL**Naturalis Biodiversity Center, Leiden (including collections formerly deposited at Zoölogisch Museum, Universiteit van Amsterdam), Netherlands.

**BLKU**Biosystematics Laboratory, Kyushu University, Fukuoka, Japan.

**BPBM**Bernice Pauahi Bishop Museum, Honolulu, Hawaii, USA.

**CNC**Canadian National Collection of Insects, Agriculture and Agri-Food Canada, Ottawa, Canada.

**CULSP** Czech University of Life Sciences, Prague, Czech Republic.

**DEI**Deutsches Entomologisches Institut, Leibniz-Zentrum für Agrarlandschaftsforschung, Müncheberg, Germany.

**ENSAM** École nationale supérieure agronomique de Montpellier, France.

**INBio**Instituto Nacional de Biodiversidad, San José, Costa Rica [INBio collections are now under the care of the Museu Nacional de Costa Rica, San José].

**IRSNB**Institut Royal des Sciences Naturelles de Belgique, Brussels, Belgium.

**LSUK** Linnean Society, London, United Kingdom.

**MIZA**Museo del Instituto de Zoología Agrícola, Maracay, Venezuela.

**MNHN**Muséum national d’Histoire naturelle, Paris, France.

**MNCN**Museo Nacional de Ciencias Naturales, Madrid, Spain.

**MRAC**Musée Royal de l’Afrique Centrale.

**MZF** Museo Zoologico “La Specola”, Florence, Italy.

**MZLU**Museum of Zoology, Lund University, Lund, Sweden.

**MZSP**Museu de Zoologia da Universidade de São Paulo, São Paulo, Brazil.

**MZUR**Museo di Zoologia. Sapienza Università di Roma, Rome, Italy.

**NHMD**Natural History Museum of Denmark, University of Copenhagen, Copenhagen, Denmark. [Formerly as ZMUC.]

**NHMUK**Natural History Museum [formerly British Museum (Natural History)], London, United Kingdom.

**NHMW**Naturhistorisches Museum Wien, Vienna, Austria.

**NHRS**Naturhistoriska riksmuseet, Stockholm, Sweden.

**NMBA**Naturhistorisches Museum der Benediktiner-Abtei Admont, Admont, Austria.

**NMDA**Department of Arthropoda, KwaZulu-Natal Museum, Pietermaritzburg, South Africa.

**NMNW**National Museum of Namibia, Windhoek, Namibia.

**PUCE** Pontificia Universidad Cátolica Ecuador, Quito, Ecuador.

**QDPC**Queensland Department of Primary Industries, Indooroopilly, Queensland, Australia.

**SAMC**Iziko South African Museum, Cape Town, South Africa.

**SMNS**Staatliches Museum für Naturkunde Stuttgart, Stuttgart, Germany.

**TAU**The Steinhardt Museum of Natural History, Tel Aviv University, Tel Aviv, Israel.

**TFMC**Museo de Ciencias Naturales de Santa Cruz de Tenerife, Canary Islands, Spain.

**UASK**National Academy of Sciences of Ukraine, Kiev, Ukraine.

**USNM** National Museum of Natural History [formerly United States National Museum], Smithsonian Institution, Washington DC, USA.

**ZIN**Zoological Institute, Russian Academy of Sciences, St. Petersburg, Russia.

**ZMHB** Museum of Natural History, Leibniz-Institute for Research on Evolution and Biodiversity, Berlin, Germany.

**ZMUM** Zoological Museum of Moscow University, Moscow, Russia.

## Key to world genera of Rhinophoridae

The following is a standard dichotomous key with couplets containing the main characters chosen for the key path, with alternative states in the respective entries of the couplet, listed by their supposed diagnostic strength. Occasionally, supplementary information is given in square brackets where considered helpful to secure an identification. The key includes the genus *Alvamaja* as well as the Afrotropical *Morinia* “*carinata* species group” (Pape 1997, Rognes 2010), all of which were recently transferred from Rhinophoridae to the re-established oestroid family Polleniidae (Cerretti et al. 2019). As these polleniids are nearly indistinguishable from rhinophorids, and given the lack of comprehensive keys to world oestroid families and genera, their inclusion is deemed helpful. They are given within square brackets. The key also includes the genus *Aporeomyia*, which was originally tentatively assigned to the Tachinidae based on the morphology of the phallus, but which is here moved to Rhinophoridae based on a re-assessment of the homology of relevant phallic sclerotisations (see below).

**Table:** 

1	Body ground colour metallic green or blue-violet. Postalar wall with a tuft of fine setulae	[***Alvamaja* Rognes; Polleniidae**]
–	Body ground colour not metallic green or blue-violet, at most shiny black. Postalar wall bare [if with short setae then not Rhinophoridae, see couplets 8, 13, 17, 22]	**2**
2	Brachypterous, micropterous or apterous specimen (Fig. 4H), female only	**3**
–	Wing fully developed, male or female	**4**
3	Tergite 7 and sternite 7 forming a sclerotised, dorsoventrally flattened oviscapt (Fig. 13K–N, or similar structures)	***Axinia* Colless (in part)**
–	Segment 7 normally developed, i.e., not modified into a dorsoventrally flattened oviscapt	***Bezzimyia* Townsend (in part)**
4	Vein M_1_ running nearly straight or evenly curved forward to wing margin (Figs 9B, C; 10D, M, N; 16I; 17J; 18F), i.e., without a distinct bend	**5**
–	Vein M_1_ with a distinct, sometimes shallow bend that can be well removed from wing margin (e.g., Figs 9A, D–F, K, L; 10B, C, E–L) or very close to it (Fig. 9G), or M_1_ not reaching wing margin (i.e., ending freely in the wing membrane) (Fig. 9H–J)	**11**
5	Shiny black or brown flies, usually with bright yellow antenna or, more rarely, with variously patterned legs; with very short setae on head, scutum and abdomen, of approx. the same size as the smallest clothing setae (Fig. 4C–E). Vein CuA+CuP not reaching wing margin. Male: postpedicel characteristically rounded-subtriangular (axe-head-like and shorter than maximum distal width) (Fig. 6C–F), the thickened posterior margin abutting the enlarged, sunken face or anterior margin forming two or more lobes; arista very short (i.e., distinctly shorter than length of postpedicel) to nearly absent, and arising apically or sub-apically on postpedicel (Fig. 6C–F). Female: tergite 7 and sternite 7 forming a strongly sclerotised, dorsoventrally flattened and evenly curved, usually long oviscapt (Fig. 13K–N)	***Axinia* Colless**
–	Body colour varies from black to yellow, microtomentum present or absent; setae of head and thorax usually normally developed. Male: postpedicel usually not triangular in lateral view (if so, then postpedicel much longer than its maximum distal width and vein CuA+CuP reaching wing margin), arista arising in proximal half (Fig. 18C, E). Female: oviscapt not modified	6
6	Antenna short, at most as long as minimum height of gena (usually much shorter) (Figs 16D, F; 17F, H). Antenna arising distinctly below middle of compound eye. Tarsus of fore leg strongly laterally compressed (Figs 16A, H; 17B, E). Palpus strongly reduced, approx. 1–2 times as long as wide	***Neotarsina* Cerretti & Pape, gen. nov.**
–	Antenna much longer than minimum height of gena, arising at or above middle of compound eye. Tarsus of fore leg not laterally compressed. Palpus usually well developed, if reduced then mouthparts vestigial	**7**
7	First postsutural supra-alar seta well developed, at least as long and robust as posterior notopleural seta (Fig. 13B, F)	**8**
–	First postsutural supra-alar seta absent or minute (Fig. 13E)	**9**
8	Head longer than high and facial profile not receding. Prementum at least as long as head height and labella narrow. Antenna brown to black. Upper half of facial plate carinate (Fig. 13A). Postalar wall usually with at least one small setula. Male: cerci normally developed and medially not fused into a syncercus; surstylus articulated (i.e., not fused) to epandrium; extension of dorsal sclerite of distiphallus divided medially into two sclerites; median process of ventral sclerotisation of distiphallus not fused to base of ventral sclerotisation	[***Morinia* “ *carinata* species group” (in part); Polleniidae**]
–	Head higher than long (even if slightly so) and facial profile receding (Figs 5E; 7H). Prementum usually shorter than height of head, if approx. as long as head height and labella narrow, then antenna pale yellow. Face not carinate. Postalar wall bare. Male: cerci very short and medially fused into a syncercus; surstylus fused to epandrium; extension of dorsal sclerite of distiphallus not divided medially; median process of ventral sclerotisation of distiphallus very long, narrow and fused to base of ventral sclerotisation	***Rhinodonia* Cerretti, Lo Giudice & Pape**
9	Vein CuA+CuP not reaching wing margin (Fig. 10M). First aristomere at most as long as wide, second aristomere approx. 4 times as long as wide. Mouthparts strongly reduced (vestigial). Male: proclinate orbital setae absent; postpedicel normal, i.e., not divided longitudinally into lobes; cerci not fused medially, distally well divided into two pointed branches; surstylus fused to epandrium	***Rhinopeza* Cerretti, Lo Giudice & Pape**
–	Vein CuA+CuP reaching wing margin (Fig. 9B). First aristomere at least 4 times as long as wide, second aristomere at least 8 times as long as wide (at least half as long as third aristomere). Mouthparts normally developed or strongly reduced. Male: 0–1 proclinate orbital setae; postpedicel varying from normal to branching into three lobes; cerci medially fused into a syncercus or distally well-divided into two pointed branches; surstylus not fused to epandrium	**10**
10	Mouthparts normally developed (Fig. 18A, C, E). Male: 1 proclinate orbital seta; postpedicel whole, i.e., not divided longitudinally into lobes (Figs 18A, C, E); median distal margin of first and second metatarsomeres without modified setae; cerci medially fused into a syncercus (Fig. 18G)	***Kinabalumyia* Cerretti & Pape, gen. nov.**
–	Mouthparts strongly reduced (proboscis very short, though palpus well developed). Male: proclinate orbital setae absent; postpedicel divided longitudinally into three lobes (Figs 4B; 6B); median distal margin of first and second metatarsomeres provided with a comb of flattened setae, which are distally jagged; cerci proximally fused, distally divided into two pointed branches (similar to the condition shown in Fig. 15F)	***Aporeomyia* Pape & Shima**
11	Vein M_1_ not reaching wing margin (i.e., ending freely in the wing membrane) (Fig. 9H–J)	**12**
–	Vein M_1_ reaching wing margin (Figs 9A, D, K, L; 10B, E, H–K) or fused to vein R_4+5_, so that cell r_4+5_ is petiolate (Figs 9E, F; 10C, F, G, L)	**15**
12	Male and female without proclinate orbital setae. Male: surstylus fused to epandrium	***Bezzimyia* Townsend (in part)**
–	Male and female with at least one proclinate orbital seta. Male: surstylus either freely articulating with or fused to epandrium	**13**
13	Prementum long and slender, at least as long as head height, and labella narrow. Postalar wall usually with at least one small setula. Postpronotum with two setae and facial profile not receding	[***Morinia lactineala* (Pape), *Morinia* “ *carinata* species group” (in part): Polleniidae**]
–	Prementum shorter than head height, and labella fleshy and normally developed. Postalar wall bare. Facial profile receding or not; if not receding then postpronotum with three setae arranged in a right-angled triangle	**14**
14	Facial profile not receding, i.e., vibrissal angle distinctly in front of anterior margin of eye (Fig. 7E). Postpronotum with three setae arranged in a right-angled triangle (or nearly so) (as in Fig. 2C). Male: one proclinate orbital seta. Female: 2–3 proclinate orbital setae	***Oplisa* Rondani (in part)**
–	Facial profile receding, i.e., vibrissal angle approx. in line with or behind anterior margin of eye (head with postcranial surface oriented vertically) (Figs 7B, 8B–E). Postpronotum with 1–3 setae, if three then arranged in a line or an obtuse triangle (as in Fig. 2B). Male: at least two proclinate orbital setae. Female: 3–5 proclinate orbital setae	**24**
15	Posterior lappet of metathoracic spiracle distinctly larger than anterior lappet (Fig. 13H). Female: thorax yellow, brown or black	**16**
–	Anterior and posterior lappets of metathoracic spiracle approx. of equal size and standing out from spiracular rim (i.e., almost perpendicular to pleural surface) (Fig. 13I), sometimes lappets not differentiated at all (Fig. 13J). Female: thorax usually black or brown, rarely yellow	**18**
16	Vein R_1_ setose along full length dorsally. Vein R_4+5_ with setulae dorsally extending from base to approx. level of bend of vein M_1_. Cell r_4+5_ open (Fig. 14A, C). Parafacial bare. Facial ridge with a row of setae on lower 2/3 (Fig. 14A, D). Vibrissa well developed. Palpus absent. Female: head, thorax, legs and abdomen mostly pale yellow (Fig. 14). Male unknown	***Maurhinophora* Cerretti & Pape, gen. nov.**
–	Vein R_1_ bare. Vein R_4+5_ with a few short setulae confined at base or bare. Cell r_4+5_ open or petiolate. Parafacial setulose at least on upper half (Figs 6J; 13C, D). Facial ridge bare or with a few decumbent setae on lower third. Palpus well developed	**17**
17	Vein R_4+5_ with a few short setulae confined to base. Cell r_4+5_ petiolate (Fig. 9E, F). Anterior lappet of metathoracic spiracle without setae. Female: thorax yellow (Fig. 4G). Male: at least tip of wing membrane smoky (Fig. 9E); frons very narrow (i.e., head almost holoptic), without proclinate or lateroclinate orbital setae	***Baniassa* Kugler**
–	Vein R_4+5_ bare. Cell r_4+5_ open. Anterior lappet of metathoracic spiracle with setae. Female: thorax black or brown. Male: wing membrane evenly yellowish; frons broad with at least one proclinate or lateroclinate orbital seta (i.e., head non-holoptic)	[***Morinia* “ *carinata* species group” (in part): Polleniidae**]
18	First postsutural supra-alar seta present and well developed, as long as or longer than notopleural setae (Fig. 13B, F)	**19**
–	First postsutural supra-alar seta absent or very short, distinctly shorter and weaker than notopleural setae (Fig. 13E, G)	**24**
19	Palpus absent. Facial ridge with a row of setae on lower 2/3 (Fig. 14D). Vein R_1_ entirely setulose dorsally. Vein R_4+5_ with setulae dorsally, extending from base to approx. level of bend of vein M_1_. Head, thorax, legs and abdomen mainly yellow (Fig. 14)	***Maurhinophora* Cerretti & Pape, gen. nov.**
–	Palpus present and usually well developed. Facial ridge with at most a few setulae just above vibrissa. Vein R_1_ bare. Vein R_4+5_ dorsally with a few short setulae confined to base or with one strong setula. Body colour variable	**20**
20	Palpus, tibiae, femora, pleural sclerites of thorax and sides of abdomen yellow (Fig. 5D). Head distinctly higher than long in lateral view. Facial ridge nearly straight. Lunule bare. Abdominal tergites 3 and 4 with strong, erect median discal setae (Fig. 11G). Male tergite 6 not differentiated	***Queximyia* Crosskey**
–	Palpus, tibiae, femora, pleural sclerites of thorax and sides of abdomen black or blackish-brown. Head varying from longer than high to higher than long. Facial ridge usually concave. Lunule bare or with setulae. Abdominal discal setae, if present, not as above. Male tergite 6 always present	**21** ^1^
21	Lunule with setulae (Fig. 13C). Katepimeron with at least one seta anteriorly. Male with or without orbital setae (lateroclinate and/or reclinate)	***Phyto* Robineau-Desvoidy** ^2^
–	Lunule bare. Katepimeron bare. Male with orbital setae (lateroclinate and/or reclinate)	**22**
22	Upper half of facial plate carinate (Fig. 13A) (sometimes only slightly so). Base of vein R_4+5_ dorsally bare. Postalar wall usually with a tuft of short setulae (rarely bare). Anterior lappet of metathoracic spiracle usually with 1–7 setae. Prementum varying from normally developed to long and slender with narrow labella	[***Morinia* “ *carinata* species group” (in part): Polleniidae**]
–	Facial plate not carinate. Base of vein R_4+5_ dorsally with one long setula (Fig. 10B). Postalar wall bare. Anterior lappet of metathoracic spiracle without setae. Prementum distinctly shorter than head height and labella unmodified (i.e., broad)	**23**
23	Facial profile moderately receding, i.e., vibrissal angle in line with antennal insertion (postcranial surface oriented vertically) (as in Fig. 7D). Male with orbital setae (only one, strong)	***Comoromyia* Crosskey**
–	Facial profile not receding, i.e., vibrissal angle produced forwards and in front of antennal insertion (Fig. 7I). Male without proclinate orbital setae	***Rhinomorinia* Brauer & Bergenstamm** ^3^
24	Antenna distinctly shorter than height of gena (Figs 7B–D, F; 8A, B). Gena at least 0.5 times as high as compound eye; if less, then (i) mouthparts strongly reduced, vestigial and (ii) postangular section of vein M_1_ absent (Figs 4K; 9H–J). Male with proclinate orbital setae	**25**
–	Antenna at least as long as height of gena (usually distinctly longer), and gena at most 0.35 times as high as compound eye; if more, then mouthparts normally developed and vein M_1_ complete	**29**
25	Cell r_4+5_ open or vein M_1_ vanishing on wing membrane (i.e., postangular section of vein M_1_ absent)	**26**
–	Cell r_4+5_ long petiolate; petiole 0.8–2.5 times as long as postangular section of vein M_1_ (Figs 4L; 10L)	**32**
26	Postangular section of vein M_1_ absent and vein ending freely in wing membrane (Fig. 4K). Mouthparts strongly reduced, vestigial (Fig. 7B, C). Scutellum with only one pair of defined marginal setae (subapicals), diverging or subparallel. Thorax and abdomen characterised by a thick cover of silver microtomentum, which is brilliant in anterodorsal view (Figs 4K; 11A) (*M. argyriventris*, *M. basilewskyi*), or dull black (*M. asetosa*). Lunule bare. Male: trichia on arista bottle-brush like	***Melanophora* Meigen** ^4^
–	Vein M_1_ complete, i.e., reaching wing margin (Figs 9L; 10J; 15E). Mouthparts small, but normally developed. Scutellum usually with two pairs of well-developed marginal setae (basals and converging or crossed apicals). Microtomentum of thorax and abdomen not as described above. Lunule bare or with setulae. Male: trichia on arista usually not bottle-brush like (except *Marshallicona* Cerretti & Pape, gen. nov.)	**27**
27	Male and female without proclinate orbital setae (Fig. 8A). Wing infuscate with 3 whitish, hyaline spots (Figs 5J; 10J)	***Trypetidomima* Townsend**
–	Male and female with at least one proclinate (or lateroclinate) orbital seta (Figs 7F; 15C). Wing membrane not as described above	**28**
28	Vein R_1_ dorsally setose on distal 1/4. Vein CuA+CuP not reaching wing margin. Costal sector cs_5_ longer than costal sector cs_2_ (Fig. 15E). Thorax blackish brown (Fig. 15A). Katepimeron bare. Lunule bare. Male: arista bottle-brush like; 4–5 proclinate orbital setae (Fig. 15C, D)	***Marshallicona* Cerretti & Pape, gen. nov.** (live habitus Fig. 1C)
–	Vein R_1_ dorsally bare. Vein CuA+CuP reaching wing margin. Costal sector cs_5_ shorter than costal sector cs_2_ (Fig. 9L). Thorax mainly yellow (Fig. 5B). Katepimeron usually with a few setulae anteriorly. Lunule usually setulose. Male: arista bare; 1–2 proclinate orbital setae	***Parazamimus* Verbeke**
29	Vein R_1_ entirely setulose dorsally. Scutellum with only one pair of defined marginal setae (subapicals), diverging or subparallel. Head shape strongly modified especially in male, with sunken face and vibrissal angle conspicuously projected forward and turned inwards apically (Fig. 7L). [Wing infuscate with 3 or 4 whitish spots (as in Fig. 10J). Male: proclinate orbital setae present; first aristomere elongated, more than twice as long as wide.]	***Shannoniella* Townsend**
–	Vein R_1_ bare dorsally, if with 1–3 setulae distally, then scutellum with at least 3 marginal setae. Head shape not as described above	**30**
30	Facial profile receding, i.e., vibrissal angle approx. in line with or behind anterior margin of eye (head with postcranial surface oriented vertically) (Figs 6A, K, L; 7B–D, F, G; 8A, C)	**31**
–	Facial profile not receding, i.e., vibrissal angle distinctly in front of anterior margin of eye (Figs 6G; 7A, E, I, J, K)	**41**
31	Cell r_4+5_ long petiolate (Figs 4L; 5C, G, L; 10C, F, G, L)	**32**
–	Cell r_4+5_ open (Figs 9A, D, G, K, L; 10B, E, J, K), closed at wing margin (Fig. 10H, I) or vein M_1_ ending freely in membrane (Fig. 9J)	**34**
32	Parafacial with a row of long robust setae (Fig. 8E). Male: arista not bottle-brush like. Female: wing without whitish posterior subapical spot	***Ventrops* Crosskey (*V. stuckenbergi* Cerretti & Pape)**
–	Parafacial with fine setulae at most on upper half (Fig. 13D). Male: arista bare, plumose or bottlebrush-like. Female: whitish posterior subapical spot on wing membrane absent or present	**33**
33	Both sexes with 3–7 proclinate orbital setae. Male: trichia on arista bottlebrush-like (Figs 7B; 13D); posterior margin of sternite 5 with a deep median cleft. Female: wing membrane mainly brownish with whitish posterior subapical spot	*** Melanophora *^5^**
–	Male with 0–2, female with 1–3 proclinate orbital setae. Male arista bare to short pubescent, not bottlebrush-like; sternite 5 with almost straight posterior margin. Female: wing varying from hyaline to variously patterned but without whitish posterior subapical spot	***Paykullia* Robineau-Desvoidy**
34	Facial ridge with robust setae on lower 1/2 or more. Vein CuA+CuP reaching wing margin. Female: postpedicel with a row of setae medially or dorsally (Fig. 6L)	***Malayia* Malloch**
–	Facial ridge with only a few decumbent setulae reaching at most the lower 1/3. Vein CuA+CuP usually not reaching wing margin (except in *Bixinia winkleri* Cerretti, Lo Giudice & Pape from Australia). Female: postpedicel without setae	**35**
35	Parafacial bare. Lateral vertical seta not differentiated from postocular row (as in Fig. 8A)	**36**
–	Parafacial setose at least on upper half (Figs 6A; 8E). Lateral vertical seta well differentiated from postocular row (Fig. 8D, E); if not differentiated, then lunule with setulae	**38**
36	Bend of vein M_1_ distinctly rounded and very close to wing margin (Fig. 9G). Antenna distinctly longer than compound eye height, and facial ridge longer than frons (Fig. 6K)	***Bixinia* Cerretti, Lo Giudice & Pape**
–	Bend of vein M_1_ well removed from wing margin (Fig. 9K, L). Antenna shorter than compound eye height and facial ridge shorter than frons (Fig. 8A, C)	**37**
37	Male and female with at least 2 proclinate orbital setae (Fig. 8C). Wing infuscate mostly along veins, without whitish spots. Lower calypter without long, blackish setulae along margin. Posterior margin of eye indented in lateral view (Fig. 8C). Eye enormously developed so that gena and parafacial are practically obliterated. Male cerci very short	***Ventrops* Crosskey** ^6^
–	Male and female without proclinate orbital setae (Fig. 8A). Wing infuscate with 3 whitish, hyaline spots (Figs 10J). Lower calypter with long, blackish setulae along margin. Posterior eye margin not indented (Fig. 8A). Eye large but not enormously developed so that both gena and parafacial are distinct. Male cerci well developed	***Trypetidomima* Townsend**
38	Antennal insertion distinctly above eye middle. Antenna long, at least as long as eye height. Second aristomere elongated, 2–3 times as long as wide	***Acompomintho* Villeneuve**
–	Antennal insertion at or below eye middle (Figs 6A; 8D, E). Antenna distinctly shorter that eye height. Second aristomere at most as long as wide	**39**
39	Male and female without proclinate (or lateroclinate) orbital setae (Fig. 6A). Lunule setose. Legs largely red (Fig. 4A). Male: surstylus fused to epandrium	***Apomorphyto* Cerretti, Lo Giudice & Pape**
–	Male and female with proclinate orbital setae (Fig. 8D, E). Lunule bare. Legs black or blackish-brown. Male: surstylus not fused to epandrium	**40**
40	Abdominal tergites extensively covered with whitish-grey reflecting microtomentum except along posterior margins of tergites 3–5 and along a narrow longitudinal median vitta. Head and thorax extensively covered with grey reflecting microtomentum. Basicosta yellow. Male: arista pubescent, longest trichia distinctly longer than maximum, basal diameter or arista. Body length: 6 mm	***Ventrops* Crosskey (undescribed species from South Africa)**
–	Head, thorax and abdomen shiny black or nearly so, i.e., with microtomentum sparse or almost absent. Basicosta black or yellow. Male: arista apparently bare, i.e., trichia clearly shorter than its maximum diameter. Body length less than 4 mm	***Ventrops* Crosskey** ^7^
41	Distal section of CuA+CuP (i.e., distal to junction with CuA) approx. 1/2 of total length of CuA+CuP (Fig. 9C, D; 10A). Anterior katepisternal seta weak and at most 2/3 as long as posterior one	**42**
–	Distal section of CuA+CuP approx. 3/4 of total length of CuA+CuP (Figs 9A, E, G; 10E–G). Anterior katepisternal seta robust and more than 2/3 as long as posterodorsal katepisternal seta	**43**
42	Anterior katepisternal seta more than 1/2 as long as posterior seta. Second aristomere slightly elongated, approx. 1.5 times as long as wide (Fig. 6G, H). Male: fore tarsus not elongated or compressed (Fig. 4F); wing membrane hyaline or evenly smoked (Fig. 9D)	***Azaisia* Villeneuve**
–	Anterior katepisternal seta distinctly less than 1/2 as long as posterior seta. Second aristomere at most as long as wide (Fig. 7A). Male: fore tarsus greatly elongated and laterally compressed (Fig. 4J); wing membrane darkened around distal third of vein R_2+3_ (Fig. 4J)	***Macrotarsina* Schiner**
43	Setae present on lunule (Fig. 13C), notopleuron and katepimeron simultaneously. Median process of ventral sclerotisation of distiphallus interrupted proximally and not connected to ventral plate (Fig. 12G)	***Phyto* Robineau-Desvoidy (*P. pauciseta* Herting)**
–	Setae never present on lunule, notopleuron and katepimeron simultaneously. Median process of ventral sclerotisation of distiphallus not interrupted, running from ventral plate to tip of phallus	**44**
44	Postpronotum with three setae arranged in right-angled triangle or nearly so; if with two setae (one species of *Stevenia*), then parafacial with strong setae in lower half	**45**
–	Postpronotum with three setae arranged in a line or in a shallow triangle; if with only two setae, then parafacial bare	**47**
45	Parafacial bare or with hair-like setulae in upper half; if parafacial setae continue on lower half, these are never robust (Fig. 7E). Cell r_4+5_ never petiolate	***Oplisa* Rondani (in part)**
–	Parafacial with a row of strong bristly setae in lower half (Fig. 7J, K). Cell r_4+5_ narrowly open, closed at wing margin or petiolate (Fig. 10H, I, L)	**46**
46	Cell r_4+5_ narrowly open, closed at wing margin or, more rarely, short-petiolate (petiole, when present, shorter than crossvein r-m). Mid tibia with 1 anterodorsal seta. Male habitus in lateral view as in Fig. 5H	***Tricogen* a Rondani**
–	Cell r_4+5_ distinctly petiolate (Fig. 10L) (petiole approx. as long as crossvein r-m in *S. acutangula*). Mid tibia with at least 2 anterodorsal setae. Male habitus in lateral view as in Fig. 5G	***Stevenia* Robineau-Desvoidy**
47	Arista plumose, i.e., longest trichia approx. 2.5 times as long as maximum diameter of arista (Fig. 8B)	***Tromodesia* Rondani**
–	Arista bare or with trichia at most 1.5 times as long as maximum diameter of arista	**48**
48	Base of vein R_4+5_ bare or with a few, short, fine setulae, distinctly shorter than crossvein r-m. Vibrissal angle more or less in line with antennal insertion (postcranial surface oriented vertically) (Figs 6G; 7E)	**49**
–	Base of vein R_4+5_ with at least one strong setula, longer than crossvein r-m, with or without additional shorter setulae. Vibrissal angle produced forwards and in front of antennal insertion (Figs 7I; 12C)	**51**
49	Cell r_4+5_ open or closed at wing margin, very rarely petiolate but petiole at most approx. half as long as crossvein r-m	**50**
–	Cell r_4+5_ with a petiole 1.2–2.5 times as long as crossvein r-m	**52**
50	Frontal vitta, palpus (Figs 6G, H; 7A) and basicosta yellow	**42**
–	Frontal vitta, palpus and basicosta black	***Metoplisa* Kugler**
51	Cell r_4+5_ open or closed at wing margin, rarely petiolate with petiole approx. half as long as crossvein r-m. Scutum with two presutural microtomentose vittae. Palpus at least as long as postpedicel (Fig. 7I). Male: head not holoptic, with upper reclinate orbital setae usually differentiated (except *R. capensis*)]	***Rhinomorinia* Brauer & Bergenstamm (in part)**
–	Cell r_4+5_ with a petiole 1.2–2.5 times as long as crossvein r-m. Scutum dark with no presutural microtomentose vittae differentiated. Palpus varying from shorter to longer than postpedicel. Male: head holoptic or not so, with or without upper reclinate orbital setae	**52**
52	Parafacial entirely bare. Palpus shorter than postpedicel. Lunule bare. Male: head almost holoptic, upper reclinate orbital setae not differentiated (Fig. 12A, C). Male terminalia as in Fig. 12D, E	***Melanomyoides* Crosskey**
–	Parafacial with a row or short setulae. Palpus longer than postpedicel. Lunule with setulae. Male: head not holoptic, with upper reclinate orbital setae usually differentiated	***Rhinophora* Robineau-Desvoidy**

**Figure 4.**
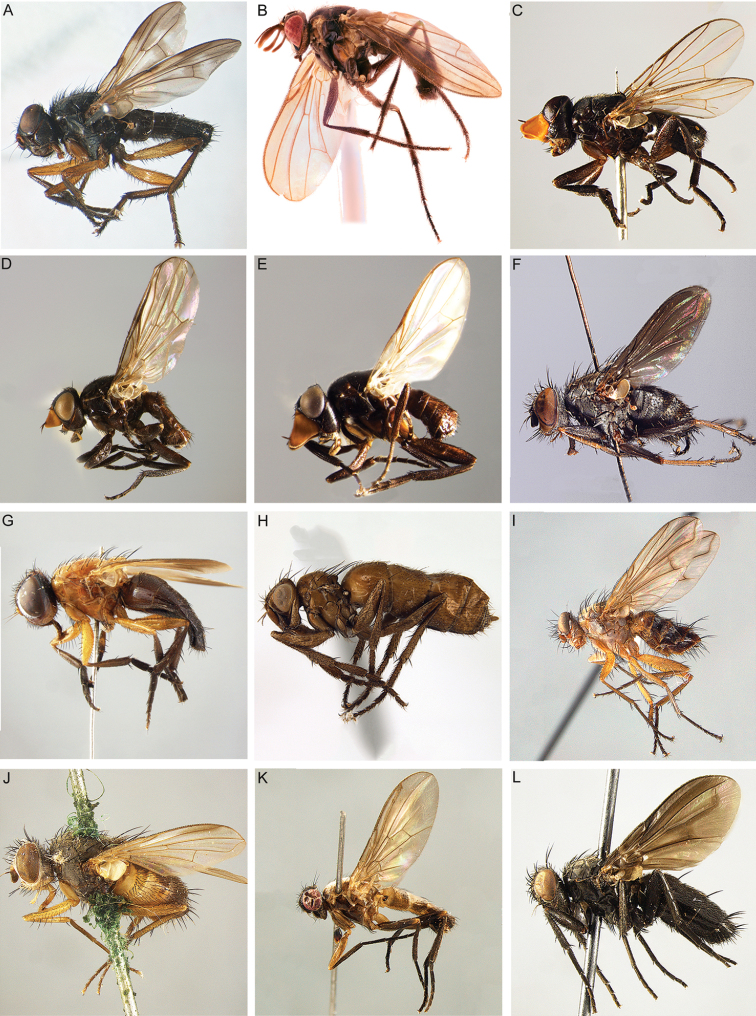
Rhinophoridae, habitus in lateral view. **A***Apomorphyto inbio* ♂ (Costa Rica) [holotype] **B***Aporeomyia* sp. ♂ (Malaysia, Sabah) **C***Axinia zantae* ♂ (Australia) **D***Axinia arenaria* ♂ (Australia) **E***Axinia brevispica* ♂ (Australia) **F***Azaisia* sp. ♂ (Portugal, Azores) **G***Baniassa fascipennis* ♀ (Israel) [paratype] **H***Bezzimyia hansoni* ♀ (Costa Rica) **I***Comoromyia* sp. ♀ (Madagascar) **J***Macrotarsina longimana* ♂ (Italy) **K***Melanophora basilewskyi* ♂ (Kenya) **L***Melanophora roralis* ♂ (Italy).

**Figure 5.**
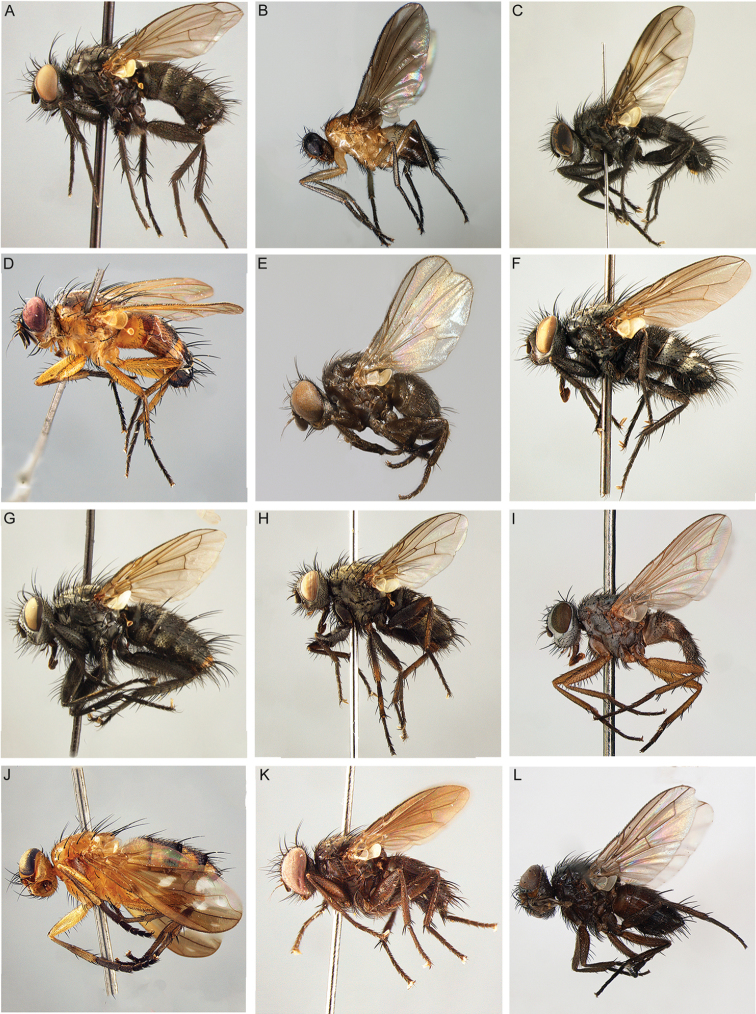
Rhinophoridae, habitus in lateral view. **A***Oplisa tergestina* ♂ (Italy) **B***Parazimimus congolensis* ♂ (Burundi) **C***Paykullia partenopea* ♂ (Italy) **D***Queximyia flavipes* ♂ (South Africa) **E***Rhinodonia antiqua* ♂ (New Caledonia) [holotype] **F***Rhinomorinia sarcophagina* ♂ (Italy) **G***Stevenia palermitana* ♂ (Italy) [holotype] **H***Tricogena rubricosa* ♂ (Morocco) **I***Tromodesia angustifrons* ♀ (Israel) [paratype] **J***Trypetidomima lutea* ♀ (Brazil) **K***Ventrops incisus* ♂ (Tanzania) [paratype] **L***Ventrops stuckenbergi* ♂ (Namibia) [holotype].

**Figure 6.**
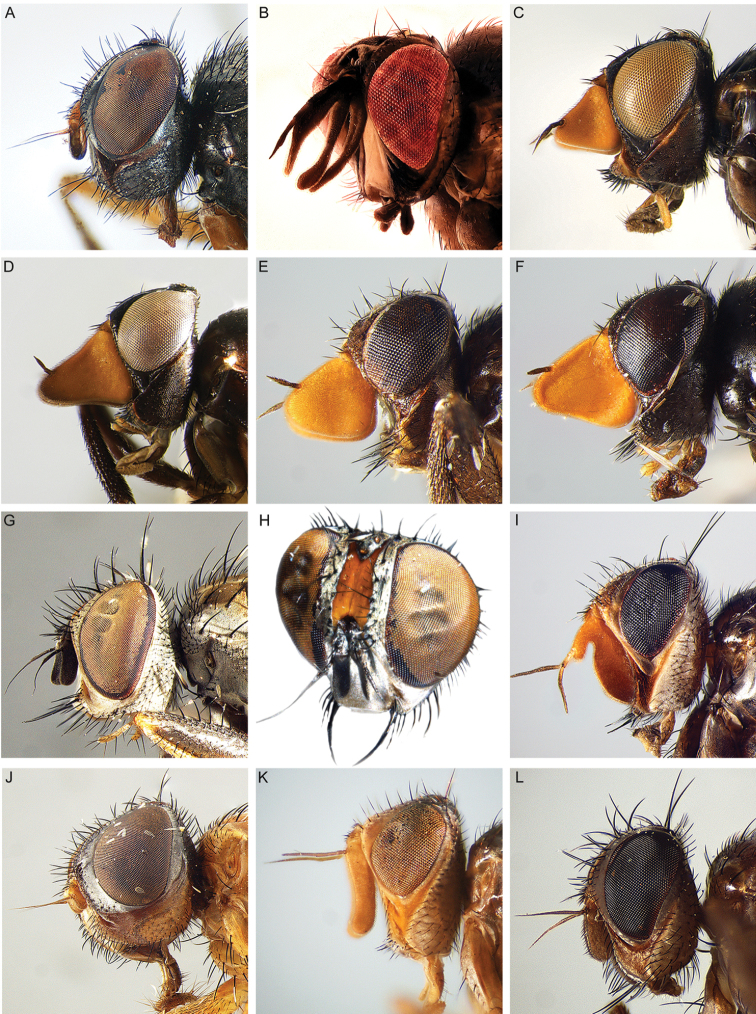
Rhinophoridae, head in lateral view. **A***Apomorphyto inbio* ♂ (Costa Rica) [holotype] **B***Aporeomyia* sp. (Malaysia, Sabah) **C***Axinia arenaria* ♂ (Australia) **D***Axinia brevispica* ♂ (Australia) **E***Axinia lucaris* ♂ (Australia) **F***Axinia zentae* ♂ (Australia) **G, H***Azaisia* sp. ♂ (Portugal, Madeira) **G** lateral view, **H** dorsolateral view **I***Bezzimyia bisecta* ♂ (Costa Rica) **J***Baniassa fascipennis* ♀ (Israel) [paratype] **K***Bixinia collessi* ♂ (Australia) [paratype] **L***Malayia fuscinervis* ♀ (Malaysia, Malay Peninsula).

**Figure 7.**
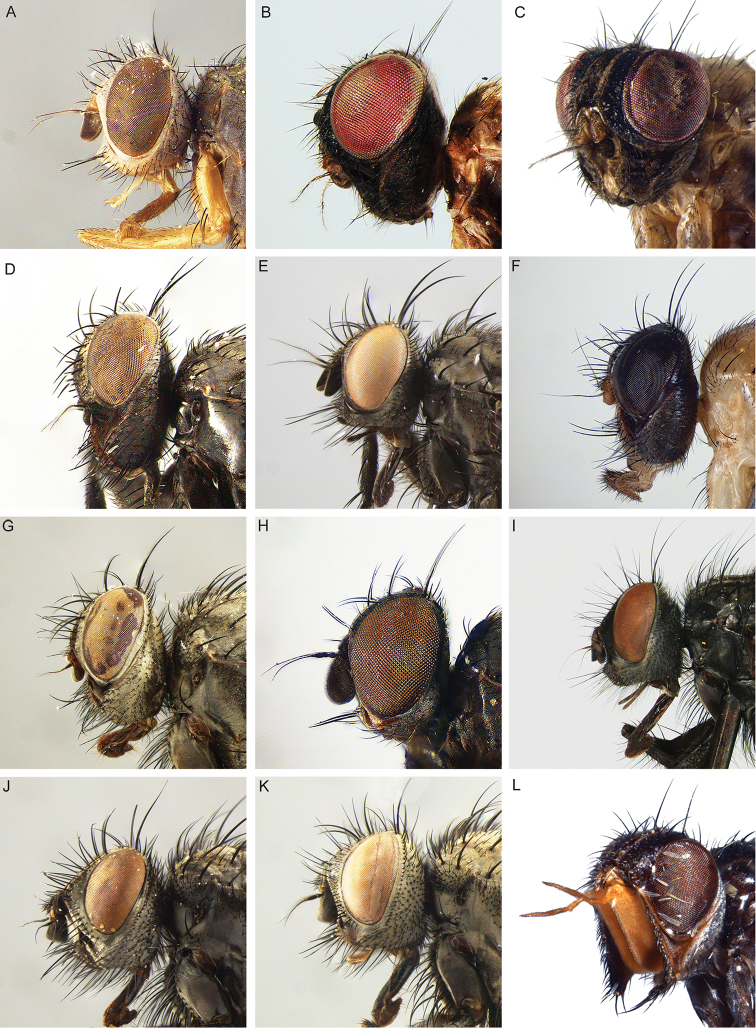
Rhinophoridae, head in lateral view. **A***Macrotarsina longimana* ♂ (Italy) **B, C***Melanophora basilewskyi* ♂ (Kenya) **B** lateral view **C** fronto-lateral view **D***Melanophora roralis* ♂ (Italy) **E***Oplisa tergestina* ♂ (Italy) **F***Parazamimus congolensis* ♂ (Burundi) **G**Paykullia cf. nubilipennis ♂ (Italy) **H***Rhinodonia antiqua* ♂ (New Caledonia) [holotype] **I***Rhinomorinia sarcophagina* ♂ (Italy) **J***Stevenia palermitana* ♂ (Italy) [paratype] **K***Tricogena rubricosa* ♂ (Morocco) **L***Shannoniella setinervis* ♂ (Brazil).

**Figure 8.**
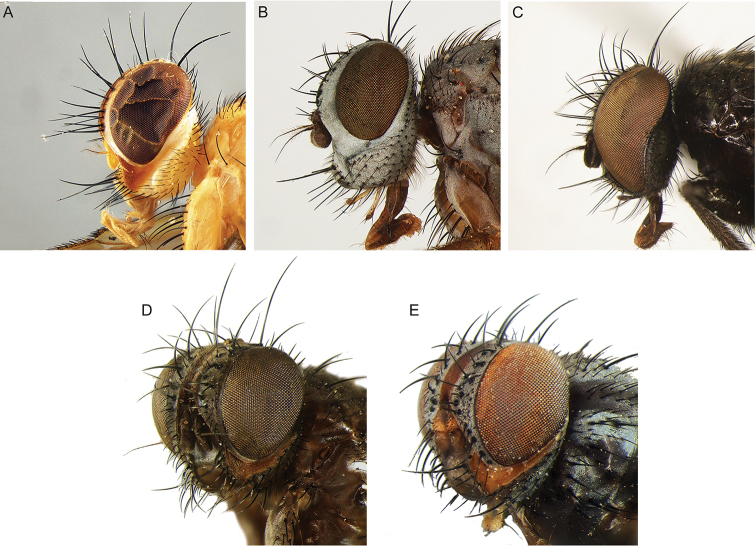
Rhinophoridae, head in lateral view. **A***Tripetidomima lutea* ♂ (Brazil) **B***Tromodesia angustifrons* ♀ (Israel) [paratype] **C***Ventrops milichioides* ♂ (Tanzania) **D***Ventrops hannemariae* ♀ (Tanzania) **E***Ventrops stuckenbergi* ♂ (Namibia) [holotype].

**Figure 9.**
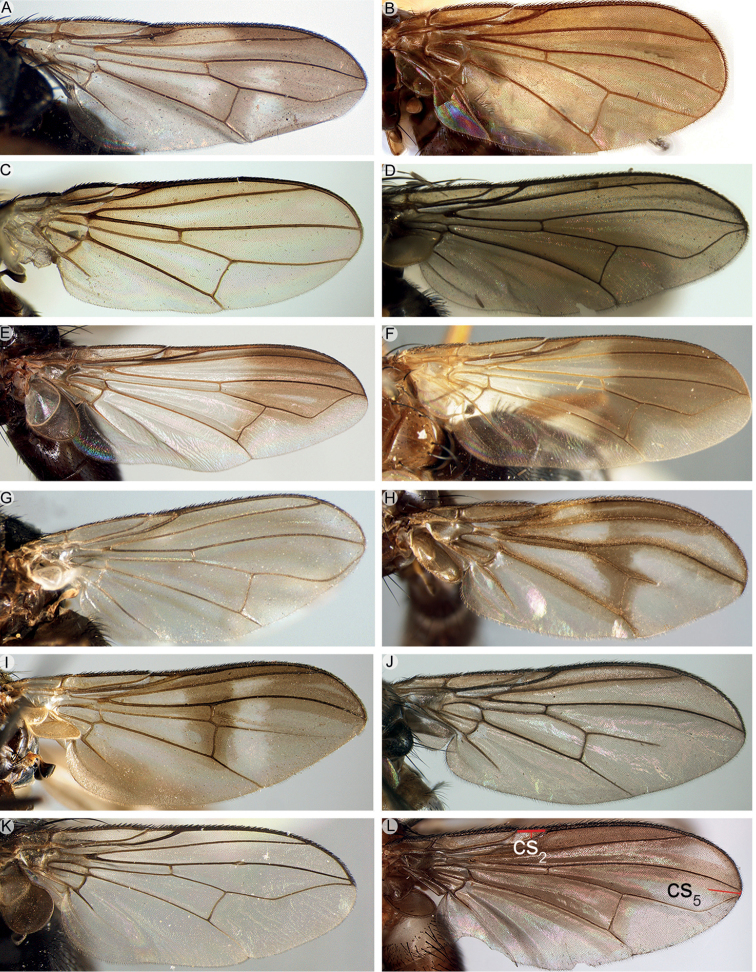
Rhinophoridae, wing. **A***Apomorphyto inbio* ♂ (Costa Rica) [holotype] **B***Aporeomyia* sp. (Malaysia, Sabah) ♂ **C**Axinia cf. brevispica ♀ (Australia) **D***Azaisia* sp. ♂ (Portugal, Madeira) **E, F***Baniassa fascipennis* (Israel) **E** ♂ [paratype] **F** ♀ [paratype] **G***Bixinia collessi* ♂ (Australia) [paratype] **H***Bezzimyia bisecta* ♂ (Costa Rica) **I***Bezzimyia busckii* ♂ (Costa Rica) **J***Melanophora basilewskyi* ♀ (Kenya) **K***Metoplisa carbonaria* ♂ (Israel) **L***Parazamimus congolensis* ♂ (Burundi).

**Figure 10.**
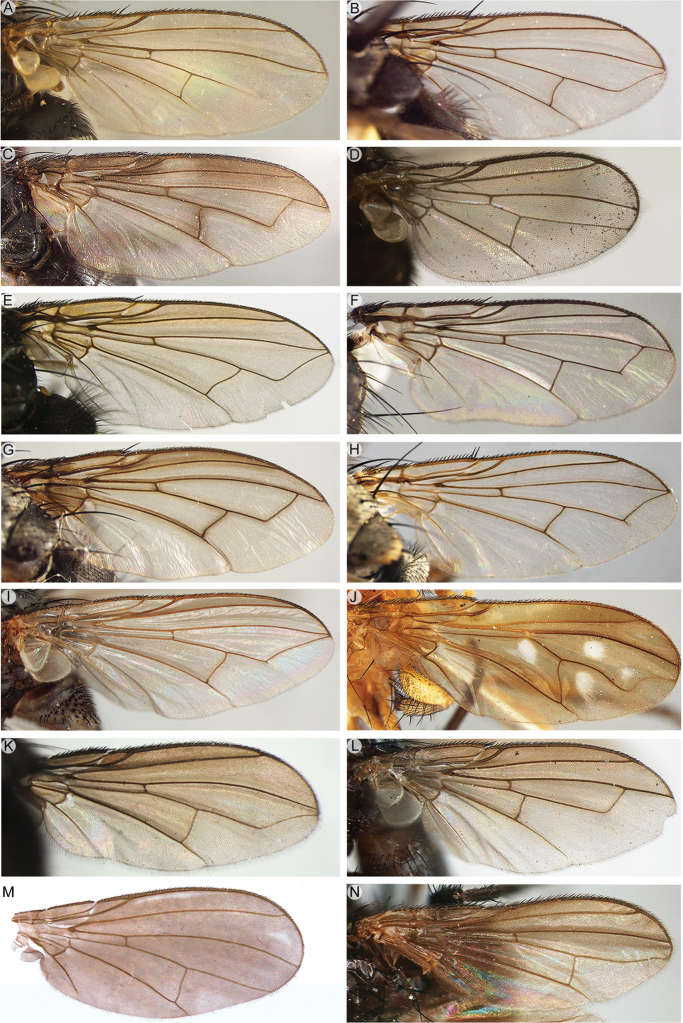
Rhinophoridae, wing. **A***Oplisa aterrima* ♂ (Italy) **B***Oplisa tergestina* ♂ (Italy) **C***Paykullia partenopea* ♂ (Italy) **D***Rhinodonia antiqua* ♂ (New Caledonia) [holotype] **E***Rhinomorinia sarcophagina* ♂ (Italy) **F***Rhinophora lepida* ♂ (Italy) **G***Stevenia etrusca* ♀ (Italy) [paratype] **H***Tricogena rubricosa* ♀ (Morocco) **I***Tromodesia angustifrons* ♀ (Israel) [paratype] **J***Trypetidomima lutea* ♂ (Brazil) **K***Ventrops milichioides* ♂ (Tanzania) **L***Ventrops stuckenbergi* ♂ (Namibia) [holotype] **M***Rhinopeza gracilis* ♂ (Papua New Guinea) [holotype] **N**Polleniidae, wing: *Morinia carinata* ♂ (South Africa).

**Figure 11.**
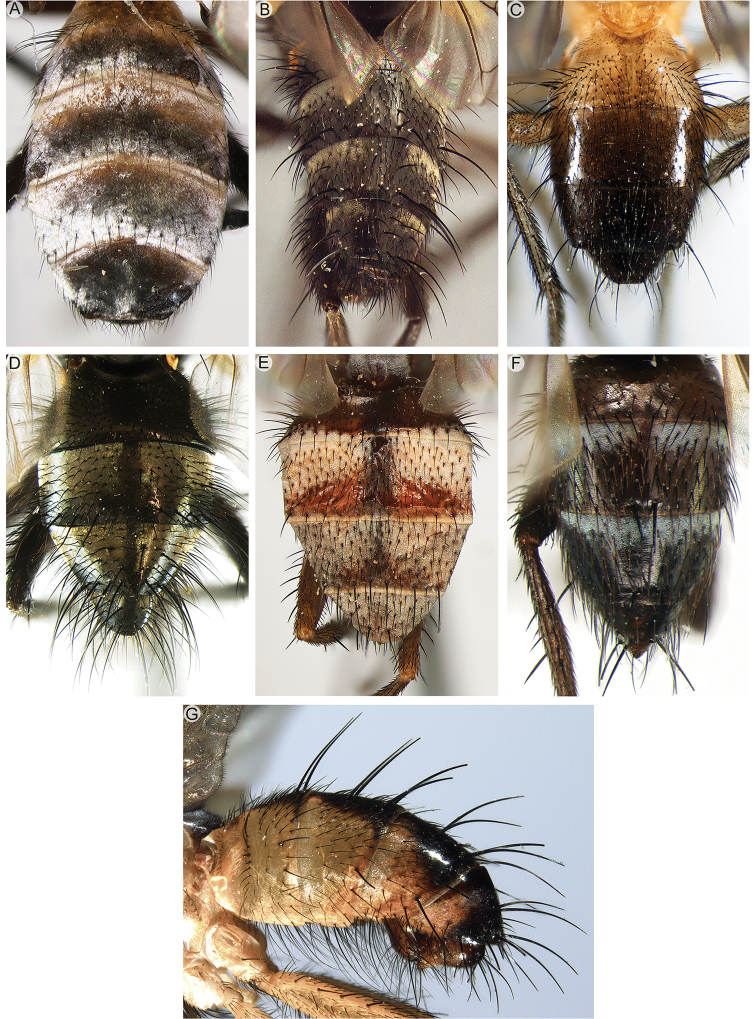
Rhinophoridae, abdomen. **A***Melanophora basilewskyi* ♂ (Kenya) **B***Oplisa tergestina* ♂ (Italy) **C***Parazamimus congolensis* ♂ (Burundi) **D***Rhinomorinia sarcophagina* ♂ (Italy) **E***Tromodesia angustifrons* ♀ (Israel) [paratype] **F***Ventrops stuckenbergi* ♂ (Namibia) [holotype] **G***Queximyia flavipes* ♂ (South Africa). **A–F** dorsal view **G** lateral view.

**Figure 12.**
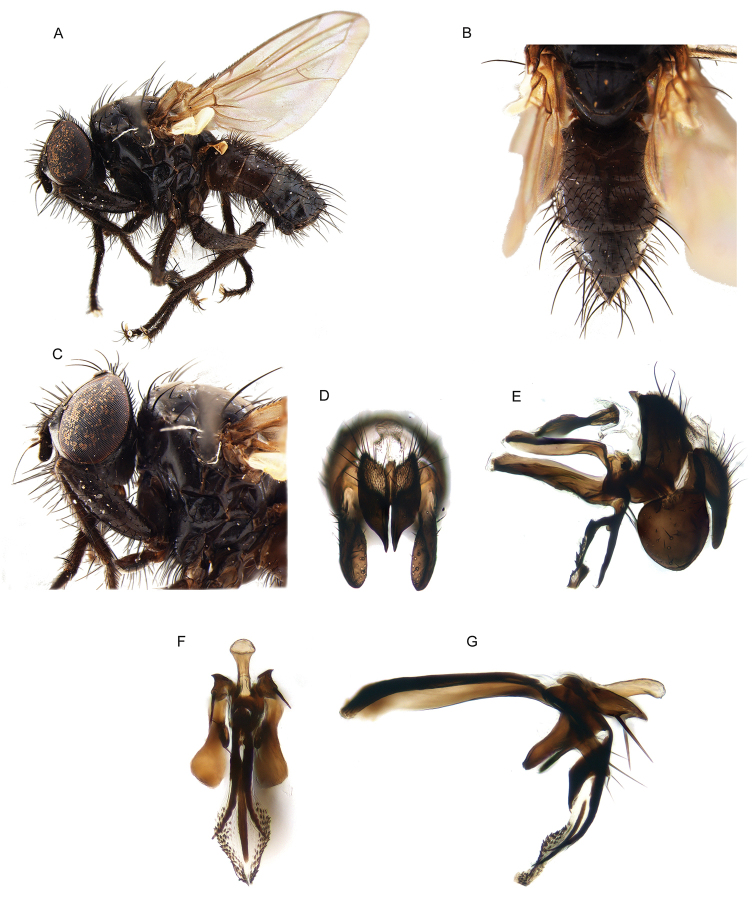
*Melanomyoides capensis* (South Africa). **A** Habitus in lateral view **B** abdomen in dorsal view **C** head in lateral view **D, E** epandrial complex in posterior view (**D**) and lateral view (**E**) **F, G***Phyto adolescens* (Italy), hypandrial complex in posterior view (**F**) and lateral view (**G**).

**Figure 13.**
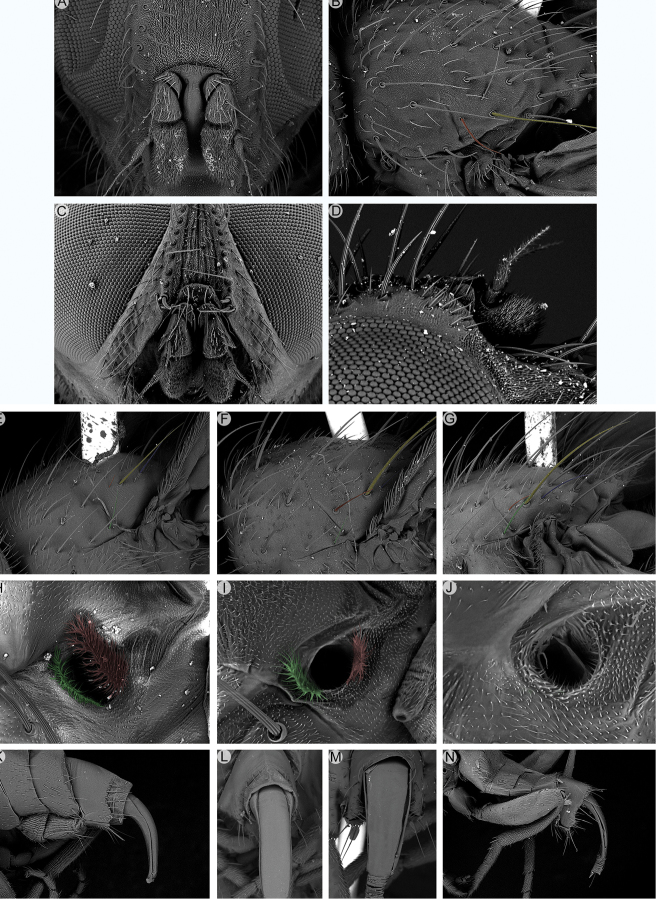
SEM images of diagnostic characters. **A, B**Polleniidae**A***Morinia carinata* ♂ (South Africa) [paratype], detail of head in anterodorsal view **B***Morinia carinata* ♂ (South Africa) [paratype], thorax in dorsolateral view [colour coding as **E–G**] **C–N**Rhinophoridae**C***Phyto adolescens* ♂ (Italy), detail of head in anterodorsal view **D***Melanophora chia* ♂ (Italy), detail of anterior part of head in lateral view **E–G** thorax in dorsolateral view [**B, E–G** colour coding: red = first postsutural supra-alar seta; yellow = second postsutural supra-alar seta; blue = third postsutural supra-alar seta; green = posterior notopleural seta] **E***Parazamimus congolensis* ♀ (Burundi) **F***Queximyia flavipes* ♂ (South Africa) **G***Rhinomorinia sarcophagina* ♂ (Italy) **H–J** metathoracic spiracle [colour coding: green = anterior lappet; red = posterior lappet] **H***Baniassa fascipennis* ♀ (Israel) **I***Rhinomorinia* sp. ♀ (South Africa) **J***Melanophora basilewskyi* ♀ (Kenya) **K–N***Axinia* spp. (Australia), female oviscapt **K, L***Axinia zentae* in lateral view (**K**) and posterior view (**L**) **M, N***Axinia* sp. in lateral view (**M**) and posterior view (**N**).

## New taxa

Four new genera are here erected to accommodate five new species, which do not fit within any of the current generic concepts within Rhinophoridae according to our phylogenetic analysis (see Fig. 20, and “Phylogeny and suprageneric classification” section below). Erecting these new genera is considered superior to alternatives involving generic lumping, as it will facilitate our communication and visualisation of the ever-increasing morphological diversity of the world rhinophorids.

### Afrotropical region

#### Maurhinophora

Taxon classificationAnimaliaDipteraRhinophoridae

Cerretti & Pape gen. nov.

9957236B-D8D6-5DE9-9C02-816FFE8FBCAF

http://zoobank.org/D515BF05-EF3A-4D85-B4D5-959AA3B0EE85

Fig. 14

##### Unambiguous character state changes

(Table 1, Fig. 20). Global apomorphies: **none**; local apomorphies: 13:1, 14:1, 21:2; 30:2, 42:1, 44:1.

##### Diagnosis.

***Head***: head higher than long in lateral view. Facial ridge 1.1 times as long as frons. Ocellar setae virtually absent. Frons approx. 0.9 times as wide as compound eye in dorsal view. Median (= inner) vertical setae strong and crossed. Five medially crossed frontal setae, slightly reclinate, descending to approx. half level of pedicel. Fronto-orbital plate with some short setulae. Two proclinate orbital setae. One upper lateroclinate orbital seta. Parafacial bare, at its narrowest point at most 1.5 times as wide as maximum diameter of arista. Vibrissal angle receding. Vibrissa well developed, arising slightly below level of lower facial margin. Lower facial margin sunken and not visible in lateral view. Facial ridge slightly and evenly convex with a row of setae on lower 2/3, decreasing in size dorsally. Face deeply concave, antennae hidden in lateral view. Antenna long and narrow, much longer than height of gena. Postpedicel narrowly elongated approx. 5 times as long as pedicel. Arista bare (or apparently so). Arista thickened in proximal 2/5–1/2; second aristomere at most as long as wide. Lunule bare. Gena, in profile, approx. 1/5 as high as compound eye. Palpus absent.

***Thorax***: prosternum bare. Postpronotum with three setae arranged in triangle. Three postsutural supra-alar setae (first postsutural supra-alar seta well developed, i.e., longer than posterior notopleural seta and approx. the same size as anterior notopleural seta). Scutellum with one pair of well-developed basal setae and one pair of strong, horizontal and crossed apical setae; basal setae placed at level of apical setae. Anatergite with a tuft of short setulae below lower calypter. Subscutellum moderately swollen, not fully sclerotised. Posterior lappet of metathoracic spiracle larger than anterior lappet (as in *Baniassa*). Lower calypter distinctly tongue-shaped (ground-plan trait of Rhinophoridae) (Fig. 2E). Costal sector cs_2_ setose ventrally. Costal spine not differentiated from general costal setae. Costal sector cs_5_ clearly shorter than costal sector cs_2_. Vein R_1_ entirely setulose dorsally. Vein R_4+5_ with setulae dorsally extending from base to approx. level of bend of vein M_1_. Bend of vein M_1_ well developed, rounded and well removed from wing margin. Crossvein dm-m forming a right angle with proximal section of M_4_. Vein CuA+CuP not reaching wing margin. Preapical anterodorsal seta of fore tibia longer than preapical dorsal seta. Fore tarsus not compressed. Tibiae of mid and hind leg normally developed. Mid tibia with one, short subdistal anterodorsal seta. Hind tibia with 3 dorsal preapical setae.

***Abdomen***: tergites without microtomentum and with relatively strong and suberect general setulae; syntergite 1+2 without median discal setae, tergite 3 with one pair of strong median discal setae, tergites 4 and 5 with a row of strong marginal setae (discal setae not differentiated).

##### Distribution.

Afrotropical – Mauritius.

##### Type species.

*Maurhinophora indoceanica* Cerretti & Pape, sp. nov., by present designation.

##### Etymology.

The generic name is a composite word formed from the first part of the name of the island Mauritius, to which the known species is restricted, and the name *Rhinophora*, which is the type-genus for the family-group name Rhinophoridae. The name should be treated as a feminine noun.

**Figure 14.**
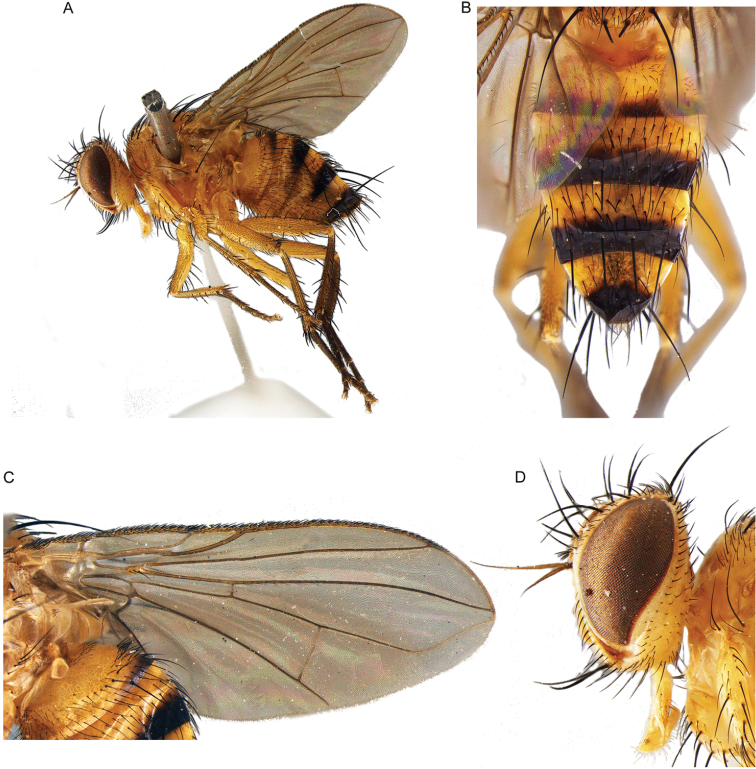
*Maurhinophora indoceanica* gen. et sp. nov. ♀ (Mauritius) [holotype] **A** habitus in lateral view **B** abdomen in dorsal view **C** wing in dorsal view **D** head in lateral view.

#### Maurhinophora indoceanica

Taxon classificationAnimaliaDipteraRhinophoridae

Cerretti & Pape sp. nov.

689435AE-08D6-5CEA-BF52-4F33C7A15C0D

http://zoobank.org/EC960FDE-2EF4-4AFC-8C94-495775E3638A

Fig. 14

##### Type material.

***Holotype*** ♀: Mauritius; /Corps de la Garde, to 2,200’ /4.vi.1971 /A.M. Hudson /B.M. 1971-346. (NHMUK).

##### Description.

**Female**. ***Body length***: 5.5 mm. ***Colouration***: head, thorax (including tegula, basicosta, wing veins and legs) yellow; abdomen mostly yellow with blackish brown transversal bands on posterior 1/3 of syntergite 1+2, and posterior 1/2 of tergites 3–5. ***Head***: frontal vitta as wide as fronto-orbital plate. Two strong proclinate orbital setae (one additional short proclinate orbital seta is present posterior to the main ones). Parafacial narrow with only one setula below lower frontal seta. Lateral (= outer) vertical seta well developed though not strongly differentiated from strongest uppermost postocular setae. Prementum approx. 2 times as long as wide; labella not elongated, normally developed. ***Thorax***: one posthumeral seta (medial); 1 + 3 supra-alar setae (posterior postsutural supra-alar weak); 0 + 2 intra-alar setae; 2(3) + 3 dorsocentral setae; 0 + 1 acrostichal setae. Two strong, diverging katepisternal setae. *Legs*: fore tibia with 1 posterior seta. ***Abdomen***: mid-dorsal depression on syntergite 1+2 confined to anterior half.

##### Distribution.

Afrotropical – Mauritius.

##### Etymology.

The species epithet is derived from the name of the Indian Ocean and should be treated as a Latin adjective.

##### Remarks.

We consider the description of a new species based on a single female as warranted due to the quite remarkable habitus and the occurrence on a small oceanic island, which means that the likelihood of complications due to the lack of male material can be considered low.

### Neotropical region

#### Marshallicona

Taxon classificationAnimaliaDipteraRhinophoridae

Cerretti & Pape gen. nov.

09EC5E6C-17E1-5F89-BC9A-D86D54972C96

http://zoobank.org/2C4548A8-84FB-4AB4-90C2-7CBFE9919839

Figs 1C , 15

##### Unambiguous character state changes

(Table 1, Fig. 20). Global apomorphies: **none**; local apomorphies: 2:1, 9:1, 43:1, 51:1, 52:1, 56:1, 66:0, 68:1.

##### Diagnosis.

***Head***: head higher than long in lateral view. Facial ridge 0.4 times as long as frons. Ocellar setae virtually absent. Frons approx. 0.8 times as wide as compound eye in dorsal view. Median vertical setae strong, subparallel. Six to eight medially crossed frontal setae, slightly reclinate, descending to level of upper margin of scape. Fronto-orbital plate with few short setulae confined to upper half. Four or five proclinate orbital setae (posterior two slightly lateroclinate). One weak upper lateroclinate orbital seta (usually not distinguishable from uppermost frontal setae). Parafacial bare, at its narrowest point approx. as wide as width of postpedicel. Vibrissal angle receding. Vibrissa well developed, arising at level of lower facial margin. Lower facial margin not sunken though not visible in lateral view. Facial ridge concave with decumbent setulae on lower 1/3–2/5. Face slightly concave, antennae not hidden from view in profile. Antenna approx. as long as height of gena. Postpedicel sub-ovoid, approx. 1.5 times as long as pedicel. Arista bottlebrush-like, trichia longer that maximum diameter of arista. Arista thickened in proximal 1/5 or less; second aristomere at most as long as wide. Lunule hidden by inner anterior margins of fronto-orbital plate. Gena, in profile, approx. 1/2 as high as compound eye. Palpus stout, clavate, with a few thin setulae on apical 1/4.

**Figure 15.**
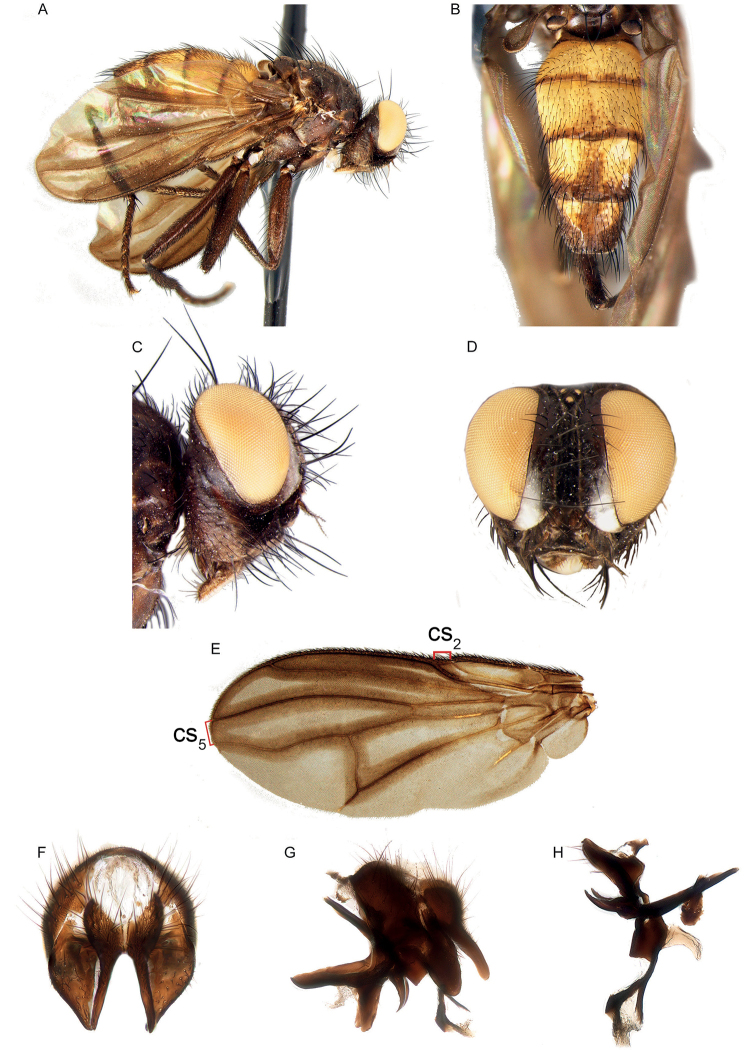
*Marshallicona quitu* gen. et sp. nov. (Ecuador) ♂. **A** Habitus in lateral view **B** abdomen in dorsal view **C** head in lateral view **D** head in frontal view **E** wing in dorsal view **F, G** epandrial complex in posterior view (**F**) and lateral view (**G**) **H** hypandrial complex in lateral view [**A–D** holotype, **E–H** paratype].

***Thorax***: prosternum bare. Postpronotum with two setae. One postsutural supra-alar seta (i.e., first and third post sutural supra-alar setae absent). Scutellum with one pair of well-developed basal setae and one pair of strong, horizontal and crossed apical setae; basal setae placed dorsally with respect to apical setae. Anatergite bare. Subscutellum moderately swollen, not fully sclerotised. Metathoracic spiracular lappets virtually absent. Lower calypter distinctly tongue-shaped (ground-plan trait of Rhinophoridae) (Fig. 2E). Costal sector cs_2_ setose ventrally. Costal spine not differentiated from general costal setae. Costal sector cs_5_ longer than costal sector cs_2_ (Fig. 15E). Vein R_1_ dorsally setose on distal 1/4. Base of R_4+5_ entirely bare. Bend of vein M_1_ shallow, well removed from wing margin. Crossvein dm-m forming an acute angle with proximal section of M_4_. Vein CuA+CuP not reaching wing margin. Preapical anterodorsal seta of fore tibia approx. as long as preapical dorsal seta. Fore tarsus not compressed. Tibiae of mid and hind leg normally developed. Mid tibia with one anterodorsal seta. Hind tibia with 3 dorsal preapical setae.

***Abdomen***: slightly elongated, virtually without microtomentum and without distinct marginal and discal setae.

***Male terminalia*** (Fig. 15F–H): posterior margin of sternite 5 with a deep median notch; lateral lobe rounded posteriorly. Tergite 6 bare, medially divided into two hemitergites; tergite 6 divided from syntergosternite 7+8 by a wide membrane. Connection between sternite 6 and syntergosternite 7+8 on right side membranous. Cerci well developed, not fused medially. Basal 1/3 of cerci convex and covered with short setae; distal 2/3 straight and narrowly digitiform (branches symmetrically diverging and well separated). Surstylus well developed, lobe-like in lateral view; lateral side of surstylus broadly convex at approx. mid length. Surstylus not fused to epandrium. Bacilliform sclerite firmly fused to laterobasal margin of surstylus. Hypandrial arms not fused medially. Connection between phallic guide and pregonite sclerotised. Postgonite without anterior seta. Epiphallus well developed and attached dorsomedially to basiphallus. Extension of dorsal sclerite of distiphallus entirely fused medially into a single sclerite and proximally fused to dorsal sclerite of distiphallus. Median process of ventral sclerotisation of distiphallus present, divided medially into two hemisclerites, which are both proximally fused to ventral plate of distiphallus. Acrophallus simple and scale-like spinules present lateroventrally.

##### Distribution.

Neotropical – Ecuador.

##### Type species.

*Marshallicona quitu* Cerretti & Pape, sp. nov., by present designation.

##### Etymology.

The generic name is a composite word formed from the name of our colleague and friend Steve Marshall, who collected the type series and took the photo of a living specimen (Fig. 1C), and from the Latin noun ‘*icona*’, meaning image, in honour of Steve’s remarkable skills in natural history photography. The name should be treated as a feminine noun.

#### Marshallicona quitu

Taxon classificationAnimaliaDipteraRhinophoridae

Cerretti & Pape sp. nov.

9757A5E7-81F5-517A-80B9-B2D3AB5A833B

http://zoobank.org/2663527B-8BB7-4353-8AFB-774F71581BD2

Figs 1C , 15

##### Type material.

***Holotype*** ♂: Mindo-Bellavista Ecuador /Bellavista Cloud Forest Reserve /0°3'28.57"S, 78°46'6.02"W /2000m 1 May 2001 /S.A. Marshall and S.P.L. Luk (PUCE). ***Paratypes*** 2 ♂♂: same data as holotype (NHMD, MZUR).

##### Description.

**Male**. ***Body length***: 4.5–5.5 mm. ***Colouration***: head mostly black in ground colour, antenna and palpus brown; occiput and posterior 2/3 of fronto-orbital plate shiny without microtomentum, anterior 1/3 of fronto-orbital plate and parafacial covered with silver reflecting microtomentum; thorax (including tegula, basicosta, wing veins and legs) black in ground colour, microtomentum virtually absent; abdomen mainly yellow except brownish posterior margin of tergites 1+2, 3 and 4 and on posterior 1/2–2/3 of tergite 5. Wing membrane infuscate around veins. ***Head***: frontal vitta slightly narrower than fronto-orbital plate (measured at midlength). Parafacial entirely bare below lower frontal seta. Lateral vertical seta not differentiated from strongest uppermost postocular setae. Prementum stout, not longer than wide; labella broad. ***Thorax***: one posthumeral seta (medial); 1 + 1 supra-alar setae; 0 + 2 intra-alar setae; 2(3) + 3 dorsocentral setae; 0–3 + 1 acrostichal setae. One or 2 katepisternal setae. ***Legs***: fore tibia without posterior seta. ***Abdomen***: mid-dorsal depression on syntergite 1+2 confined to anterior half.

##### Distribution.

Neotropical – Ecuador.

##### Etymology.

The species epithet is derived from the Quitu tribe, the pre-Columbian indigenous people who founded the city of Quito, which is now the capital of Ecuador. The name should be treated as a noun in apposition.

#### Neotarsina

Taxon classificationAnimaliaDipteraRhinophoridae

Cerretti & Pape gen. nov.

A9AF30E0-B252-5E11-BC07-7E2CA9011248

http://zoobank.org/6D6A0BBF-2399-43B4-A41C-E4617D37BA03

Figs 16 , 17

##### Unambiguous character state changes

(Table 1, Fig. 20). Global apomorphies: 33:1; local apomorphies: 21:1, 59:1, 72:1.

##### Diagnosis.

***Head***: head higher than long in lateral view. Facial ridge 0.6 times as long as frons. Ocellar setae virtually absent. Frons 0.3–0.5 (male), 0.8–0.9 (female) times as wide as compound eye in dorsal view. Median vertical setae converging or crossed, though very short, at most as long as antenna. Ten to 20 short, medioclinate frontal setae, descending to level of upper margin of scape. Fronto-orbital plate bare or with scattered setulae interspersed between frontal setae. Proclinate orbital setae absent. Upper reclinate orbital seta absent. Parafacial bare, at its narrowest point 0.8–1.2 times as wide as width of postpedicel. Vibrissal angle receding. Vibrissa weak, i.e., barely distinguishable from setae of subvibrissal ridge, arising at level of lower facial margin. Lower facial margin not sunken though not visible in lateral view. Facial ridge concave with decumbent setulae on lower 1/5–2/5. Face slightly concave, antennae not hidden in lateral view. Antenna shorter than height of gena. Postpedicel sub-ovoid, approx. 1.0–1.7 times as long as pedicel. Arista bare. Arista thickened on proximal 1/10–1/5 of its length; second aristomere at most as long as wide. Lunule bare. Gena, in profile, 2/5–1/2 as high as compound eye. Palpus very short 1–2 times as long as wide, bare.

***Thorax***: prosternum bare. Postpronotum with 2–3 setae. One postsutural supra-alar seta (i.e., first and third post sutural supra-alar setae absent). Scutellum with one pair of basal setae and one pair of, crossed, horizontal apical setae; basal setae placed dorsally with respect to apical setae. Anatergite bare. Subscutellum moderately swollen or flat, not fully sclerotised. Metathoracic spiracular lappets small, subequal in size and directed outwards. Lower calypter distinctly tongue-shaped (ground-plan trait of Rhinophoridae) (Fig. 2E). Costal sector cs_2_ usually setose ventrally. Costal spine not differentiated from general costal setae. Costal sector cs_5_ approx. as long as costal sector cs_2_. Vein R_1_ dorsally bare. Base of R_4+5_ entirely bare. Bend of vein M_1_ indistinct; i.e., M_1_ evenly curved forward without forming a distinct bend. Crossvein dm-m forming a right angle with proximal section of M_4_. Vein CuA+CuP not reaching wing margin. Preapical anterodorsal seta of fore tibia longer than preapical dorsal seta. Fore tarsus strongly laterally compressed in both sexes. Tibiae of mid and hind legs laterally compressed and distinctly keeled dorsally. Mid tibia without anterodorsal setae. Hind tibia with three dorsal preapical setae.

***Abdomen***: lightly elongated, varying from slightly microtomentose to virtually without microtomentum. Marginal and discal setae not differentiated from general setulae.

***Male terminalia***: posterior margin of sternite 5 with a deep median notch; lateral lobe rounded posteriorly. Tergite 6 plate-like, with median marginal setae; tergite 6 divided from syntergosternite 7+8 by a membrane. Connection between sternite 6 and syntergosternite 7+8 fused on right side. Cerci well developed, basally broad, narrowing toward apex and apically pointed, well separated medially. Surstylus well developed, sub-triangular in lateral view; lateral side of surstylus not or only slightly convex. Surstylus fused to epandrium. Bacilliform sclerite articulated (i.e., not fused) to laterobasal margin of surstylus. Hypandrial arms not fused medially. Connection between phallic guide and pregonite membranous. Postgonite without anterior seta. Epiphallus well developed and attached dorsomedially or dorsomedially to basiphallus. Extension of dorsal sclerite of distiphallus divided medially into two hemisclerites which are proximally not fused to dorsal sclerite of distiphallus. Median process of ventral sclerotisation of distiphallus present, not interrupted, i.e., running from the ventral plate to tip of phallus, and not divided medially. Acrophallus simple and scale-like spinules not differentiated.

##### Distribution.

Neotropical – Peru, Trinidad and Tobago (Trinidad).

##### Type species.

*Neotarsina caraibica* Cerretti & Pape, sp. nov., by present designation.

##### Etymology.

The generic name is a composite word formed from the Latin word ‘*neo*’ meaning new, and ‘*tarsina*’ [from Latin ‘*tarsus*’ and Greek: ‘*tarsos*’, the flat part of a human foot] as in the last part of the genus-group name *Macrotarsina*, in reference to the modified, laterally-compressed fore tarsus characterising the two new species described below. The name should be treated as a feminine noun.

**Figure 16.**
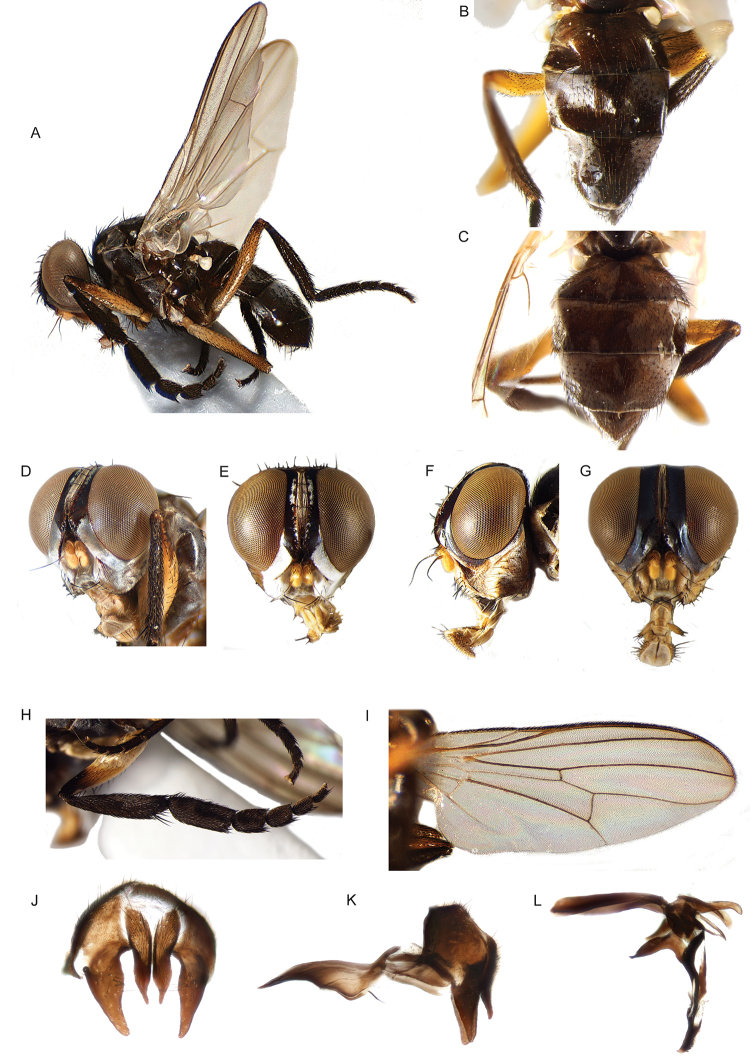
*Neotarsina andina* gen. et sp. nov. (Peru). **A** Female habitus in lateral view **B** male abdomen in dorsal view **C** female abdomen in dorsal view **D, E** male head in lateral view (**D**) and frontal view (**E**) **F, G** female head in lateral view (**F**) and frontal view (**G**) **H** female fore tibia and tarsus **I** male wing in dorsal view **J–L** epandrial complex in posterior view (**J**) and lateral view (**K**) **L** hypandrial complex in lateral view [**B, D–E, I–L** holotype; **A, C, F, G** paratype].

**Figure 17.**
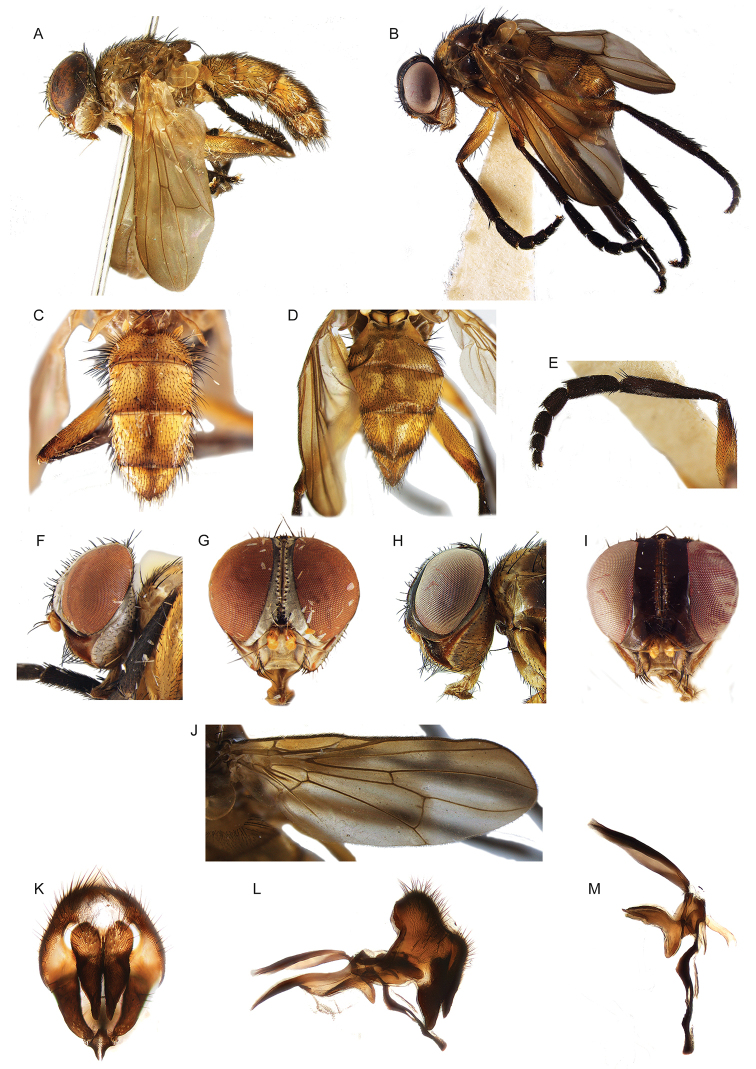
*Neotarsina caraibica* gen. et sp. nov. (Trinidad and Tobago). **A** Male habitus in lateral view **B** female habitus in lateral view **C** male abdomen in dorsal view **D** female abdomen in dorsal view **E** female fore tibia and tarsus **F, G** male head in lateral view (**F**) and frontal view (**G**) **H, I** female head in lateral view (**H**) and frontal view (**I**) view **J** male wing in dorsal view **K–M** mpandrial complex in posterior (**K**) and lateral (**L**) view **M** hypandrial complex in lateral view [**A, C, F–G, J** holotype; **B, D–E, H–I, K–M** paratypes].

#### Neotarsina andina

Taxon classificationAnimaliaDipteraRhinophoridae

Cerretti & Pape sp. nov.

934AFC75-60DD-56C7-852B-C3C1F577C1F1

http://zoobank.org/8E091767-9298-48A6-A49C-73594CCEFA86

Fig. 16

##### Type material.

***Holotype*** ♂: Peru: Raymondi /monoculture (loc. 11) /6°45'21"S, 79°51'05"W /J. Krausová, 15.v.09 (CULSP). ***Paratype*** ♀: PERU: Pimental, 165 m /agroforest (loc. 81) /8°31'30"S, 74°46'30"W /J. Krausová, 1.xi.10 (CULSP).

##### Notes on type specimens.

The holotype lacks postpronotal and scutellar setae but is otherwise in good condition. The paratype is in fair general condition but lacks most of the setae on the thorax.

##### Description.

**Male**. ***Body length***: ca. 4 mm. ***Colouration***: head mostly black except antenna and palpus which are pale yellow, parafacial, gena and lower occiput covered with dense silver reflecting microtomentum; fronto-orbital plate polished shiny black, except narrowing microtomentose along medial margin; thorax entirely black or dark brown in ground colour; thoracic pleura covered with silver reflecting microtomentum; femora mostly yellow except dark brown proximally, tibiae and tarsi dark brown; calypters whitish; tegula and basicosta pale yellow; wing membrane hyaline; halter yellow; abdomen black in ground colour, tergites 3 and 4 laterally covered with silver reflecting microtomentum. ***Head***: frontal vitta measured at mid-length approx. 1.7 times as wide as fronto-orbital plate at same level. Postpedicel 1.4–1.7 times as long as pedicel. First and second aristomeres approx. as long as wide. Frontal setae medioclinate, slightly proclinate near antennal insertion. Median vertical setae short, approx. 0.2 times as long as compound eye height. Parafacial approx. 1.2 times as wide as postpedicel. Facial ridge bare or with few short setae above vibrissa. Gena, in profile, 2/5 as high as compound eye. Frons 0.5 times as wide as eye in dorsal view. Prementum stubby, approx. 2 times as long as wide; labella not elongated, normally developed. Palpus short, clavate. ***Thorax***: two postpronotal setae; two posthumeral setae; 0 + 1 supra-alar setae (i.e., first and third postsutural supra-alar setae absent); 0 + 2 intra-alar setae; 2 + 3 dorsocentral setae (first presutural dorsocentral barely distinguishable from general setulae); one presutural acrostichal seta; postsutural acrostichal setae apparently not differentiated. Fore tibia approx. 1.5 times as long as first tarsomere. Costal sector cs_2_ nearly bare ventrally. ***Abdomen***: Tergite 5 very short, approx. 0.5–0.6 times as long as tergite 4. ***Male terminalia***: Surstylus narrowly triangular in lateral view. Cerci relatively narrow with a clear, though shallow, bottleneck restriction at approx. mid length; apical 1/5 of cerci strongly narrowing and pointed. **Female**. ***Body length***: 5 mm. Female differs from male as follows: ***Head***: fronto-orbital plate entirely shiny and parafacial with less dense microtomentum. Frontal vitta measured at mid-length approx. 2.0 times as wide as fronto-orbital plate at same level. Frons 0.8 times as wide as eye in dorsal view.

##### Distribution.

Neotropical – Peru.

##### Etymology.

The species epithet is derived from the name of the Andes mountain range and should be treated as a Latin adjective.

#### Neotarsina caraibica

Taxon classificationAnimaliaDipteraRhinophoridae

Cerretti & Pape sp. nov.

951081C8-419F-59ED-984B-BBF432891012

http://zoobank.org/547E1F61-4D43-4C42-A32F-D0D56E83FB9D

Fig. 17

##### Type material.

***Holotype*** ♂. TRINIDAD: Curepe /Santa Margarita /malaise trap /12–14.vi.1972 //B.R. Pitkin coll. /BMNH(E) 1997-41 (NHMUK). ***Paratypes***: 1 ♂: same data and repository as holotype; 1 ♀: Simla, Trin. [Trinidad and Tobago] /II-16-1966 /W. D. Duckworth (USNM, unique specimen identifier: USNMENT01519745).

##### Description.

**Male**. ***Body length***: ca. 4.5–5.5 mm. ***Colouration***: head mostly dark brown, except antenna and palpus which are yellow and genal dilation, which is brownish; head evenly covered with grey reflecting microtomentum. Scutum brown with three or four pale longitudinal vittae; thoracic pleura covered with silver reflecting microtomentum; tegula, basicosta light brown, calypters and wing membrane slightly infuscate; halter yellow; coxae and femora yellow; tibiae brown; tarsi dark brown; abdomen yellowish to light brown, weakly microtomentose. ***Head***: frontal vitta 0.5–0.7 times as wide as fronto-orbital plate, both measured at approx. midlength. Ocellar triangle setulose, without ocellar setae. Postpedicel approx. 1.0–1.2 times as long as pedicel. Arista thickened in proximal 1/8–1/10; first and second aristomere approx. as long as wide. Frontal setae medioclinate, slightly proclinate near base of antenna. Median vertical setae short, 0.2–0.3 times as long as height of compound eye, crossed medially. Parafacial 0.8–0.9 times as wide as postpedicel. Facial ridge with few short setae above vibrissa. Gena, in profile, 2/5–1/2 as high as compound eye. Frons approx. 0.3 times as wide as compound eye in dorsal view. Prementum stubby, 1–2 times as long as wide; labella not elongated, normally developed; palpus exceptionally reduced. ***Thorax***: two postpronotal setae; one posthumeral seta; 0 + 1 supra-alar setae (i.e., first and third postsutural supra-alar setae absent); 1 + 1–2 intra-alar setae; 2 + 3 dorsocentral setae (first presutural dorsocentral barely distinguishable from general setulae); acrostichal setae not differentiated. Fore tibia approx. 2 times as long as first protarsomere. Hind tibia with 3 dorsal preapical setae and 3–4 well-developed anterodorsal and posterodorsal setae. Costal sector cs_2_ setulose ventrally. ***Abdomen***: tergite 5 approx. as long as tergite 4. ***Male terminalia***: surstylus triangular in lateral view; cerci stout with a slight restriction at approx. mid length; apical 1/5 of cerci not pointed. **Female**. Female differs from male as follows: ***Head***: fronto-orbital plate entirely shiny and parafacial with less dense microtomentum. Frontal vitta approx. 0.2 times as wide as fronto-orbital plate in female. Parafacial 1.2 times as wide as postpedicel. Frons approx. 0.9 times as wide as compound eye in dorsal view.

##### Distribution.

Neotropical – Trinidad and Tobago (Trinidad).

##### Etymology.

The species epithet is derived from the Spanish word for Caribbean and should be treated as a Latin adjective.

### Oriental region

#### Kinabalumyia

Taxon classificationAnimaliaDipteraRhinophoridae

Cerretti & Pape gen. nov.

0C026A8A-9BF9-58C6-87B0-D02322EF9DF6

http://zoobank.org/5E01603E-D354-4B84-BA64-0064DD960B14

Fig. 18

##### Unambiguous character state changes

(Table 1, Fig. 20). Global apomorphies: 81:1; local apomorphies: 21:1, 53:1, 59:1.

##### Diagnosis.

***Head***: head higher than long in lateral view. Facial ridge approx. 1.3 times as long as frons. Ocellar setae present though small. Frons 0.9–1.1 times as wide as compound eye in dorsal view. Median vertical setae strong and slightly converging. Two or three slightly convergent and reclinate frontal setae, descending to approx. half level of pedicel. Fronto-orbital plate nearly bare with one proclinate orbital seta (no sexual dimorphism). One upper lateroclinate orbital seta. Parafacial bare, at its narrowest point approx. twice as wide as maximum diameter of arista. Vibrissal angle strongly receding. Vibrissa well developed, arising slightly below the level of lower facial margin. Lower facial margin not sunken and slightly visible in lateral view. Facial ridge strongly concave with two or three short setulae above vibrissa. Face concave but antennae not hidden and clearly visible in lateral view. Antenna long and wide (in lateral view), much longer than height of gena. Postpedicel 4.5–6.5 times as long as pedicel. Postpedicel axe-shaped in male, more or less stick-like in female. Arista bare (or apparently so). First and second aristomere slightly thickened and strongly elongated. Lunule bare. Gena, in profile, approx. 3/5 (male), 1/2 (female) as high as compound eye. Palpus pretty short, dark brown.

***Thorax***: prosternum bare. Postpronotum with two setae. Two postsutural supra-alar setae (first postsutural supra-alar seta absent). Scutellum with one pair of strong lateral setae and one pair of short, crossed and horizontal apical setae. Anatergite with a tuft of short setulae below lower calypter. Subscutellum moderately swollen, mostly, though not entirely, sclerotised. Metathoracic spiracular lappets nearly undeveloped. Lower calypter distinctly tongue-shaped (ground-plan trait of Rhinophoridae) (Fig. 2E). Costal sector cs2 setose ventrally. Costal spine not differentiated from general costal setae. Costal sector cs_5_ clearly longer than costal sector cs_2_. Vein R_1_ entirely bare. Vein R_4+5_ bare dorsally. Bend of vein M_1_ absent. Crossvein dm-m forming a right angle with proximal section of M_4_. Vein CuA+CuP reaching wing margin. Preapical anterodorsal seta of fore tibia slightly longer than preapical dorsal seta. Fore tarsus not compressed. Tibiae of mid and hind leg normally developed. Hind tibia with three dorsal preapical setae.

***Abdomen***: tergites virtually without microtomentum and with relatively strong and recumbent general setulae; syntergite 1+2, tergites 3 and 4 with a pair of strong median marginal setae, without median discal setae, tergite 5 with strong marginal setae without discal setae.

***Male terminalia***: posterior margin of sternite 5 slightly concave, almost straight. Tergite 6 plate-like, with median marginal setae; tergite 6 and syntergosternite 7+8 fused. Connection between sternite 6 and syntergosternite 7+8 fused on right side. Cerci well developed and entirely fused medially into a syncercus. Surstylus long, narrow (almost stick-like), slightly enlarged distally and gently curved anteriorly. Surstylus not fused to epandrium. Bacilliform sclerite articulated (i.e., not fused) to laterobasal margin of surstylus. Hypandrial arms not fused medially. Connection between phallic guide and pregonite membranous. Pregonite well developed and lobe-like. Postgonite without anterior seta. Epiphallus well developed apically, pointed and attached dorsomedially to basiphallus. Extension of dorsal sclerite of distiphallus divided medially into two hemisclerites which are proximally fused to dorsal sclerite of distiphallus. Median process of ventral sclerotisation of distiphallus present, divided longitudinally and interrupted proximally, i.e., not connected to ventral plate. Acrophallus simple and scale-like spinules not differentiated.

##### Distribution.

Oriental – Indonesia (Bali), Malaysia (Sabah), Philippines (Palawan).

##### Type species.

*Kinabalumyia pinax* Cerretti & Pape, sp. nov., by present designation.

##### Etymology.

The generic name is a composite word formed from the name of the type locality of the type species, Mount Kinabalu, and from the Greek word *μύγα* (miga), meaning fly. The name should be treated as a feminine noun.

**Figure 18.**
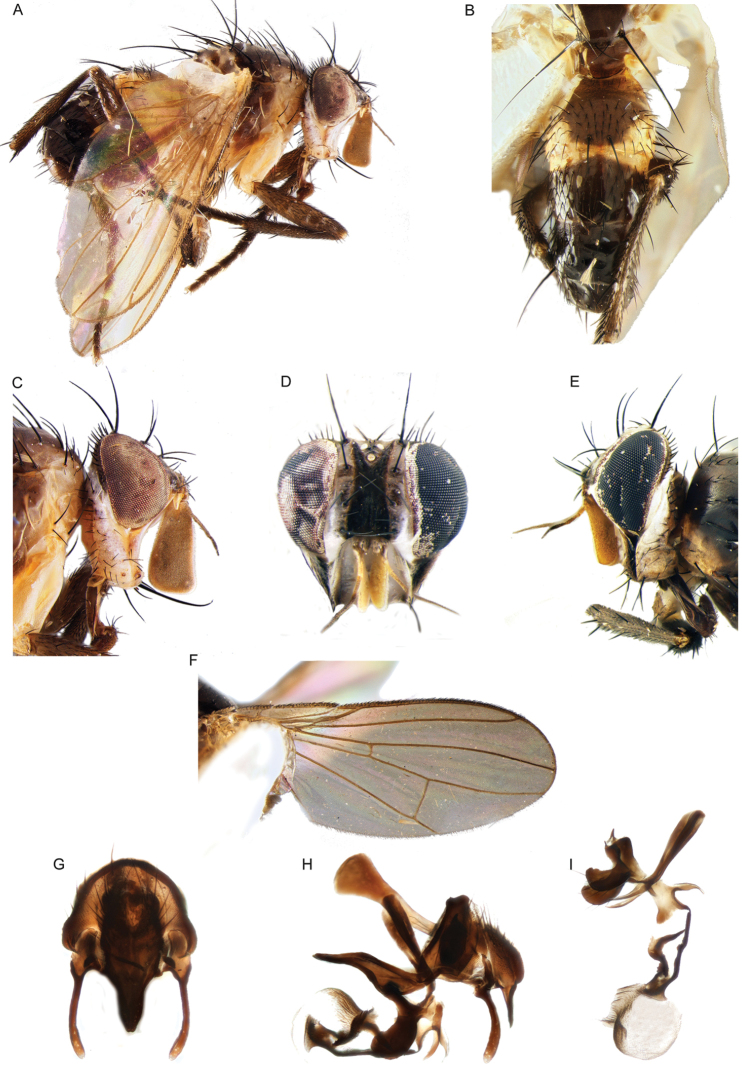
*Kinabalumyia* gen. nov. **A–C, F–I***Kinabalumyia pinax* sp. nov. (Malaysia, Sabah) **D–E***Kinabalumyia* sp. ♀ (Philippines) **A***K. pinax* sp. nov., habitus in lateral view **B***K. pinax* sp. nov., abdomen in dorsal view **C***K. pinax* sp. nov., head in lateral view **D***Kinabalumyia* sp. ♀, head in frontal view, ♀ **E***Kinabalumyia* sp. ♀, head in lateral view **F***K. pinax* sp. nov., wing **G–I***K. pinax* sp. nov.: epandrial complex in posterior (**G**) and lateral (**H**) view **I** hypandrial complex in lateral view [**A–C** holotype; **F–I** paratypes].

#### Kinabalumyia pinax

Taxon classificationAnimaliaDipteraRhinophoridae

Cerretti & Pape sp. nov.

6FC2DAF6-8150-52DA-A94A-7D659C54DD16

http://zoobank.org/A664B712-8059-4F62-80D0-6315FEE2B19F

Fig. 18A–C, F–I

##### Type material.

***Holotype*** ♂: At light //SABAH: Mt Kinabalu /Mesilau 14.II.1964. J. Smart. /Royal Soc. Exped. /B.M. 1964-250 (NHMUK). ***Paratype*** ♂: same data and repository as holotype.

##### Description.

**Male**. ***Body length***: 3.5–3.7 mm. ***Colouration***: fronto-orbital plate and upper occiput blackish-brown, frontal vitta black, remainder of head yellow; head covered with silver reflecting microtomentum. Scape and pedicel brown, postpedicel blackish-brown, palpus brown; postpronotum, proepisternum, proepimeron, and prosternum pale yellow, scutum, notopleuron, scutellum and subscutellum brown; anepisternum and upper anterior part of katepisternum brown, remaining sclerites pale brown except anatergite which is dark brown; tegula brown, basicosta and wing veins yellow; coxae and trochanters pale yellow, femora, tibiae and tarsi blackish-brown; abdomen can vary from entirely black to mostly black except yellow along posterior margin, on sides of syntergite 1+2 and along anterior margin of tergite 3; microtomentum virtually absent. Wing membrane hyaline. ***Head***: frontal vitta well developed and slightly wider than fronto-orbital plate (both measured at midlength). Parafacial entirely bare below lower frontal seta. Lateral vertical seta not differentiated from strongest uppermost postocular setae. Prementum stout, not longer than wide; labella broad. ***Thorax***: one posthumeral seta (medial); 1 + 1 supra-alar setae; 0 + 1–2 intra-alar setae; 2(3) + 3 dorsocentral setae; acrostichal setae not differentiated. One or two katepisternal setae. *Legs*: fore tibia without posterior seta. ***Abdomen***: mid-dorsal depression on syntergite 1+2 confined to anterior half.

##### Distribution.

Oriental – Malaysia (Sabah).

##### Etymology.

The species epithet, which should be treated as a noun in apposition, is derived from the Greek noun *pinax*, meaning painting, in reference to the remarkable colour pattern of the thorax and abdomen.

#### Kinabalumyia

Taxon classificationAnimaliaDipteraRhinophoridae

sp. 1 (unidentified)

364446D2-EC21-5D00-A6CA-333BE060E0FD

Fig. 18D, E

##### Material examined.

1 ♀: Philippines, Palawan /Mantalingajan /Tagembung 1150 meter /18 Sept. 1961 /Noona Dan Exp. 61-62 //Caught by /Mercury-light /18.00–06.00 (NHMD).

##### Remarks.

This female specimen, in fair condition, strongly resembles the two males from Sabah described as *K. pinax* above and may be conspecific with them. However, the sparse material available, the lack of females from the type locality, and the wide geographic separation provide sufficient taxonomic uncertainty to suggest caution. We therefore prefer to treat this specimen as unidentified awaiting further material.

##### Distribution.

Oriental – Philippines (Palawan).

#### Kinabalumyia

Taxon classificationAnimaliaDipteraRhinophoridae

sp. 2 (undescribed)

33FA78D6-E493-5D84-833C-2E01F9134C41

##### Material examined.

**Figure 19.**
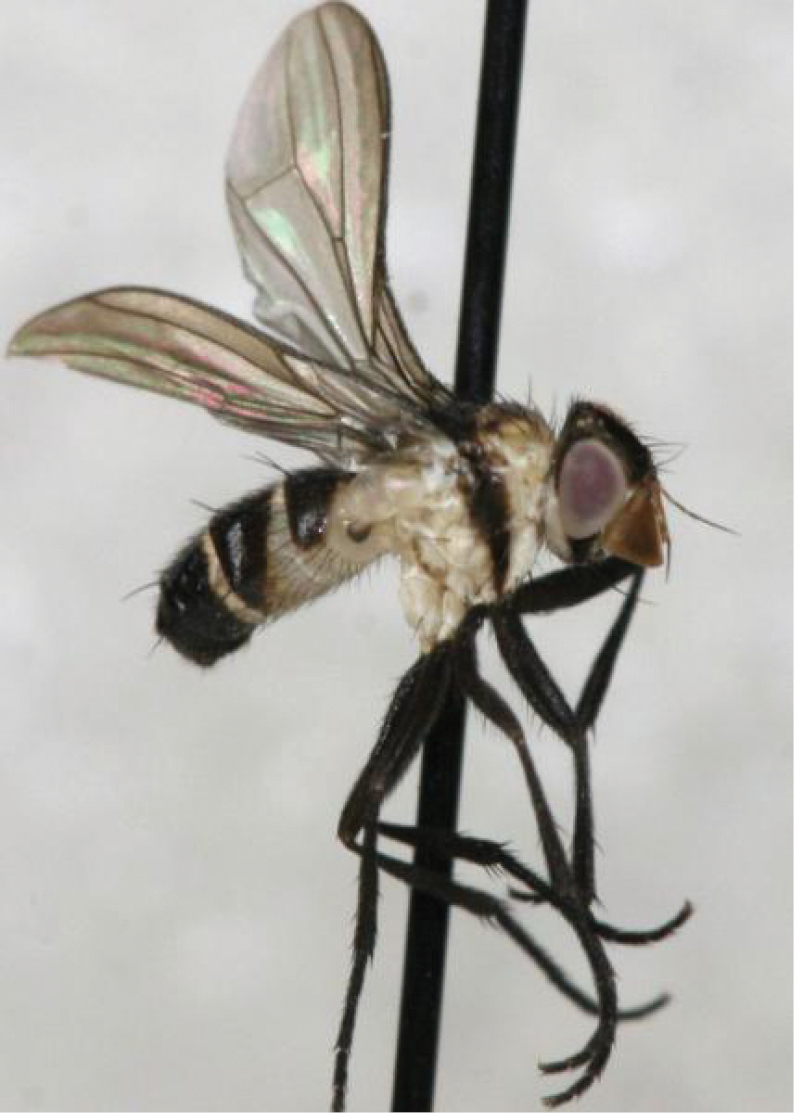
*Kinabalumyia* sp. 2, undescribed species from Indonesia, Bali (ZMUM).

1 ♂: Indonesia, Bali, Bratan L. env., 1250m, Febr, 2014, O. Kosterin (ZMUM); photo only (Fig. 19), image also available from www.diptera.info (photo_id=9408).

##### Remarks.

The photo shows a male specimen assessed as belonging to the genus here newly described as *Kinabalumyia*. Shape of the antenna and colouration of the body suggest that it is not conspecific with *K. pinax* and therefore represents an undescribed species.

##### Distribution.

Oriental – Indonesia (Bali).

### Diversity and distribution

The Palaearctic Region hosts by far the most diverse rhinophorid fauna, with 92 of the 177 described species, and these are heavily concentrated to the western part. The Afrotropical Region contains 33 species, followed by the Australasian and Neotropical regions with 24 and 21 species, respectively. Only seven species (plus the two undescribed) have been recorded from the Oriental Region, and the by far most species-poor region is the Nearctic with only four native rhinophorids, all belonging to the genus *Bezzimyia*. This genus reaches its peak of diversity in the Neotropical Region. Indeed, the distribution of the Nearctic species of *Bezzimyia* is limited to the southernmost parts of the USA (Arizona, Texas, Georgia, Florida), corresponding with the range of the few native Nearctic woodlice (see Jass and Klausmeier 2000, Schmalfuss 2003), which are the most probable hosts of *Bezzimyia* and relatives. The near-absence of native woodlice in most of the temperate parts of North America has been given as in need of explanation (Jass and Klausmeier 2000), although it is rather the remarkable diversity of woodlice in the western Palaearctic that asks for scrutiny (“Paradoxically, their present centre of distribution and evolution is the region of the Mediterranean Sea”, Hatch 1947: 175; see also Sfenthourakis and Hornung 2018).

Faunistic connections are scarce among biogeographic regions, as virtually no rhinophorid species is so far known to be distributed across two or more regions, with the exclusion of a few anthropogenic introductions outside their native distributional range. In particular, three rhinophorids have been introduced from Europe to the Americas (Downes 1965, Mulieri et al. 2010, O’Hara et al. 2015, Wood et al. 2018), namely: *Melanophora roralis* (Linnaeus), which is now widespread in the New World; *Stevenia deceptoria* (Loew), recently reported from Ohio (USA) and northern Argentina; and *Phyto discrepans* Pandellé, known from Canada. While the two former species are now well established, little is known about the status of the latter. Moreover, *Melanophora roralis*, which is a native West-Palaearctic element, has been introduced to Japan and Cape Verde Islands, where it is now established (Crosskey 1977, Kato and Tachi 2016).

### Phylogeny and suprageneric classification

Monophyly of Rhinophoridae and differences with previous analyses

As stressed by Cerretti and Pape (2012) and Cerretti et al. (2014a), the rhinophorid phylogeny based on currently available morphological data is poorly resolved, while a comprehensive phylogenetic study based on molecular data is still under completion (Gisondi et al., unpublished). Nevertheless, refined morphological evidence, coupled with new discoveries, are producing interesting results. Our taxon sample includes at least one species of all recognised rhinophorid genera, thereby comprising 14 more ingroup species than that of Cerretti et al. (2014a) (Table 1, asterisked names).

**Figure 20.**
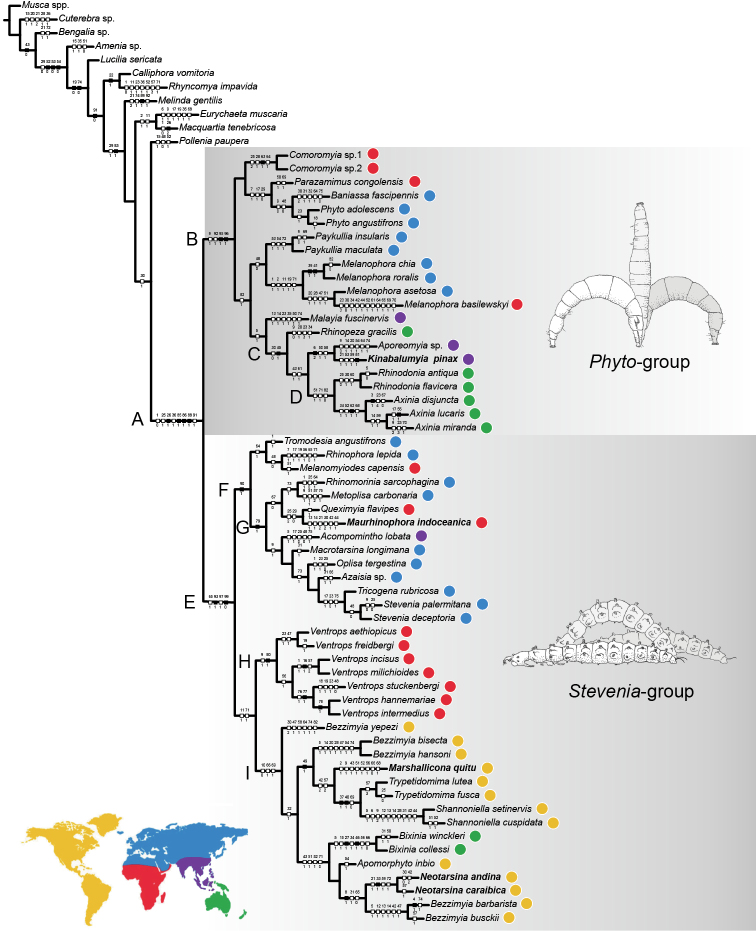
Phylogeny of Rhinophoridae based on morphological evidence. Single tree obtained under IW (total fit: 62.33), enforcing a k-value of 4, with unambiguous character state changes mapped above branches [black squares: global apomorphies (i.e., uncontradicted and unreversed apomorphic character states; white squares: local apomorphies (i.e., homoplasious character state due to convergence or reversal)] and new taxa highlighted in bold.

Our analyses reconstructed Rhinophoridae (clade A) as monophyletic based on eight unambiguous character state changes [four traceable in the adult: arista with short trichia (1:0, local apomorphy), first postsutural supra-alar seta short (25:1, local apomorphy), subscutellum moderately swollen (26:1, global apomorphy), lower calypter tongue-shaped (36:1, local apomorphy); four in the first instar larva: antenna long and tapering (85:1, global apomorphy), posterior part of anal division modified as a terminal sucker (86:1, global apomorphy), mandibles toothed or serrated (88:1, global apomorphy), and parastomal bar of cephaloskeleton long and slender (91:1, local apomorphy)], confirming the split of the family into two main subclades: the *Phyto* group (clade B), the first instar larvae of which move by somersaulting, and the *Stevenia* group (clade E), whose first instars move in a leech-like crawling fashion (see Bedding 1973, Pape 1986, Pape and Arnaud 2001, Cerretti et al. 2014a) (Fig. 20). Analyses by Cerretti et al. (2014a) found the New Caledonian endemic genus *Rhinodonia* as sister to the remaining rhinophorids, and the Australasian genera *Axinia* and *Bixinia* as sister taxa nested within the *Stevenia* group. Our analyses still recovered *Bixinia* within the *Stevenia* group but notably diverged from Cerretti et al. (2014a) in retrieving *Rhinodonia* and *Axinia* as sister clades and part of an Oriental–Australasian radiation of the *Phyto* group. The differences between analyses stand likely by the double effect given by the new taxa included in the present version of the matrix and by the application of the implied weighting function implemented in TNT [k/(es+k), where k = concavity constant and es=extra step, i.e., as k increases, the function approximate the linear, equally weighted (“unweighted”) function (Goloboff 1993)]. Under implied weighting, the increase in fit (with differing strength depending upon the k-value) caused by the reduction in number of steps for a character is higher for characters with low homoplasy because each step represents a larger fraction of the total homoplasy for that character. In Fig. 20 one of these apomorphies, shared by the Australasian taxa except *Bixinia* (clade C), is the lack of a bend of vein M_1_ (45:1). The position of *Axinia* as a member of the *Phyto* group has recently been corroborated by molecular data (Cerretti et al. 2019). However, the affinities of *Bixinia* are more problematic, as both its separation from *Axinia* and its position within a Neotropical clade (I) appear unlikely, as ‘intuitively’ assessed by its relative similarity to other Australasian taxa (see below).

*Phyto* group and *Stevenia* group

The *Phyto* group (Fig. 20, clade B) was supported by four unambiguous character state changes [anal division of the first instar larva with longitudinal cuticular ridges in posteroventral position (95:1, global apomorphy) and a tongue-like projection in posterodorsal position (96:1, global apomorphy), cephaloskeleton of the first instar with longitudinal incision on parastomal bar (92:1, local apomorphy), and adult male with well-developed proclinate orbital setae (9:1, local apomorphy)]. The clade is composed exclusively of Old World elements. Remarkably, most of the characters whose derived condition supported the monophyly of clade B (*Phyto* group) are from the first instar larva, which is known for only three (i.e., *Paykullia*, *Melanophora*, and *Phyto*) of the 12 genera composing the group.

The *Stevenia* group (clade E) is nearly worldwide in distribution. Its monophyly was supported by four unambiguous character state changes [first instar larva with prolegs (93:1, global apomorphy), mandible provided with three or more teeth (99:0, global apomorphy), anal division with pair of vesicles in posteroventral position (97:1, local apomorphy), and adult male with bacilliform sclerite firmly fused to laterobasal margin of surstylus (65:1, local apomorphy)]. This clade was in turn divided into three subclades: F, H and I. Clade F is a relatively large assemblage supported by one global apomorphy [first instar larva with dorsoventrally flattened body (90:1)], and composed by Old World taxa widespread in the Afrotropical (*Maurhinophora* gen. nov., *Melanomyiodes*, *Queximyia*, *Stevenia* [in part]), Oriental (*Acompomintho* [in part], *Stevenia* [in part]), and Palaearctic (*Acompomintho* [in part], *Azaisia*, *Macrotarsina*, *Baniassa*, *Metoplisa*, *Oplisa*, *Rhinomorinia*, *Rhinophora*, *Tricogena*, *Tromodesia*, *Stevenia* [in part]) regions. Clade H was supported by two unambiguous character state changes of adult male [dorsal sclerite of acrophallus semicylindrical in shape (80:1, global apomorphy), and parafrontal plate with proclinate orbital setae (9:1, local apomorphy)] and includes only the endemic Afrotropical genus *Ventrops* (Cerretti & Pape, 2012). Finally, clade I is weakly supported by three local apomorphies [female without proclinate orbital setae (10:0), male surstylus fused to epandrium (66:1), and postgonite without anterior seta (69:1)], and is mostly composed of Neotropical taxa, with the notable exception of the Australian genus *Bixinia*, whose phylogenetic position seems unlikely and needs further study. *Ventrops* (i.e., clade H) was reconstructed as sister to clade I based on two local apomorphies [fronto-orbital plate with more than two proclinate orbital setae (11:1), and extensions of dorsal sclerite of distiphallus entirely fused mid-dorsally from each other into a single sclerotisation (71:1)]. The preimaginal stages and the natural history of *Ventrops* are unknown and more data are needed to clarify its phylogenetic position within Rhinophoridae.

The recent molecular-based phylogenetic reconstruction by Cerretti et al. (2019) recovered monophyly for both the *Phyto* group and the *Stevenia* group.

The *Aporeomyia* conundrum

The genus *Aporeomyia* was originally assigned to the family Tachinidae by Pape and Shima (1993), despite sharing with Rhinophoridae two local apomorphies, i.e., a tongue-like lower calypter (36:1) and anterior and posterior lappets of metathoracic spiracle subequal in size and directed outward (30:1). The original placement of this unusual taxon was based on the presence of a moderately convex subscutellum and a distiphallus characterised by an undivided extension of the dorsal sclerite, as in tachinids (Pape and Shima 1993). *Aporeomyia* is here reassigned to Rhinophoridae based on a reassessment of the homologies of the thorax and male terminalia. The subscutellum in *Aporeomyia* is moderately convex, but not bulging and fully sclerotised as in tachinids, which indicates a rhinophorid affinity. Notably, several rhinophorids also have a moderately convex subscutellum similar to that of *Aporeomyia*, in particular among species within various genera of the *Phyto* group, such as *Phyto*, *Comoromyia*, *Malayia*, *Rhinopeza*, and *Kinabalumyia* gen. nov. This character state is not restricted to this group: the Australasian genus *Bixinia* has a bulging subscutellum similar to that of tachinids, but its inclusion in Rhinophoridae is beyond any reasonable doubt (Kutty et al. 2019). As discussed by Pape and Shima (1993: 78, fig. 5), the extension of the dorsal sclerite of the distiphallus in *Aporeomyia antennalis* Pape & Shima is undivided, as typical for nearly all tachinids and a few other non-tachinid oestroids (see also Rognes 1997, Pape and Arnaud 2001). However, recent morphology-based cladistic analyses including a growing number of rhinophorid taxa indicate that an undivided extension of the dorsal sclerite of the distiphallus characterises several rhinophorid species belonging to different genera [e.g., *Rhinophora lepida* (Meigen), some *Bezzimyia*, *Shannoniella*, *Trypetidomima*, some *Melanophora*], thus confirming this trait as homoplasious within oestroids, and among rhinophorids in particular (Pape and Arnaud 2001, Cerretti and Pape 2012, Cerretti et al. 2014a, 2017). Moreover, the apparently undescribed species of *Aporeomyia* from Sabah included in our analysis is characterised by an extension of the dorsal sclerite of distiphallus that is longitudinally divided, even if shallowly, into two branches, suggesting that the two conditions can occur even in closely related species. We retrieved *Aporeomyia* nested within the *Phyto* group (clade B), as sister to the Oriental genus *Kinabalumyia* gen. nov. based on three unambiguous character state changes [first aristomere at least four times as long as wide (6:2, global apomorphy), wing vein CuA+CuP reaching wing margin (50:1, local apomorphy), and male with connection between tergite 6 and syntergosternite 7+8 fused (58:1, global apomorphy)]. The monophyly of *Aporeomyia* is based almost entirely on the strongly autapomorphic postpedicel with a tripartition (Fig. 6B) similar to that of the Neotropical *Bezzimyia lobata* Pape & Arnaud, but with sufficient differences to be considered a unique autapomorphy of *Aporeomyia*. In conclusion, we found strong evidence supporting *Aporeomyia* as a member of the Rhinophoridae as well as generic monophyly.

The position of *Kinabalumyia* gen. nov. within the *Phyto* group

The Oriental genus *Kinabalumyia* gen. nov. was supported by four unambiguous character state changes [distiphallus with a helmet-shaped, partly sclerotised envelope (81:1, global apomorphy), palpus reduced (21:1, local apomorphy), and male with: sternite 5 without posterior notch (53:1, local apomorphy), and connection between sternite 6 and syntergosternite 7+8 fused on right side (59:1, local apomorphy)] and it was reconstructed as sister to the Oriental genus *Aporeomyia* (see above). This clade was in turn retrieved as sister to the Australasian clade D (*Rhinodonia* + *Axinia*) based on two local apomorphies [base of wing vein R_4+5_ bare (43:1), and male cerci medially fused into a syncercus (61:1)]. We think that the helmet-shaped envelope of distiphallus is a strong autapomorphy supporting the erection of a new genus for the examined specimens. We have examined four specimens of *Kinabalumyia* gen. nov. from three localities scattered from Bali in Indonesia to Palawan in the Philippines, suggesting that this genus is widespread at least throughout the non-continental part of the Oriental Region, with further species probably awaiting discovery. Clade C (Fig. 20) was supported by two unambiguous character state changes [bend of vein M_1_ indistinct (45:1, global apomorphy) and metathoracic spiracular lappets absent (30:0, local apomorphy)] and it reconstructed the Australasian clade (clade D) arising from a grade composed of Australasian (*Rhinopeza*) and Oriental (*Aporeomyia*, *Kinabalumyia* gen. nov.) taxa. Despite being weakly supported by one local apomorphy [arista thickened at least on basal 3/4 (5:1)], clade C is in turn sister to the Oriental genus *Malayia*. If this reconstruction were accurate, the hypothesis of Cerretti et al. (2014a) about the origin of the Australasian rhinophorid fauna would need to be reconsidered. In fact, our phylogeny favours an Old World ancestor for most of the Australasian taxa, the only notable exception being *Bixinia* of Neotropical descent.

The *Stevenia* group and the position of *Maurhinophora* gen. nov., *Marshallicona* gen. nov. and *Neotarsina* gen. nov.

The monotypic genus *Maurhinophora* gen. nov. was erected to accommodate the new species *M. indoceanica* sp. nov. from Mauritius, based on a single female. The genus is supported in this work by six local apomorphies [facial plate and lower facial margin deeply sunken (13:1 and 14:1), palpus absent (21:2), lappets of metathoracic spiracle unequal in size, i.e., posterior one distinctly larger (30:2), wing vein R_1_ with a row of setulae along whole length (42:1), and base of wing vein R_4+5_ with a row of setae reaching at least crossvein r-m (44:1)] and was reconstructed as sister to the monotypic endemic Afrotropical genus *Queximyia*. *Maurhinophora* gen. nov. and *Queximyia* were part of the large clade F. The latter group was based on a global apomorphy of the first instar larva [first instar larva with slightly flattened body shape (90:1)], while an included subclade (clade G), also comprising *Maurhinophora* gen. nov. + *Queximyia*, was instead supported by one global apomorphy of the male terminalia [acrophallus distinctly tripartite, i.e., with three openings (79:1)]. This phylogenentic reconstruction suggests that the first instar and adult male of *Maurhinophora* gen. nov. (both unknown) might share these traits as well.

Two of the four newly described genera are Neotropical in distribution. During the last decades, the native New World rhinophorid fauna has increased remarkably from one genus and two species to the present six genera and 25 species (Pape and Arnaud 2001, Pape 2010, Cerretti et al. 2014a, Nihei and Andrade 2014, Nihei et al. 2016), and more are most likely awaiting discovery. Although based on a large array of local apomorphies, our analysis corroborated the non-monophyly of the genus *Bezzimyia*, as previously suggested by Cerretti and Pape (2012) and Cerretti et al. (2014a). The monotypic genus *Marshallicona* gen. nov. was supported by seven local apomorphies [arista microtrichia bottlebrush-like in male (2:1), male with proclinate orbital setae (9:1), base of wing vein R_4+5_ bare (43:1), abdominal tergites 3 and 4 without median marginal setae (51:1 and 52:1), abdominal tergite 6 bare in male (56:1), connection between surstylus and epandrium membranous (66:0), and phallic guide fused to pregonites (68:1)] and was reconstructed as sister to the clade *Trypetidomima* + *Shannoniella*.

The genus *Neotarsina* gen. nov. was erected for two new species from Peru (*N. andina* sp. nov.) and Trinidad and Tobago (*N. caraibica* sp. nov.). As described above for *Kinabalumyia* gen. nov., we suspect that *Neotarsina* gen. nov. is widespread in the Neotropics and also more diverse, not just including the presently described species. *Neotarsina* gen. nov. was reconstructed as sister to *Bezzimyia* (*B. barbarista* Pape & Arnaud + *B. busckii* Townsend) based on three unambiguous character changes [female with a groove between fronto-orbital plate and parafacial (8:1, global apomorphy), male fore tarsus laterally compressed (31:1, local apomorphy), and bacilliform sclerite articulated (i.e., not fused) to laterobasal margin of surstylus (65:0, local apomorphy)], and its monophyly relies on four unambiguous character changes [tibia of mid and hind legs laterally compressed and distinctly keeled dorsally (33:1, global apomorphy), palpus reduced (21:1, local apomorphy), male with connection between sternite 6 and syntergosternite 7+8 on right side (59:1, local apomorphy), and connection between dorsal sclerite of distiphallus and extensions of dorsal sclerite of distiphallus sclerotised (72:1, local apomorphy)].

## Catalogue

### Family Rhinophoridae Robineau-Desvoidy, 1863

RhinophoridaeRobineau-Desvoidy 1863: 3. Type genus: *Rhinophora*Robineau-Desvoidy 1830.

### Genus *Acompomintho* Villeneuve, 1927

***Acompomintho***Villeneuve 1927: 223. Type species: *Acompomintho lobata* Villeneuve, 1927, by monotypy.

*Wagneriopsis*Townsend 1927: 281. Type species: *Wagneriopsis formosensis* Townsend, 1927, by original designation.

[Acampomintho]: Townsend (1931: 383), Lopes (1938: 555), Herting (1961: 5); incorrect subsequent spelling of *Acompomintho* Villeneuve, 1927.

***caucasica*** (Villeneuve, 1908), stat. rev., comb. nov.

*Frauenfeldia caucasica*Villeneuve 1908: 287. Type locality: Russia, Krasnodar Krai, Tuapse. Holotype male not located [“coll. Schnabl”; “C. Tuapse”].

Remarks. Treated as unplaced to genus by Herting (1961: 34) but here resurrected as a valid species in the genus *Acompomintho*. Herting (1961) did not examine the holotype of *F. caucasica* and he claimed that the original description was inconclusive as to the generic placement of *caucasica*. In particular, Herting mentioned that despite similarities in colouration and habitus with *Tricogena rubricosa* (Meigen) [*Frauenfeldia* Egger is a junior synonym of *Tricogena*, see below], the original description of *F. caucasica* lacked any mention of robust setae on the lower half of the parafacial (characterising *Tricogena* and *Stevenia*, but not all described *Acompomintho*), or any information about other diagnostic characters. We have not been able to locate and examine the holotype of *caucasica*, but we consider the original description to match *Acompomintho* better than *Tricogena*.

Distribution. Palaearctic – Russia (North Caucasus).

***itoshimensis*** Kato & Tachi, 2016

*Acompomintho itoshimensis*Kato and Tachi 2016: 83. Type locality: Japan, Kyushu, Fukuoka, Shimasakurai. Holotype male in BLKU.

Distribution. Palaearctic – Japan (Kyushu).

***lobata*** Villeneuve, 1927

*Acompomintho lobata*Villeneuve 1927: 223. Type locality: Taiwan. Syntypes, male(s) and female(s), in DEI, NHMUK, USNM, possibly also elsewhere.

*Wagneriopsis formosensis*Townsend 1927: 282. Type locality: Taiwan: Hokuto, Anping, Kankau and Macuyama. Syntypes, male(s) and female(s), 1 male and 6 females in DEI, possibly also elsewhere.

Distribution. Palaearctic – North Korea, South Korea. Oriental – Japan (Ryukyu Is), Taiwan.

***sinensis*** (Villeneuve, 1936), stat. rev., comb. nov.

*Frauenfeldia sinensis*Villeneuve 1936: 7. Type locality: China, southern Gansu. Holotype female not located.

Remarks. Treated as unplaced to genus by Herting (1961: 34) but here resurrected as a valid species in the genus *Acompomintho*. The revised status of this nominal species is based on the recent discovery in Tajikistan of two female specimens (Table 4) belonging to the genus *Acompomintho*, which match the original description of *F. sinensis*.

Distribution. Palaearctic – China (Gansu), Tajikistan (Gorno-Badachšan, Rŭshan) [**new record**].

### Genus *Apomorphyto* Cerretti, Lo Giudice & Pape, 2014

***Apomorphyto***Cerretti et al. 2014a: 675. Type species: *Apomorphyto inbio* Cerretti, Lo Giudice & Pape, 2014, by original designation.

***inbio*** Cerretti, Lo Giudice & Pape, 2014

*Apomorphyto inbio*Cerretti et al. 2014a: 676. Type locality: Costa Rica, Guanacaste, Estación Las Pailas, Parc Nacional Rincón de la Vieja. Holotype male in INBio.

Distribution. Neotropical – Costa Rica, Nicaragua.

### Genus *Aporeomyia* Pape & Shima, 1993

***Aporeomyia***Pape and Shima 1993: 77. Type species: *Aporeomyia antennalis* Pape & Shima, 1993, by original designation.

***antennalis*** Pape & Shima, 1993

*Aporeomyia antennalis*Pape and Shima 1993: 77. Type locality: Philippines, Mindanao, Mt. Apo, Lake Agco. Holotype male in BLKU.

Distribution. Oriental – Philippines (Mindanao).

**Undescribed sp.**: Malaysia (Sabah) (CNC, NHMD).

### Genus *Axinia* Colless, 1994

***Axinia***Colless 1994a: 484. Type species: *Axinia arenaria* Colless, 1994, by original designation.

Remarks. There are two spellings of this genus-group name in Colless (1994): *Axinia* on page 484 plus another 90 occurrences, and *Axinis* on page 502, used in the combination “*Axinis cornuta*”. We consider the multiple use of the spelling “*A. cornuta*” in contexts where the “*A.*” is evidently an abbreviation for “*Axinia*”, as “clear evidence of an inadvertent error” (Code, Article 32.5.1), and *Axinis* must accordingly be corrected to *Axinia*.

*Dixicera*Colless 1994a: 508 (as subgenus of *Axinia* Colless, 1994). Type species: *Axinia carnei* Colless, 1994, by original designation.

*Barrinea*Colless 1994a: 511. Type species: *Barrinea disjuncta* Colless, 1994, by original designation.

*Ismaya*Colless 1994a: 512. Type species: *Ismaya miranda* Colless, 1994, by original designation. [Junior homonym of *Ismaya* Bouček, 1988 (Hymenoptera: Chalcidoidea).]

*Chirops*Colless 1994a: 516. Type species: *Chirops arcana* Colless, 1994, by original designation.

*Johnismaya*Colless 1994b: 380. New name for *Ismaya* Colless, 1994.

[Axinis]: Colless (1994a: 502); incorrect original spelling of *Axinia* Colless [*teste* this work].

***arcana*** (Colless, 1994)

*Chirops arcana*Colless 1994a: 517. Type locality: Papua New Guinea, E end Saruwaged Range, 20 km SSW Kabwum, 2550 m. Holotype male in BPBM.

Distribution. Australasian – Papua New Guinea (PNG).

***arenaria*** Colless, 1994

Axinia (Axinia) arenariaColless 1994a: 486. Type locality: Australia, Western Australia, 37 mi N Ajana. Holotype male in ANIC.

Distribution. Australasian – Australia (Northern Territory, South Australia, Western Australia).

***austrina*** Colless, 1994

Axinia (Axinia) austrinaColless 1994a: 496. Type locality: Australia, Australian Capital Territory, Blundells Creek. Holotype male in ANIC.

Distribution. Australasian – Australia (ACT, New South Wales).

***bicolor*** Colless, 1994

Axinia (Axinia) bicolorColless 1994a: 505. Type locality: Australia, Queensland, Lamington National Park, O’Reilly’s. Holotype male in ANIC.

Distribution. Australasian – Australia (Queensland).

***brevicentrum*** Colless, 1994

Axinia (Axinia) brevicentrumColless 1994a: 503. Type locality: Australia, Western Australia, 37 km SW Mt Ragged. Holotype male in ANIC.

Distribution. Australasian – Australian (South Australia, Victoria, Western Australia).

***cantrelli*** Colless, 1994

Axinia (Axinia) cantrelliColless 1994a: 489. Type locality: Australia, Queensland, Mt Tamborine. Holotype male in QDPC.

Distribution. Australasian – Australia (New South Wales, Queensland).

***carnei*** Colless, 1994

Axinia (Dixicera) carneiColless 1994a: 508. Type locality: Australia, Western Australia, 8 mi ENE Millstream. Holotype male in ANIC.

Distribution. Australasian – Australia (Western Australia).

***cornuta*** Colless, 1994

Axinia (Axinia) cornutaColless 1994a: 502. Type locality: Australia, Australian Capital Territory, Blundells Creek. Holotype male in ANIC.

Distribution. Australasian – Australia (ACT, Tasmania, Victoria).

***disjuncta*** (Colless, 1994)

*Barrinea disjuncta*Colless 1994a: 511. Type locality: Australia, Queensland, Lake Barrine. Holotype male in ANIC.

Distribution. Australasian – Australia (Queensland).

***gressitti*** Colless, 1994

Axinia (Axinia) gressittiColless 1994a: 501. Type locality: Papua New Guinea, Morobe Province, Mt Missim, 1600 m. Holotype male in BPBM.

Distribution. Australasian – Papua New Guinea (Morobe).

***lucaris*** Colless, 1994

Axinia (Axinia) lucarisColless 1994a: 492. Type locality: Australia, Queensland, 13 km NE by N Yungaburra, Danbulla Forest Reserve. Holotype male in ANIC.

Distribution. Australasian – Australia (New South Wales, Queensland).

***minuta*** Colless, 1994

Axinia (Dixicera) minutaColless 1994a: 509. Type locality: Australia, Western Australia, Crowea near Busselton. Holotype male in ANIC.

Distribution. Australasian – Australia (Western Australia).

***miranda*** (Colless, 1994)

*Ismaya miranda*Colless 1994a: 514. Type locality: Papua New Guinea, Northern Province, Mt Scratchley, 3500 m. Holotype male in ANIC.

Distribution. Australasian – Papua New Guinea (Northern Province).

***mutabilis*** Colless, 1994

Axinia (Axinia) mutabilisColless 1994a: 499. Type locality: Australia, Queensland, Lamington National Park, O’Reilly’s. Holotype male in ANIC.

Distribution. Australasian – Australia (Queensland).

***naumanni*** Colless, 1994

Axinia (Axinia) naumanniColless 1994a: 498. Type locality: Australia, Tasmania, 4 km E Rosebery. Holotype male in ANIC.

Distribution. Australasian – Australia (Tasmania).

***zentae*** Colless, 1994

Axinia (Axinia) zentaeColless 1994a: 495. Type locality: Australia, Australian Capital Territory, Blundells Creek. Holotype male in ANIC.

Distribution. Australasian – Australia (ACT, New South Wales).

### Genus *Azaisia* Villeneuve, 1939

***Azaisia***Villeneuve 1939: 350. Type species: *Azaisia setitarsis* Villeneuve, 1939, by original designation.

*Azaisiella*Villeneuve 1939: 351 (as subgenus of *Azaisia* Villeneuve, 1939). Type species: *Azaisiella obscura* Villeneuve, 1939, by original designation.

***obscura*** Villeneuve, 1939

Azaisia (Azaisiella) obscuraVilleneuve 1939: 352. Type locality: Madeira. Holotype female in IRSNB.

Distribution. Palaearctic – Azores?, Madeira.

***setitarsis*** Villeneuve, 1939

*Azaisia setitarsis*Villeneuve 1939: 351. Type locality: Madeira. Holotype female in IRSNB.

Distribution. Palaearctic – Madeira.

### Genus *Baniassa* Kugler, 1978

***Baniassa***Kugler 1978: 73. Type species: *Baniassa fascipennis* Kugler, 1978, by orginal designation.

***fascipennis*** Kugler, 1978

*Baniassa fascipennis*Kugler 1978: 74. Type locality: Israel, Mt Carmel. Holotype male in TAU.

Distribution. Palaearctic – Israel.

***fenestrata*** Zeegers, 2008

*Baniassa fenestrata*Zeegers 2008: 733, 734. Type locality: United Arab Emirates, Fujairah. Holotype male in NBCL.

Distribution. Palaearctic/Afrotropical – Oman, United Arab Emirates.

***paucipila*** Pape, 1985

*Baniassa paucipila*Pape 1985: 209. Type locality: Iraq, Kurdistan, Arbil. Holotype male in USNM (unique specimen identifier: USNMENT01519730).

Distribution. Palaearctic – Iraq.

### Genus *Bezzimyia* Townsend, 1919

***Bezzimyia***Townsend 1919a: 591. Type species: *Bezzimyia busckii* Townsend, 1919, by original designation.

*Lutzomyia*Curran 1934: 387. Type species: *Lutzomyia americana* Curran, 1934, by monotypy. [Preoccupied by *Lutzomyia* França, 1927 (Diptera: Psychodidae).]

*Pseudolutzomyia*Rapp 1945: 278. New name for *Lutzomyia* Curran, 1934.

[Pseudolutozmyia]: Rapp (1945: 278); incorrect original spelling of *Pseudolutzomyia* [*teste*Pape and Arnaud (2001: 261)].

**Group A**

***bisecta*** Pape & Arnaud, 2001

*Bezzimyia bisecta*Pape and Arnaud 2001: 260, 265. Type locality: Costa Rica, San José, Braulio Carillo National Park, 10°07'N, 83°58'W, 1000 m. Holotype male in USNM (unique specimen identifier: USNMENT01519747).

Distribution. Neotropical – Costa Rica.

***hansoni*** Pape & Arnaud, 2001

*Bezzimyia hansoni*Pape and Arnaud 2001: 260, 273. Type locality: Costa Rica, Cartago, La Cangreja, 1950 m. Holotype male in USNM (unique specimen identifier: USNMENT01519735).

Distribution. Neotropical – Costa Rica.

***pittieri*** Pape & Arnaud, 2001

*Bezzimyia pittieri*Pape and Arnaud 2001: 261, 279. Type locality: Venezuela, Aragua, Henri Pittier National Park, Pico Periquito, 1680 m. Holotype male in MIZA.

Distribution. Neotropical – Venezuela.

***ramicornis*** Pape & Arnaud, 2001

*Bezzimyia ramicornis*Pape and Arnaud 2001: 260, 281. Type locality: Ecuador, Pichincha, nr Tinalandia [hotel 16 km SE Santo Domingo], 1150 m. Holotype male in CNC.

Distribution. Neotropical – Ecuador.

***yepezi*** Pape & Arnaud, 2001

*Bezzimyia yepezi*Pape and Arnaud 2001: 261, 286. Type locality: Venezuela, Aragua, Rancho Grande, 1100 m. Holotype male in MIZA.

Distribution. Neotropical – Venezuela.

**Group B**

***americana*** (Curran, 1934)

*Lutzomyia americana*Curran 1934: 387. Type locality: USA, Arizona, Cobabi Mts., Sta. Cruz Village, 3100 ft. Lectotype male (by designation of Sabrosky 1963: 14) in AMNH.

[Lutzomyialatifrons]: Curran (1934: 396, 398); incorrect original spelling of *americana* Curran, 1934 [*teste*Sabrosky (1963: 14)].

Distribution. Nearctic – USA (Arizona).

***barbarista*** Pape & Arnaud, 2001

*Bezzimyia barbarista*Pape and Arnaud 2001: 260, 263. Type locality: Costa Rica, Cartago, La Cangreja, 1950 m. Holotype male in USNM (unique specimen identifier: USNMENT01519746).

Distribution. Neotropical – Belize, Costa Rica.

***bulbosa*** Pape & Arnaud, 2001

*Bezzimyia bulbosa*Pape and Arnaud 2001: 260, 267. Type locality: Mexico, Veracruz, Fortín de Las Flores, Sumidero, Planta de la Serveceria. Holotype male in USNM (unique specimen identifier: USNMENT01519748).

Distribution. Neotropical – Mexico (Vera Cruz).

***busckii*** Townsend, 1919

*Bezzimyia busckii*Townsend 1919a: 592. Type locality: Panama, Trinidad River [“Trinidad Rio”]. Holotype male in USNM (unique specimen identifier: USNMENT01519740).

Distribution. Nearctic – USA (Florida, Texas). Neotropical – Panama.

***floridensis*** Pape & Arnaud, 2001

*Bezzimyia floridensis*Pape and Arnaud 2001: 260, 271. Type locality: USA, Florida, Jackson County, Florida Caverns State Park. Holotype male in USNM (unique specimen identifier: USNMENT01519743).

Distribution. Nearctic – USA (Florida).

***jamaica*** Pape & Arnaud, 2001

*Bezzimyia jamaica*Pape and Arnaud 2001: 261, 275. Type locality: Jamaica, Hardwar Gap, 4000 ft. Holotype female in CNC.

Distribution. Neotropical – Jamaica.

***lapidicina*** Pape & Arnaud, 2001

*Bezzimyia lapidicina*Pape and Arnaud 2001: 261, 275. Type locality: Costa Rica, Puntarenas, Las Alturas, quarry (tajo) near Las Alturas Station. Holotype male in USNM (unique specimen identifier: USNMENT01519736, USNMENT01519737 [wing slide]).

Distribution. Neotropical – Costa Rica.

***orestes*** Pape & Arnaud, 2001

*Bezzimyia orestes*Pape and Arnaud 2001: 261, 277. Type locality: Mexico, Chiapas, 3 mi NE San Cristobal. Holotype male in CNC.

Distribution. Neotropical – Mexico (Chiapas).

***platina*** Pape & Arnaud, 2001

*Bezzimyia platina*Pape and Arnaud 2001: 261, 280. Type locality: Mexico, Chiapas, 20 mi N Bochil, Yerba Buena, 6500 ft. Holotype male in CNC.

Distribution. Neotropical – Mexico (Chiapas).

***setifax*** Pape & Arnaud, 2001

*Bezzimyia setifax*Pape and Arnaud 2001: 261, 282. Type locality: Costa Rica, Guanacaste, Estación Maritza, 600 m. Holotype male in INBio.

Distribution. Neotropical – Costa Rica.

***sternothrix*** Pape & Arnaud, 2001

*Bezzimyia sternothrix*Pape and Arnaud 2001: 260, 285. Type locality: Costa Rica, Guanacaste, Tierras Morenas, 685 m. Holotype male in INBio.

Distribution. Neotropical – Costa Rica.

***thompsonorum*** Pape & Arnaud, 2001

*Bezzimyia thompsonorum*Pape and Arnaud 2001: 261, 286. Type locality: USA, Georgia, Liberty County, St. Catherines Island. Holotype male in AMNH.

Distribution. Nearctic – Mexico (San Luis Potosí), USA (Georgia).

### Genus *Bixinia* Cerretti, Lo Giudice & Pape, 2014

***Bixinia***Cerretti et al. 2014a: 668. Type species: *Bixinia winkleri* Cerretti, Lo Giudice & Pape, 2014, by original designation.

***collessi*** Cerretti, Lo Giudice & Pape, 2014

*Bixinia collessi*Cerretti et al. 2014a: 668. Type locality: Australia, Western Australia, 37 km SW of Mt Ragged. Holotype male in ANIC.

Distribution. Australasian – Australia (Western Australia).

***solitaria*** Cerretti, Lo Giudice & Pape, 2014

*Bixinia solitaria*Cerretti et al. 2014a: 671. Type locality: Australia, New South Wales, Mt Clyde. Holotype male in ANIC.

Distribution. Australasian – Australia (New South Wales).

***spei*** Cerretti, Lo Giudice & Pape, 2014

*Bixinia spei*Cerretti et al. 2014a: 673. Type locality: Australia, Western Australia, Thomas River estuary. Holotype male in ANIC.

Distribution. Australasian – Australia (Western Australia).

***variabilis*** Cerretti, Lo Giudice & Pape, 2014

*Bixinia variablis*Cerretti et al. 2014a: 673. Type locality: Australia, New South Wales, Congo, 35.58S, 150.09E. Holotype male in ANIC.

Distribution. Australasian – Australia (Western Australia, Tasmania).

***winkleri*** Cerretti, Lo Giudice & Pape, 2014

*Bixinia winkleri*Cerretti et al. 2014a: 674. Type locality: Australia, Queensland, Wongabel State Forest, nr Atherton, 17.20S 145.31E. Holotype male in ANIC.

Distribution. Australasian – Australia (Queensland).

### Genus *Comoromyia* Crosskey, 1977

***Comoromyia***Crosskey 1977: 46. Type species: *Comoromyia griseithorax* Crosskey, 1977, by original designation.

***griseithorax*** Crosskey, 1977

*Comoromyia griseithorax*Crosskey 1977: 47. Type locality: Comoro Is, Anjouan, Col de Moya, 850 m. Holotype female in MNHN.

Distribution. Afrotropical – Comoro Is.

**Undescribed sp. 1**: Madagascar, Andringitra (MNHN) (Cerretti and Pape 2012, Cerretti et al. 2014a).

**Undescribed sp. 2**: Madagascar, Andringitra (MNHN) (Cerretti and Pape 2012, Cerretti et al. 2014a).

**Undescribed sp. 3**: Madagascar (TAU) (Cerretti et al., unpubl.).

### Genus *Kinabalumyia* Cerretti & Pape, gen. nov.

***Kinabalumyia*** Cerretti & Pape, gen. nov. Type species: *Kinabalumyia pinax* Cerretti & Pape, sp. nov., by present designation.

***pinax*** Cerretti & Pape, sp. nov.

*Kinabalumyia pinax* Cerretti & Pape, sp. nov. Type locality: Malaysia, Sabah, Mount Kinabalu. Holotype male in NHMUK.

Distribution. Oriental – Malaysia (Sabah).

**Unidentified sp. 1**: Philippines (Palawan) (NHMD).

**Undescribed sp. 2**: Indonesia (Bali) (photographic material, available from www.diptera.info).

### Genus *Macrotarsina* Schiner, 1857

*Zelleria*Egger 1856: 385. Type species: *Zelleria longimana* Egger, 1856, by monotypy. [Junior homonym of *Zelleria* Stainton, 1849 (Lepidoptera: Yponomeutidae).]

***Macrotarsina***Schiner 1857: 4. New name for *Zelleria* Egger, 1856.

*Braueria*Schiner 1861a: 142. New name for *Zelleria* Egger, 1856.

[Microtarsina]: Verves and Khrokalo (2006: 9); incorrect subsequent spelling of *Macrotarsina* Schiner, 1857.

***longimana*** (Egger, 1856)

*Zelleria longimana*Egger 1856: 385. Type locality: Italy, Trieste [“bei Triest ... am Ufer”]. Syntypes, male(s) and female(s), in NHMW.

*Macrotarsina zelleri*Schiner 1857: 4. New name for *Zelleria longimana* Egger, 1856.

Distribution. Palaearctic – Croatia, Cyprus, Gibraltar, Italy (mainland, Sicily [**new record**]), Malta.

### Genus *Malayia* Malloch, 1926

***Malayia***Malloch 1926: 510. Type species: *Malayia fuscinervis* Malloch, 1926, by original designation.

***fuscinervis*** Malloch, 1926

*Malayia fuscinervis*Malloch 1926: 511. Type locality: Malaysia, West Malaysia, Pahang, Cameron Highlands. Holotype female in NHMUK.

Distribution. Oriental – Malaysia (West Malaysia).

***indica*** Lo Giudice, Pape & Cerretti, 2016

*Malayia indica*Lo Giudice et al. 2016: 62. Type locality: India, Tamil Nadu, Kodaikanal. Holotype female in NHMUK.

Distribution. Oriental – India (Tamil Nadu).

***nigripennis*** Malloch, 1927

*Malayia nigripennis*Malloch 1927: 416. Type locality: Malaysia, West Malaysia, Selangor, Bukit Kutu. Holotype female in NHMUK.

Distribution. Oriental – Indonesia (Sumatra), Malaysia (West Malaysia).

### Genus *Marshallicona* Cerretti & Pape, gen. nov.

***Marshallicona*** Cerretti & Pape, gen. nov. Type species: *Marshallicona quitu* Cerretti & Pape, sp. nov., by present designation.

***quitu*** Cerretti & Pape, sp. nov.

*Marshallicona quitu* Cerretti & Pape, sp. nov. Type locality: Ecuador, Mindo-Bellavista, Bellavista cloud forest. Holotype male in PUCE.

Distribution. Neotropical – Ecuador.

### Genus *Maurhinophora* Cerretti & Pape, gen. nov.

***Maurhinophora*** Cerretti & Pape, gen. nov. Type species: *Maurhinophora indoceanica* Cerretti & Pape, sp. nov., by present designation.

***indoceanica*** Cerretti & Pape, sp. nov.

*Maurhinophora indoceanica* Cerretti & Pape, sp. nov. Type locality: Mauritius, Corps de la Garde. Holotype female in NHMUK.

Distribution. Afrotropical – Mauritius.

### Genus *Melanomyoides* Crosskey, 1977

***Melanomyoides***Crosskey 1977: 23. Type species: *Chaetostevenia capensis* Zumpt, 1959, by original designation.

***capensis*** (Zumpt, 1959)

*Chaetostevenia capensis*Zumpt 1959: 433. Type locality: South Africa, Western Cape, Cape Peninsula, Hout Bay, Skoorstenskop. Lectotype male (by fixation of Crosskey 1977: 25) in MZLU.

Distribution. Afrotropical – South Africa (Western Cape).

### Genus *Melanophora* Meigen, 1803

***Melanophora***Meigen 1803: 279. Type species: *Musca grossificationis* Linnaeus, 1758 [= *Musca roralis* Linnaeus, 1758], by monotypy.

[Milanophora]: Fabricius (1805: 304); incorrect subsequent spelling of *Melanophora* Meigen, 1803.

[Melanophera]: Fischer von Waldheim (1813: 350); incorrect subsequent spelling of *Melanophora* Meigen, 1803.

*Illigeria*Robineau-Desvoidy 1830: 273. Type species: *Illigeria atra* Robineau-Desvoidy, 1830 [= *Musca roralis* Linnaeus, 1758], by designation of Townsend (1916: 7).

*Sylvanus*Gistel 1848: xi. New name for *Melanophora* Meigen, 1803.

[Melanosphora]: Shimer (1871: 219); incorrect subsequent spelling of *Melanophora* Meigen, 1803.

*Bequaertiana*Curran 1929: 14. Type species: *Bequaertiana argyriventris* Curran, 1929, by original designation.

***argyriventris*** (Curran, 1929)

*Bequaertiana argyriventris*Curran 1929: 15. Type locality: Liberia, Du River, “Camp No. 3”. Holotype male in AMNH.

Distribution. Afrotropical – Liberia.

***asetosa*** Kugler, 1978

*Melanophora asetosa*Kugler 1978: 78. Type locality: Israel, Negev, Yeruham. Holotype female in TAU.

Distribution. Palaearctic – Israel.

***basilewskyi*** (Peris, 1957)

*Bequaertiana basilewskyi*Peris 1957: 136. Type locality: Rwanda, Ruhengeri, 1900 m. Holotype male in MRAC.

Distribution. Afrotropical – Kenya, Rwanda, Uganda, R.D. Congo.

***chia*** Cerretti & Pape, 2009

*Melanophora chia*Cerretti and Pape 2009: 555. Type locality: Italy, Sardinia, Torre di Chia, Dòmus de Maria. Holotype male in MZUR.

Distribution. Palaearctic – Italy (Sardinia).

***roralis*** (Linnaeus, 1758)

*Musca roralis*Linnaeus 1758: 597. Type locality: Europe. Lectotype female (by fixation of Crosskey 1977: 51) in LSUK.

*Musca grossificationis*Linnaeus 1758: 599. Type locality: Europe. Lectotype female (by fixation of Crosskey 1977: 51) in LSUK.

*Musca interventum*Harris 1780: 144. Type locality: England. Type(s), unspecified sex, lost [*teste*Crosskey (1977)].

*Tachina plumigera*Wiedemann 1830: 342. Type locality: Egypt [“Egypten”]. Syntypes, unspecified number and sex, in ZMHB and ZMUH.

*Melanophora violacea*Robineau-Desvoidy 1830: 272. Type locality: France, Paris. Type(s), female, not located.

*Melanophora atra*Robineau-Desvoidy 1830: 272. Type locality: France, Paris. Type(s), female, not located.

*Melanophora festiva*Robineau-Desvoidy 1830: 272. France, Angers. Type(s), female, not located.

*Melanophora distincta*Robineau-Desvoidy 1830: 273. Type locality: France, Paris. Type(s), female, not located.

*Illigeria atra*Robineau-Desvoidy 1830: 274. Type locality: France, Paris. Type(s), male, not located.

*Illigeria minor*Robineau-Desvoidy 1830: 274. Type locality: France, Yonne, St Sauveur. Holotype, male, not located.

*Melanophora nigerrima*Macquart 1834: 232. New name for *Illigeria atra* Robineau-Desvoidy, 1830.

*Melanophora americana*Macquart 1844: 72. Type locality: Brazil or Chile [“Du Brésil ou du Chili”]. Type(s), unspecified sex, not located [“M. Gaudichaud Muséum”].

*Tachina interlapsa*Walker 1853: 37. Type locality: England. Type(s), unspecified sex, lost [*teste*Crosskey (1974)].

*Melanophora appendiculata*Macquart 1855b: 202. Type locality: Italy, Sicily. Type(s), female, not located.

Remarks. Probably a junior synonym of *Melanophora roralis* (Linnaeus, 1758) according to Herting (1993: 117), but no diagnostic character states can be retrieved from the original description.

*Illigeria brasiliensis*Robineau-Desvoidy 1863: 1138. Type locality: Brazil. Type(s), female, not located, possibly in MNHN.

*Melanophora nitidiventris*Curran 1928: 41. Type locality: Jamaica, Cinchona. Holotype male in AMNH.

Distribution. Afrotropical – Cape Verde (Santo Antão, Brava, St Vincent). Nearctic [introduced] – Canada (Ontario) [**new record**], USA (California [**new record**], Florida, Kansas, Louisiana, Maryland, Massachusetts, Michigan, New Hampshire, Ohio, Pennsylvania). Neotropical [introduced] – Argentina (Buenos Aires, Mendoza), Brazil (no further data), British Virgin Islands (Charlotte Amalie) [**new record**], Chile (no further data), Jamaica, Uruguay. Palaearctic – Algeria, Azores, Belgium, Croatia, Czech Republic (Bohemia, Moravia), Denmark, Egypt, France (mainland, Corsica), Germany, Hungary, Ireland, Italy (mainland, Sardinia, Sicily), Japan [introduced?], Lichtenstein, Malta, Netherlands, Norway, Poland, Slovakia, Spain (mainland, Balearic Islands), Sweden, Switzerland, Ukraine (Cherkasy, Chernigiv, Crimea, Ivano-Frankivs’k, Kyiv, Poltava, Zaporizzhya, Zhytomyr), United Kingdom (England), Yugoslavia.

### Genus *Metoplisa* Kugler, 1978

***Metoplisa***Kugler 1978: 80. Type species: *Metoplisa carbonaria* Kugler, 1978, by original designation.

***carbonaria*** Kugler, 1978

*Metoplisa carbonaria*Kugler 1978: 81. Type locality: Israel, Ein Tureiba. Holotype male in TAU.

Distribution. Palaearctic – Israel.

### Genus *Neotarsina* Cerretti & Pape, gen. nov.

***Neotarsina*** Cerretti & Pape, gen. nov. Type species: *Neotarsina caraibica* Cerretti & Pape, sp. nov., by present designation.

***andina*** Cerretti & Pape, sp. nov.

*Neotarsina andina* Cerretti & Pape, sp. nov. Type locality: Peru, Raymondi. Holotype male in CULSP.

Distribution. Neotropical – Peru.

***caraibica*** Cerretti & Pape, sp. nov.

*Neotarsina caraibica* Cerretti & Pape, sp. nov. Type locality: Trinidad and Tobago, Trinidad, Curepe. Holotype male in NHMUK.

Distribution. Neotropical – Trinidad and Tobago (Trinidad).

### Genus *Oplisa* Rondani, 1862

***Oplisa***Rondani 1862: 155. Type species: *Oplisa mendica* Rondani, 1862 [= *Morinia tergestina* Schiner, 1862], by monotypy.

*Hoplisa*Brauer and Bergenstamm 1889: 124. Unjustified emendation of *Oplisa* Rondani, 1862.

*Melanomelia*Strobl 1899: 215. Type species: *Melanomelia aterrima* Strobl, 1899, by monotypy.

*Anoplisa*Herting 1961: 10 (as subgenus of *Oplisa* Rondani, 1862). Type species: *Hoplisa oldenbergi* Herting, 1961, by monotypy.

***aterrima*** (Strobl, 1899)

*Melanomelia aterrima*Strobl 1899: 215. Type locality: Spain, Algericas and Spain, Sierra Nevada. Syntypes, 1 male and 2 females (Algeciras); 2 males and 1 female (Sierra Nevada), possibly in NMBA.

Distribution. Palaearctic – Gibraltar, Italy (Sicily [**new record**]), Morocco, Portugal [**new record**], Spain, Tunisia.

***caesia*** (Villeneuve, 1911)

*Hoplisa caesia*Villeneuve 1911: 125. Type locality: France, Corsica, Vico. Holotype male not located [“dans la collect. Schnabl”].

Distribution. Palaearctic – France (Corsica).

***grandiloba*** Kugler, 1978

*Oplisa grandiloba*Kugler 1978: 82. Type locality: Israel, Ramot Naftali. Holotype male in TAU.

Distribution. Palaearctic – Israel.

***hertingi*** Zeegers, 2011

*Oplisa hertingi*Zeegers 2011: 315. Type locality: Turkey, Province Hakkari, Habur Deresi-Tal, S ‘Beylisebap’. Holotype male in SMNS.

Distribution. Palaearctic – Turkey.

***japonica*** Pape & Kurahashi, 1994

*Oplisa japonica*Pape and Kurahashi 1994: 477. Type locality: Japan, Kyushu, Fukuoka, Aburayama. Holotype male in BLKU.

Distribution. Palaearctic – Japan (Honshu, Kyushu).

***nudiseta*** Zeegers, 2011

*Oplisa nudiseta*Zeegers 2011: 316. Type locality: Turkey, E Antalya. Holotype female in SMNS.

Distribution. Palaearctic – Turkey.

***oldenbergi*** (Herting, 1961)

*Hoplisa oldenbergi*Herting 1961: 11. Type locality: Romania, Mehadia. Holotype male in DEI.

Distribution. Palaearctic – Czech Republic (Bohemia), Romania, Poland, Russia (North Caucasus), Turkey, Ukraine (Crimea).

***pollinosa*** Kugler, 1978

Oplisa (Anoplisa) pollinosaKugler 1978: 84. Type locality: Israel, Carmel. Holotype male in TAU.

Distribution. Palaearctic – Israel, North Korea (? tentative record from Draber-Mońko 2007).

***tergestina*** (Schiner, 1861)

*Morinia tergestina*Schiner 1861b: 552. Type locality: Italy, Trieste. Type(s), unspecified sex, not located, possibly in NHMW.

*Oplisa mendica*Rondani 1862: 155. Type locality: Italy, probably Parma. Lectotype male (by designation of Pape 1989: 357) in MZF.

Distribution. Palaearctic – Czech Republic (Bohemia, Moravia), Germany, Hungary, Italy (mainland), Poland, Romania, Slovakia, Switzerland, Ukraine (Cherkasy).

### Genus *Parazamimus* Verbeke, 1962

***Parazamimus***Verbeke 1962: 164. Type species: *Parazamimus congolensis* Verbeke, 1962, by original designation.

***congolensis*** Verbeke, 1962

*Parazamimus congolensis*Verbeke 1962: 164. Type locality: D.R. Congo, Kivu, Goma. Holotype male in IRSNB.

Distribution. Afrotropical – Burundi, D.R. Congo.

### Genus *Paykullia* Robineau-Desvoidy, 1830

***Paykullia***Robineau-Desvoidy 1830: 270. Type species: *Paykullia rubricornis* Robineau-Desvoidy, 1830 [= *Ocyptera maculata* Fallén, 1815], by designation of Coquillett (1910: 585).

[Paykulia]: Schiner (1868: 293); incorrect subsequent spelling of *Paykullia* Robineau-Desvoidy, 1830.

*Chaetostevenia*Brauer 1895: 604. Type species: *Stevenia partenopea* Rondani, 1861, by original designation.

*Parafeburia*Townsend 1933: 446. Type species: *Ocyptera maculata* Fallén, 1815, by original designation.

*Euplesina*Wainwright 1933: 255. Type species: *Ocyptera maculata* Fallén, 1815, by original designation.

***braueri*** (Strobl, 1895)

Stevenia (Catharosia) braueriStrobl 1895: 246. Type locality: Slovenia, Zidani Most [“Auf Bergwiesen bei Steinbrück”]. Syntypes, 1 male and 1 female, not located (possibly in NHMW).

Distribution. Palaearctic – Albania, Czech Republic (Bohemia), Croatia [**new record**], Slovenia.

***brevicornis*** (Zetterstedt, 1844)

*Leucostoma brevicornis*Zetterstedt 1844: 1233. Type locality: Sweden: Småland and Omberg, Östergötland. Syntypes, 1 male and 1 female, in MZLU.

*Euplesina ringdahli*Villeneuve 1934a: 184. Type locality: Sweden, Skåne, Skäralid. Syntypes, 1 male and 1 female, in MZLU.

Distribution. Palaearctic – Norway, Sweden, Switzerland.

***carmela*** (Peris, 1963)

*Chaetostevenia carmela*Peris 1963: 606. Type locality: Morocco, Tanger. Holotype male in MNCN.

Distribution. Palaearctic – Morocco.

***insularis*** (Villeneuve, 1911)

*Stevenia insularis*Villeneuve 1911: 122. Type locality: France, Corsica. Syntypes, 3 males and 3 females [“Collection Becker ... coll. Kuntze ... coll. Schnabl”], 1 syntype female in IRSNB.

Distribution. Palaearctic – France (Corsica).

***kugleri*** (Herting, 1961)

*Chaetostevenia kugleri*Herting 1961: 31. Type locality: Israel, Yagur. Holotype male in TAU.

Distribution. Palaearctic – Cyprus, Israel.

***maculata*** (Fallén, 1815)

*Ocyptera maculata*Fallén 1815: 237. Type locality: Sweden: Skåne, Esperöd and Västergötland. Syntypes, females, in NHRS and/or MZLU.

*Paykullia rubricornis*Robineau-Desvoidy 1830: 270. Type locality: France, Paris. Type(s), unspecified sex, not located.

*Paykullia riparia*Robineau-Desvoidy 1830: 271. Type locality: France. Type(s), female, not located.

*Leucostoma ruficornis*Macquart 1855b: 192. Type locality: France, Lorraine. Type(s), female, not located.

Distribution. Palaearctic – Austria, Czech Republic (Bohemia, Moravia), Denmark, France (mainland), Germany, Hungary, Ireland, Italy (mainland), Netherlands, Norway, Poland, Russia (North Caucasus), Slovakia, Spain [**new record**], Sweden, Switzerland, Ukraine (Zakarpattya), United Kingdom (England, Scotland).

***nubilipennis*** (Loew, 1847)

*Plesina nubilipennis*Loew 1847: 261. Type locality: Italy, nr. Napoli. Syntypes, males, in ZMHB.

*Stevenia parmensis*Rondani 1861: 145. Type locality: Italy, Parma. Lectotype male (by designation of Pape 1989: 357) in MZF.

*Stevenia sicula*Rondani 1868: 47. Type locality: Italy, fields near Palermo [“agro Panormitano”]. Holotype male in MZF.

Distribution. Palaearctic – Croatia, Italy (mainland, Sicily), Malta.

***partenopea*** (Rondani, 1861)

*Stevenia partenopea*Rondani 1861: 145. Type locality: Italy, Napoli. Holotype male in MZF.

*Stevenia florentina*Rondani 1861: 146. Type locality: Italy, Toscana [“Etruria”]. Holotype male in MZF.

*Chaetostevenia fischeri*Brauer 1895: 604. Type locality: Italy, Tivoli [“Tivoli bei Rom”]. Type(s), unspecified sex, in NHMW.

[Chaetostevenia parthenopaea]: Brauer (1895: 604); incorrect subsequent spelling of *C. partenopea* Rondani, 1861.

Distribution. Palaearctic – France (Corsica), Italy (mainland), Spain.

**Doubtful species in *Paykullia***

*Plesina liturata*Loew 1847: 260. Type locality: Italy, Trieste [“Die Gegend von Triest”]. Type(s), female, in ZMHB.

### Genus *Phyto* Robineau-Desvoidy, 1830

[Phyto] Robineau-Desvoidy MS name (Blainville et al. 1826: 22). Unavailable name; suppressed by action of ICZN. (1990: 162 [Opinion 1601]).

***Phyto***Robineau-Desvoidy 1830: 218. Type species: *Phyto nigra* Robineau-Desvoidy, 1830 [= *Tachina melanocephala* Meigen, 1824], by designation of Townsend (1916: 8).

Remarks: Both Crosskey (1977: 39; 1980: 820) and Herting (1993: 107) gave *Phyto nigra* Robineau-Desvoidy as type species of *Phyto*, considering Robineau-Desvoidy (1863: 47) to have proposed a valid subsequent designation of *Tachina melanocephala* Meigen by citing *P. nigra* in synonymy. However, Robineau-Desvoidy (1863: 47–48) cited not only *Phyto nigra* Robineau-Desvoidy, 1830 in synonymy with *T. melanocephala*, but also two other species originally included in *Phyto* by Robineau-Desvoidy (1830: 219): *P. prompta* Robineau-Desvoidy, 1830 and *P. palpalis* Robineau-Desvoidy, 1830. In accordance with Article 69.2.2. of the Code, a subsequent type fixation of “a nominal species that was not originally included” is accepted “if, but only if, at the same time he or she places that nominal species in synonymy with **one and only one** of the originally included species (as defined in Article 67.2) [...]” (our emphasis). This means that the current version of the Code rules out the type designation for *Phyto* by Robineau-Desvoidy (1863: 47–48) because he placed three of the nominal species originally included in *Phyto* in synonymy with *Tachina melanocephala*. The previous two versions of the Code (ICZN 1961, 1985) both had a slightly different and less precise wording for the same subarticle [i.e., 69(a)(iv)], which may have misled Crosskey (1980) and Herting (1993) to consider *Phyto nigra*, as one of the originally included species in *Phyto*, as the type species of *Phyto* by subsequent designation of Robineau-Desvoidy (1863).

*Cirillia*Rondani 1856: 80. Type species: *Cirillia angustifrons* Rondani, 1856, by original designation.

*Phito*Rondani 1861: 140. Unjustified emendation of *Phyto* Robineau-Desvoidy, 1830. [Name made available as an emendation by virtue of similar spelling changes in two or more names from “y” to “i” in the same work, *teste*O’Hara et al. (2011).]

*Savia*Rondani 1861: 140. Type species: *Tachina melanocephala* Meigen, 1824, by monotypy. Junior primary homonym of *Savia* Blumenbach, 1795 (Rodentia: Caviidae).

*Metopisena*Rondani 1862: 161. Type species: *Morinia celer* Rondani, 1862, by monotypy.

*Kockia*Robineau-Desvoidy 1863: 818. Type species: *Kockia claripennis* Robineau-Desvoidy, 1863 [= *Cirillia angustifrons* Rondani, 1856], by subsequent designation (Coquillett 1910: 557).

*Semitachina*Portschinsky 1883: 133. Type species: *Semitachina hylemyiaeformis* Portschinsky, 1883 [= *Tachina melanocephala* Meigen, 1824], by monotypy.

*Styloneuria*Brauer and Bergenstamm 1891: 365. Type species: *Styloneuria manni* Brauer & Bergenstamm, 1891 [= *Phyto adolescens* Rondani, 1861], by monotypy.

*Paramorinia*Brauer and Bergenstamm 1891: 367. Type species: *Paramorinia cincta* Brauer & Bergenstamm, 1891 [= *Tachina cingulata* Zetterstedt, 1844], by monotypy.

*Metopostena*Bezzi 1906: 53. Unjustified emendation of *Metopisena* Rondani, 1862.

*Cyrillia* Brauer 1898: 543. Unjustified emendation of *Cirillia* Rondani, 1856 [junior homonym of *Cyrillia*Robineau-Desvoidy 1863: 31 (placed in “Doubtful taxa in Tachinidae” by Herting and Dely-Draskovits (1993: 436))].

*Britea*Curran 1927: 127. Type species: *Britea tachinoides* Curran, 1927, by original designation.

*Diprodexia*Séguy 1935: 124. Type species: *Diprodexia lechevalieri* Séguy, 1935, by original designation.

*Protachaeta*Enderlein 1936: 225. Type species: *Phyto discrepans* Pandellé, 1896, by monotypy.

***abbreviata*** Villeneuve, 1920

*Phyto abbreviata*Villeneuve 1920: 202. Type locality: Algeria, Biskra. Holotype female not located.

Distribution. Palaearctic – Algeria, Israel, Tunisia [**new record**].

***adolescens*** Rondani, 1861

*Phyto adolescens*Rondani 1861: 139. Type locality: Italy, Parma. Lectotype male (by designation of Pape 1989: 355) in MZF.

*Styloneuria mannii*Brauer and Bergenstamm 1891: 365. Type locality: Croatia, Dubrovnik [“Ragusa”]. Type(s), female, in NHMW.

[Styloneuria manni]: Brauer and Bergenstamm (1891: 365); incorrect original spelling of *S. mannii* [*teste*Brauer and Bergenstamm (1891: errata p. 413)].

Distribution. Palaearctic – Croatia, Gibraltar, Greece [**new record**], Italy (mainland, Sicily), Hungary, Malta.

***anatolica*** Zeegers, 2011

*Phyto anatolica*Zeegers 2011: 314. Type locality: Turkey, Aksaray Province, Sultanhanı. Holotype male in SMNS.

Distribution. Palaearctic – Turkey.

***angustifrons*** (Rondani, 1856)

*Cirillia angustifrons*Rondani 1856: 80. Type locality: Italy, Parma. Lectotype male (by designation of Pape 1989: 356) in MZF.

*Kockia claripennis*Robineau-Desvoidy 1863: 819. Type locality: France, Nice [“dans la campagne de Nice”]. Type(s), male, not located.

[Cirillia ancustifrons]: Rondani (1856: 80); incorrect original spelling [*teste*Rondani (1856, unpaginated errata)].

Distribution. Palaearctic – France (mainland), Germany, Italy (mainland), Switzerland.

***armadillonis*** Kugler, 1978

*Phyto armadillonis*Kugler 1978: 87. Type locality: Israel, Mt Carmel. Holotype male in TAU.

Distribution. Palaearctic – Israel.

***atrior*** (Villeneuve, 1941)

*Styloneuria atrior*Villeneuve 1941: 122. Type locality: Morocco, Rabat. Holotype male in IRSNB.

Distribution. Palaearctic – Morocco.

***brevipila*** Herting, 1961

*Phyto brevipila*Herting 1961: 15. Type locality: Italy, Macugnaga. Holotype female in CNC.

Distribution. Palaearctic – Italy (mainland).

***celer*** (Rondani, 1862)

*Morinia celer*Rondani 1862: 161. Type locality: Italy, Parma. Lectotype male (by designation of Pape 1989: 356) in MZF.

Distribution. Palaearctic – Croatia, Italy (mainland), Hungary.

***cingulata*** (Zetterstedt, 1844)

*Tachina cingulata*Zetterstedt 1844: 1174. Type locality: Sweden, Östergötland: Gusum and Vadstena. Syntypes, male(s) and female(s), in MZLU.

*Paramorinia cincta*Brauer and Bergenstamm 1891: 368. Type locality: Italy, Trentino-Alto Adige, Merano [“Meran”]. Syntypes, male(s) and female(s), in NHMW.

*Styloneuria albidella*Villeneuve 1920: 201. Type locality: Austria, Tirol and France, Isère, St Pierre de Chartreuse (Dauphiné). Syntypes, 1 male and 1 female, not located.

Distribution. Palaearctic – Austria, Czech Republic (Bohemia), France (mainland), Italy (mainland), Norway, Sweden, Switzerland.

***discrepans*** Pandellé, 1896

*Phyto discrepans*Pandellé 1896: 132. Type locality: France, Hautes-Pyrénées, Tarbes. Syntypes, males, in MNHN.

[Phyto discreptans]: Verves (2005: 69); incorrect subsequent spelling of *discrepans* Pandellé, 1896.

Distribution. Nearctic [introduced] – Canada (Newfoundland). Palaearctic – Andorra, France (mainland), Gibraltar, Malta [**new record**], Morocco [**new record**], Poland, Portugal [**new record**], Spain, Tunisia [**new record**], Ukraine, United Kingdom (England).

***fernandezyepezi*** Báez, 1988

*Phyto fernandezyepezi*Báez 1988: 100. Type locality: Canary Islands, La Graciosa. Holotype male in TFMC.

Distribution. Palaearctic – Canary Islands (La Graciosa).

***hertingi*** Báez, 1979

*Phyto hertingi*Báez 1979a: 163. Type locality: Canary Islands, Gran Canaria, Puerto Rico. Holotype male in TFMC.

Distribution. Palaearctic – Canary Islands (Gran Canaria).

***latifrons*** Kugler, 1978

*Phyto latifrons*Kugler 1978: 89. Type locality: Israel, Zefat. Holotype male in TAU.

Distribution. Palaearctic – Israel.

***lechevalieri*** (Séguy, 1935)

*Diprodexia lechevalieri*Séguy 1935: 125. Type locality: Algeria, Rocher Blanc, le Corso. Holotype male in MNHN.

Phyto discrepans ssp. algeriensisHerting 1961: 17. Type locality: Algeria, Mascara. Holotype male in CNC.

Distribution. Palaearctic – Algeria.

***luteisquama*** Kugler, 1978

*Phyto luteisquama*Kugler 1978: 91. Type locality: Israel, Galilee, Ma’aloth. Holotype male in TAU.

Distribution. Palaearctic – Israel.

***melanocephala*** (Meigen, 1824)

*Tachina melanocephala*Meigen 1824: 281. Type locality: not given, probably Europe. Syntypes, females, 1 in MNHN.

*Tachina parvicornis*Meigen 1824: 282. Type locality: France, Paris, Bois de Boulogne. Syntypes, male(s) and female(s), in MNHN.

*Phyto nigra*Robineau-Desvoidy 1830: 219. Type locality: France, Paris. Type(s), unspecified sex, not located.

*Phyto palpalis*Robineau-Desvoidy 1830: 219. Type locality: France, Yonne, Saint-Sauveur-en-Puisaye. Type(s), unspecified sex, not located.

*Phyto prompta*Robineau-Desvoidy 1830: 219. Type locality: France, Paris, Bois de Boulogne. Type(s), female, not located.

*Clista maura*Perris 1852: 208. Type locality: France, Grandes-Landes. Type(s), unspecified sex, not located, possibly in ENSAM [*teste*Herting (1978: 2)].

*Tachina intercepta*Walker 1853: 34. Type locality: England. Syntypes, unspecified number and sex, lost [*teste*Crosskey (1974: 298)].

*Tachina nexa*Walker 1853: 63. Type locality: England. Lectotype male (by fixation of Crosskey 1974: 300) in NHMUK.

*Myobia micans*Macquart 1854: 442. Type locality: France, Landes, Mont-de-Marsan. Type(s), female, in MHNH.

*Semitachina hylemyiaeformis*Portschinsky 1883: 133. Type locality: western Transcaucasia [“Transcaucasus occident.”]. Type(s), male, not located.

Distribution. Palaearctic – Austria, Belgium, Croatia [**new record**], Czech Republic (Bohemia, Moravia), Denmark, France (mainland), Germany, Greece [**new record**], Hungary, Italy (mainland), Malta, Morocco, Netherlands, Poland, Russia (Central European Territory), Slovakia, Spain (mainland, Balearic Is), Sweden [**new record**], Switzerland, Turkey [**new record**], Ukraine (Cherkasy, Chernigiv, Chernivtzi, Crimea, Ivano-Frankivs’k, Kyiv, Kherson, Odesa, Poltava, Sumy, Zaporizzhya), United Kingdom (England).

***nigrobarbata*** (Becker, 1908)

*Styloneuria nigrobarbata*Becker 1908: 120. Type locality: Canary Islands, Tenerife, Guimar. Syntypes, 3 males in ZMHB.

Distribution. Palaearctic – Canary Islands (Tenerife).

***parafacialis*** Crosskey, 1977

*Phyto parafacialis*Crosskey 1977: 43. Type locality: South Africa, KwaZulu-Natal, Durban. Holotype male in NHMUK.

Distribution. Afrotropical – South Africa (KwaZulu-Natal).

***paratachinoides*** Crosskey, 1977

*Phyto paratachinoides*Crosskey 1977: 43. Type locality: Uganda, Bwamba valley. Holotype male in NHMUK.

Distribution. Afrotropical – Uganda.

***pauciseta*** Herting, 1961

*Phyto pauciseta*Herting 1961: 17. Type locality: Israel, Zikhon-Ya’akov. Holotype male in TAU.

Distribution. Palaearctic – Israel, Jordan.

***pilicornis*** (Villeneuve, 1920)

*Styloneuria pilicornis*Villeneuve 1920: 201. Type locality: Algeria, Tabarka and La Calle. Syntypes, males, 5 males in IRSNB.

Distribution. Palaearctic – Algeria.

***similis*** Stein, 1924

*Phyto similis*Stein 1924: 180. Type locality: Poland, Trzebiatow [“bei Treptow auf Rohr”]. Syntypes, 2 males and 7 females, in ZMHB.

Distribution. Palaearctic – France (mainland), Poland, Switzerland.

***sordidisquama*** Villeneuve, 1920

*Phyto sordidisquama*Villeneuve 1920: 202. Type locality: Tunesia, Tunis and Algeria: Alger and Mascara. Syntypes, male(s) and female(s), 1 male in IRSNB.

Distribution. Palaearctic – Algeria, Tunisia.

***subalbida*** Herting, 1961

*Phyto subalbida*Herting 1961: 19. Type locality: Spain, Noguera nr Albarracin. Holotype male in CNC.

Distribution. Palaearctic – Spain (mainland).

***tachinoides*** (Curran, 1927)

*Britea tachinoides*Curran 1927: 128. Type locality: Kenya, Lumbwa District, Kericho. Holotype male in NHMUK.

*Styloneuria maculosa*Villeneuve 1932: 272. Nomen nudum [first published in synonymy with *Britea tachinoides* Curran, 1927].

Distribution. Afrotropical – Kenya.

**Doubtful species in *Phyto***

*Phyto nigrogrisescens* [as *nigro-grisescens*] Robineau-Desvoidy 1830: 219. Type locality: France: Saint-Sauveur-en-Puisaye (Yonne) and Paris. Type(s), unspecified sex, not located.

*Triphera nigrifacies*Perris 1852: 207. Type locality: France, Grandes-Landes. Type(s), male, in ENSAM.

Remarks. Probably a senior synonym of *Phyto discrepans* (Pandellé, 1896), see Herting (1978: 2; 1993: 109).

### Genus *Queximyia* Crosskey, 1977

***Queximyia***Crosskey 1977: 45. Type species: *Queximyia flavipes* Crosskey, 1977, by original designation.

***flavipes*** Crosskey, 1977

*Queximyia flavipes*Crosskey 1977: 46. Type locality: South Africa, KwaZulu-Natal, Durban, Stella bush. Holotype male in NHMUK.

Distribution. Afrotropical – South Africa (Eastern Cape, Free State, KwaZulu-Natal).

### Genus *Rhinodonia* Cerretti, Lo Giudice & Pape, 2014

***Rhinodonia***Cerretti et al. 2014a: 662. Type species: *Rhinodonia antiqua* Cerretti, Lo Giudice & Pape, 2014, by original designation.

***antiqua*** Cerretti, Lo Giudice & Pape, 2014

*Rhinodonia antiqua*Cerretti et al. 2014a: 662. Type locality: New Caledonia, Mt Koghis, 800 m. Holotype male in CNC.

Distribution. Australasian – New Caledonia.

***flavicera*** Cerretti, Lo Giudice & Pape, 2014

*Rhinodonia flavicera*Cerretti et al. 2014a: 665. Type locality: New Caledonia, Pindal Forest, 3 km SW Nepoui. Holotype male in MNHN.

Distribution. Australasian – New Caledonia.

### Genus *Rhinomorinia* Brauer & Bergenstamm, 1889

***Rhinomorinia***Brauer and Bergenstamm 1889: 123. Type species: *Morinia sarcophagina* Schiner, 1862, by monotypy.

*Oxytachina*Brauer and Bergenstamm 1891: 369. Type species: *Oxytachina vittata* Brauer & Bergenstamm, 1891, by monotypy.

*Pseudophania*Brauer and Bergenstamm 1893: 139. Type species: *Pseudophania capensis* Brauer & Bergenstamm, 1893, by original designation.

*Dewetia*Bischof 1904: 95. Type species: *Dewetia atra* Bischof, 1904, by monotypy.

***approximata*** Crosskey, 1977

*Rhinomorinia approximata*Crosskey 1977: 29. Type locality: South Africa, Eastern Cape, Patensie District, Cambria area, Wit River valley (“3324DA”). Holotype male in NMDA.

Distribution. Afrotropical – South Africa (Eastern Cape).

***atra*** Bischof, 1904

*Dewetia atra*Bischof 1904: 97. Type locality: South Africa, Eastern Cape, Algoabaai [“Algoa-Bay”]. Holotype male in NHMW.

Distribution. Afrotropical – South Africa (Eastern Cape).

***bisetosa*** Crosskey, 1977

*Rhinomorinia bisetosa*Crosskey 1977: 35. Type locality: South Africa, Western Cape, Bredasdorp District, Arniston coastal dunes. Holotype male in NMDA.

Distribution. Afrotropical – South Africa (Western Cape).

***capensis*** (Brauer & Bergenstamm, 1893)

*Pseudophania capensis*Brauer and Bergenstamm 1893: 139. Type locality: South Africa, Western Cape, Cape of Good Hope. Lectotype male (by fixation of Villeneuve 1918: 504) in NHMW.

Distribution. Afrotropical – South Africa (Western Cape).

***longifacies*** Herting, 1966

*Rhinomorinia longifacies*Herting 1966: 451. Type locality: Nepal, Taplejung District, between Sangu and Tamrang, 1700 m. Holotype male in NHMUK.

Distribution. Oriental – Nepal.

***sarcophagina*** (Schiner, 1862)

*Morinia sarcophagina* Schiner 1862: 552. Type locality: Italy, Trieste and Austria, Wien [“Um Triest und auch bei Wien”]. Syntypes, males, in NHMW.

*Rhinomorinia minor*Strobl 1894: 33 (as var. of *sarcophagina* Schiner). Type locality: Austria, Sunk nr Hohentauern and Austria, Kalbling nr Admont. Syntypes, 1 male and 1 female (Sunk), 1 female (Kalbling), in NMBA.

*Rhinomorinia subrostrata*Villeneuve 1913: 178. Type locality: Italy, Macugnaga. Syntypes, male(s) and female(s), not located.

Distribution. Palaearctic – Austria, Belgium, Croatia, Czech Republic (Bohemia, Moravia), Germany, Hungary, Italy (mainland), Poland, Slovakia, Switzerland, Ukraine (Cherkasy, Chernivtzy, Ivano-Frankivs’k, Kyiv, Lugansk, Poltava, Sumy).

***scutellata*** Crosskey, 1977

*Rhinomorinia scutellata*Crosskey 1977: 34. Type locality: South Africa, Western Cape, Stellenbosch. Holotype female in NHMUK.

Distribution. Afrotropical – South Africa (Western Cape).

***setitibia*** Crosskey, 1977

*Rhinomorinia setitibia*Crosskey 1977: 32. Type locality: South Africa, KwaZulu-Natal, Hluhluwe. Holotype male in NHMUK.

Distribution. Afrotropical – Mozambique, South Africa (KwaZulu-Natal).

***verticalis*** Crosskey, 1977

*Rhinomorinia verticalis*Crosskey 1977: 33. Type locality: South Africa, Western Cape, George District, Outeniqua Pass. Holotype male in NMDA.

Distribution. Afrotropical – South Africa (Western Cape).

***vittata*** (Brauer & Bergenstamm, 1891)

*Oxytachina vittata*Brauer and Bergenstamm 1891: 396. Type locality: South Africa, Western Cape, Cape of Good Hope. Holotype female in NHMW.

Distribution. Afrotropical – South Africa (Western Cape).

***xanthocephala*** (Bezzi, 1908)

*Hoplisa xanthocephala*Bezzi 1908: 187. Type locality: Namibia, Rooibank, nr Walvis Bay. Lectotype male (by designation of Crosskey 1977: 36) in ZMHB.

*Hoplisa novicia*Villeneuve 1916: 511. Type locality: South Africa, Transvaal, Barberton. Lectotype male (by designation of Crosskey 1977: 36) in SAMC.

Distribution. Afrotropical – Namibia, South Africa (Eastern Cape, Northern Cape, Western Cape, KwaZulu-Natal, Free State, Transvaal).

### Genus *Rhinopeza* Cerretti, Lo Giudice & Pape, 2014

***Rhinopeza***Cerretti et al. 2014a: 666. Type species: *Rhinopeza gracilis* Cerretti, Lo Giudice & Pape, 2014, by original designation.

***gracilis*** Cerretti, Lo Giudice & Pape, 2014

*Rhinopeza gracilis*Cerretti et al. 2014a: 666. Type locality: New Guinea, NE Mt Hagen, 5°48'S, 143°57'E. Holotype male in BPBM.

Distribution. Australasian – Papua New Guinea.

### Genus *Rhinophora* Robineau-Desvoidy, 1830

*Rhinophora*Robineau-Desvoidy 1830: 258. Type species: *Rhinophora gagatea* Robineau-Desvoidy, 1830 [= *Tachina lepida* Meigen, 1824], by subsequent designation of Townsend (1916:8) see Evenhuis et al. (2010: 144).

[Rhynophoba]: Neave (1940: 68); incorrect subsequent spelling of *Rhinophora* Robineau-Desvoidy, 1830.

***lepida*** (Meigen, 1824)

*Musca parcus*Harris 1780: 144. Type locality: England. Type(s), unspecified sex, not located. Nomen oblitum [*teste* this work]. Stat. rev.

*Tachina lepida*Meigen 1824: 289. Type locality: not given, Europe. Holotype male in MNHN. Nomen protectum [*teste* this work].

Remarks. The nominal taxon *Musca parcus* was treated as a nomen dubium in Rhinophoridae by Herting (1993: 117), and as a synonym or tentative synonym of *Rhinophora lepida* by Thompson and Pont (1994) and Chandler (1998), respectively. We are here accepting the synonymy, but in accordance with ICZN Article 23.9, we consider the junior synonym *Rhinophora lepida* to be the valid name for this taxon. We have not found any work after 1899 treating the name *Musca parcus* as valid, for which reason it qualifies as a nomen oblitum, and the name *Rhinophora lepida* fulfils the requirements for being a nomen protectum by having been used as the presumed valid name in at least the following 25 works published since 1967: Bedding (1973), Crosskey (1977), Kugler (1978), Tschorsnig (1985), Pape (1986), Herting (1993), Draber-Mońko (1997), Chandler (1998), Pape (1998a), Dunk (1999), Szpila (1999), Wijnhoven and Zeegers (1999), Pape and Arnaud (2001), Wijnhoven (2001), Carles-Tolrá et al. (2003), Lutovinovas (2004), Verves (2005), Peris and González-Mora (2007), Rudzinski and Flügel (2007), Schacht et al. (2007), Kutty et al. (2010), Rognes (2010), Verves and Khrokalo (2010), Ebejer (2011), Emer et al. (2015).

*Tachina nana*Stephens 1829a: 65; 1929b: 299. Nomen nudum [*teste*Crosskey (1974: 299)].

Remarks: *Tachina nana* of Stephens was later described by Walker (1853: 39) based on Stephens’ material. The single male specimen in Stephens’ collection was considered the holotype of *Tachina nana* Walker, 1853 [= *Rhinophora lepida* (Meigen, 1824)] by Crosskey (1974: 299). Herting (1993) overlooked Crosskey’s paper on Walker’s types and treated *Tachina nana* Stephens as a nomen dubium.

*Rhinophora gagatea*Robineau-Desvoidy 1830: 259. Type locality: France. Type(s), unspecified sex, not located.

*Rhinophora metallica*Robineau-Desvoidy 1830: 259. Type locality: France: Paris and Saint-Sauveur-en-Puisaye (Yonne). Type(s), unspecified sex, not located.

*Rhinophora tessellata*Robineau-Desvoidy 1830: 259. Type locality: France: Paris and Saint-Sauveur-en-Puisaye (Yonne). Type(s), unspecified sex, not located.

*Rhinophora nigripennis*Robineau-Desvoidy 1830: 259. Type locality: Not given, France. Type(s), unspecified sex, not located. [Tentative synonym].

*Rhinophora hottentota*Robineau-Desvoidy 1830: 260. Type locality: Not given, France. Type(s), unspecified sex, not located. [Tentative synonym].

*Rhinophora pusilla*Robineau-Desvoidy 1830: 260. Type locality: France, Saint-Sauveur. Type(s), unspecified sex, not located. [Tentative synonym].

*Leucostoma aenescens*Zetterstedt 1844: 1234. Type locality: Sweden, Skåne: Räften nr Lund and Esperöd. Syntypes, male(s) and female(s), in MZLU.

*Tachina nana*Walker 1853: 39. Type locality: England. Lectotype male (by fixation of Crosskey 1974: 300) in NHMUK.

Remarks: Walker (1853) indicated neither the sex nor the number of specimens of the material he studied. Crosskey’s (1974: 299) mention that “Stephen’s [sic] collection contained one male specimen standing under this name [*Tachina nana*]. This specimen [...] is the holotype of *T. nana* Walker” would serve as a lectotype fixation by inference of holotype.

*Clista heteropalpis*Macquart 1855a: 42. Type locality: Switzerland, Graubünden, Malans nr Landquart. Type(s), male, not located (possibly in MNHM).

*Clista ignota*Brauer and Bergenstamm 1889: 136. Type locality: Central Europe [“M.-Europa”]. Type(s), male, in NHMW.

Distribution. Palaearctic – Belgium, Bulgaria, Cyprus, Czech Republic (Bohemia, Moravia), Denmark, France (mainland), Germany, Hungary, Ireland, Italy (mainland [**new record**]), Netherlands, Poland, Russia (NW European part [Leningrad Reg.], Voronyezh, North Caucasus [Caucasus Reserve, Dagestan]), Slovakia, Spain (mainland), Sweden, Switzerland, Ukraine (Cherkasy, Chernigiv, Ivano-Frankivs’k, Kherson, Kyiv, Lugansk, Mykolayiv, Vinnytzya, Zaporizzhya), United Kingdom (England).

### Genus *Shannoniella* Townsend, 1939

***Shannoniella***Townsend 1939: 249. Type species: *Shannoniella cuspidata* Townsend, 1939, by original designation.

***cuspidata*** Townsend, 1939

*Shannoniella cuspidata*Townsend 1939: 251. Type locality: Brazil, Rio de Janeiro. Holotype male in USNM (unique specimen identifier: USNMENT01519742).

Distribution. Neotropical – Brazil (Paraná, Rio de Janeiro, São Paulo).

***setinervis*** Nihei, Andrade, Pape & Cerretti, 2016

*Shannoniella setinervis*Nihei et al. 2016: 89. Type locality: Brazil, Rio de Janeiro, Teresópolis. Holotype male in MZSP.

Distribution. Neotropical – Brazil (Paraná, Rio de Janeiro).

### Genus *Stevenia* Robineau-Desvoidy, 1830

***Stevenia***Robineau-Desvoidy 1830: 220. Type species: *Stevenia tomentosa* Robineau-Desvoidy, 1830 [= *Tachina atramentaria* Meigen, 1824], by subsequent designation of Desmarest in d’Orbigny (1848: 32).

[Sterenia]: Lioy (1864: 68); incorrect subsequent spelling of *Stevenia* Robineau-Desvoidy, 1830

*Trisonevra*Lioy 1864: 68. Type species: *Ptilocera cilipennis* Macquart, 1835 [= *Tachina atramentaria* Meigen, 1824], by monotypy.

*Eophyto*Townsend 1919b: 163. Type species: *Eophyto ceylanica* Townsend, 1919, by original designation.

*Ptiloceroides*Villeneuve 1924: 31. Type species: *Ptilocera lateralis* Macquart, 1849, by monotypy.

*Astevenia*Belanovsky 1951: 122 (as subgenus of *Stevenia* Robineau-Desvoidy, 1830). Type species: Stevenia (Astevenia) nudiseta Belanovsky, 1951, by monotypy.

[Ptilochaeta]: Brauer and Bergenstamm (1889: 121), Bezzi and Stein (1907: 455); incorrect subsequent spelling of *Ptilocheta* Rondani, 1857.

***actenata*** Zeegers, 2008

*Stevenia actenata*Zeegers 2008: 737. Type locality: Yemen, Lahj. Holotype male in ZMA.

Distribution. Afrotropical – Yemen.

***acutangula*** (Villeneuve, 1910)

*Rhinophora acutangula*Villeneuve 1910: 86. Type locality: France, Dauphiné: Grenoble and Col du Lautaret. Syntypes, male(s) and female(s), not located.

Distribution. Palaearctic – France (mainland), Switzerland.

***angustifrons*** Villeneuve, 1912

*Stevenia angustifrons*Villeneuve 1912: 50. Type locality: Syria, Oasis de Damas. Holotype male in IRSNB.

*Stevenia inops*Villeneuve 1934b: 54. Type locality: Israel, Rehoboth nr Jaffa. Syntypes, 1 male and 1 female, not located.

Distribution. Palaearctic – Iran, Israel, Russia (North Caucasus [Teberda]), Syria, Turkey, Ukraine (Zakarpattya).

***atramentaria*** (Meigen, 1824)

*Tachina atramentaria*Meigen 1824: 291. Type locality: [Germany] and Austria. Syntypes, male(s) and female(s), in MNHN.

*Dexia melania*Meigen 1826: 40. Type locality: unknown [“Waterland unbekannt”]. Type(s), female, in MNHN.

*Musca putris*Stephens 1829b: 302. Unavailable name first published in synonymy with “*Dexia melania*” [*teste*Crosskey (1974: 298)].

*Stevenia tomentosa*Robineau-Desvoidy 1830: 220. Type locality: France, Gentilly nr Paris and Anjou. Syntypes, male(s) and female(s), not located.

*Stevenia velox*Robineau-Desvoidy 1830: 221. Type locality: France, Anjou. Type(s), unspecified sex, not located.

*Ptilocera rectangularis*Macquart 1834: 235. Type locality: France, Lille. Type(s), female, not located.

*Ptilocera cilipennis*Macquart 1835: 172. Type locality: northern France [“Du nord de la France”]. Type(s), male, not located.

*Rhinophora lucidiventris*Loew 1847: 269. Type locality: Turkey, Ephesus. Syntypes, males, not located, possibly in ZMHB.

*Rhinophora inornata*Loew 1847: 271. Type locality: Austria, Vienna [“Die Gegend von Wien”]. Syntypes, females, not located, possibly in ZMHB.

*Tachina caminaria*Walker 1853: 35. Type locality: England. Lectotype female (by fixation of Crosskey 1974: 298) in NHMUK.

*Rhinophora fuscipennis*Macquart 1855b: 186. Type locality: France, Landes, Mont-de-Marsan. Type(s), female, not located.

*Ptilocheta bertolinii*Rondani 1862: 140. Type locality: Italy, Parma. Tridentino. Holotype male in MZF.

*Ptilocheta galeazzii*Rondani 1862: 141. Type locality: Italy, Lombardia [“Insubria alpina”]. Holotype male in MZF.

[Ptilocheta bertolonii]: Strobl (1894: 52); incorrect subsequent spelling of *Ptilocheta bertolinii* Rondani, 1862.

[Ptilocheta galeazzi]: Rondani (1862: 237); incorrect original spelling of *P. galeazzii* [*teste*Pape (1989: 356)].

Distribution. Palaearctic – Austria, Belgium, Bulgaria, Czech Republic (Bohemia, Moravia), Finland, France (mainland), Germany, Greece [**new record**], Hungary, Italy (mainland, Sicily), Jordan, Norway, Poland, Romania, Russia (NW European part [Leningrad Reg.], North Caucasus [environs of Kislovodsk, Caucasus Reserve]), Slovakia, Sweden, Switzerland, Turkey, Ukraine (Cherkasy, Chernigiv, Dnipropetrovs’k, Kyiv, Kirovograd, Odesa), United Kingdom (Wales, England).

***bertei*** (Rondani, 1865), stat. rev.

*Ptilocheta bertei*Rondani 1865: 227. Type locality: Italy, Apennines near Parma. Holotype male in MZF.

Remarks. *Ptilocheta bertei* was listed as a nomen dubium by Herting (1993), but it is here resurrected as a valid species based on our examination of the holotype male, which was recently rediscovered in the Rondani collection at MZF. The specimen is pinned and in good condition. Following the key to Palaearctic *Stevenia* provided by Cerretti and Pape (2007), *Stevenia bertei* runs to couplet 18 and is separable from *S. etrusca* by male with three proclinate orbital setae (anterior one distinctly longer), one upper reclinate orbital seta, abdomen yellow laterally on tergites 1+2 and 3, and abdominal tergites 1+2, 3 and 4 without median discal setae.

Distribution. Palaearctic – Croatia [**new record**], Italy (mainland).

***ceylanica*** (Townsend, 1919)

*Eophyto ceylanica*Townsend 1919b: 164. Type locality: Sri Lanka, Peradeniya. Holotype female in USNM (unique specimen identifier: USNMENT01519741).

Distribution. Oriental – Sri Lanka.

***deceptoria*** (Loew, 1847)

*Rhinophora deceptoria*Loew 1847: 266. Type locality: Italy, Sicily, Syracuse [“Sicilien, bei Syrakus” (= Siracusa)]. Syntypes, male(s) and female(s), in ZMHB.

*Rhinophora deceptricula*Loew 1847: 267. Type locality: Italy, Sicily, Syracuse [“Sicilien, bei Syrakus” (= Siracusa)]. Syntypes, male(s) and female(s), in ZMHB.

*Rhinophora subpellucida*Loew 1847: 265. Type locality: Italy: Sicily, Mt Etna and Umbria, Spoleto [“Sicilien, ...., auf dem Aetna” and “Ein Männchen ... bei Spoleto gefangen”]. Type(s), male, not located (possibly lost).

*Ptilocheta passerinii*Rondani 1862: 138. Type locality: Italy: Apennines nr Parma and Toscana [“Etruria”]. Lectotype male (by designation of Pape 1989: 357) in MZF.

Distribution. Nearctic [introduced] – USA (Ohio). Neotropical [introduced] – Argentina (Buenos Aires). Palaearctic – Andorra, Croatia, France (mainland, Corsica), Gibraltar, Italy (mainland, ? Sardinia, Sicily), Malta, Morocco, Portugal, Spain (mainland, Balearic Is), Switzerland, United Kingdom (England).

***eggeri*** (Strobl, 1906)

Rhinophora (Ptilocheta) eggeriStrobl 1906: 341. Type locality: Croatia [“Dalmatien”]. Syntypes, 5 unspecified sex, in NHMW.

*Stevenia steini*Villeneuve 1931: 65. Type locality: Croatia, Split [“Spalato”]. Holotype male not located.

Distribution. Palaearctic – Croatia, Italy (mainland).

***etrusca*** Cerretti & Pape, 2007

*Stevenia etrusca*Cerretti and Pape 2007: 35. Type locality: Italy, Tuscany, Grosseto province, Scarlino, Cala di Terra Rossa. Holotype male in MZUR.

Distribution. Palaearctic – Italy (mainland).

***fausti*** (Portschinsky, 1875)

*Rhinophora fausti*Portschinsky 1875: 27. Type locality: Russia, Dagestan [“Caucasus, Daghestan”]. Type(s), female, not located.

Distribution. Palaearctic – Greece, Russia (North Caucasus [Dagestan], S European territory), Ukraine (Donetz’k, Zaporizzhya).

***fernandezi*** Báez, 1979

*Stevenia fernandezi*Báez 1979b: 23. Type locality: Canary Islands, Tenerife, Las Mercedes. Holotype male in TFMC.

Distribution. Palaearctic – Canary Islands (Tenerife).

***flaviventris*** Kugler, 1978

*Stevenia flaviventris*Kugler 1978: 96. Type locality: Israel, Jordan Valley, Bet Shean. Holotype male in TAU.

Distribution. Palaearctic – Cyprus, Israel.

***gilasiani*** Ziegler, Gisondi & Cerretti, 2019

*Stevenia gilasiani* Ziegler, Gisondi & Cerretti in Gisondi et al. 2019: 425. Type locality: Iran, West Azerbaijan, Ulugh Dagh mountain range south of Urmia. Holotype male in ZMHB.

***hertingi*** Kugler, 1978

*Stevenia hertingi*Kugler 1978: 98. Type locality: Israel, Yizrael. Holotype male in TAU.

Distribution. Palaearctic – Israel.

***hirtigena*** Herting, 1961

*Stevenia hirtigena*Herting 1961: 25. Type locality: Iran, Khuzistan, Jarrahi river bank. Holotype male in SMNS.

Distribution. Palaearctic – Iran, Israel [**new record**], Oman.

***kugleri*** Herting, 1961

Stevenia triangulata ssp. kugleriHerting 1961: 27. Type locality: Israel, Zichron Ya’akov. Holotype male in TAU.

Distribution. Palaearctic – Israel, Turkey.

***lateralis*** (Macquart, 1849)

*Ptilocera lateralis*Macquart 1849: 481. Type locality: Algeria: La Calle and Constantine. Syntypes, males, not located.

Distribution. Palaearctic – Algeria, Tunisia.

***maeotica*** Belanovsky, 1951

*Stevenia maeotica*Belanovsky 1951: 122. Type locality: Ukraine, Donetz’k Region, near Tshistjakovo. Lectotype male (by designation of Verves 2005: 72) in UASK.

Distribution. Palaearctic – Russia (North Caucasus [Krasnodar Kray, Arkhipo-Osipovsk]), Ukraine (Donetz’k, Zaporizzhya).

***nudiseta*** Belanovsky, 1951

Stevenia (Astevenia) nudisetaBelanovsky 1951: 122, 126. Type locality: Ukraine, Donetz’k Region, Zhdanov [= Mariupol’]. Holotype female not located (possibly lost).

Remarks. There are two original spellings of this species-group name in Belanovsky (1951): *nudiseta* (pages 122 and 126) and *incerta* (page 121). Acting as First Reviser, we select *nudiseta* as the correct original spelling.

[Stevenia (Astevenia) incerta]: Belanovsky (1951: 121); incorrect original spelling of S. (A.) nudiseta [*teste* this work].

Distribution. Palaearctic – Ukraine (Donetz’k, Lugansk, Odesa, Zaporizzhya).

***obscuripennis*** (Loew, 1847)

*Rhinophora obscuripennis*Loew 1847: 264. Type locality: Italy, Terni Province, Narni [“bei Rom und nördlich von Rom”]. Lectotype male (by fixation of Herting 1961: 26) in SMNS.

*Ptilocheta tacchetti*Rondani 1865: 227. Type locality: Italy, Bologna [= Bononiae]. Holotype male in MZF. Syn. nov.

Remarks. In the Rondani collection there are two males and one female under *P. tacchetti*. One male is pinned and in good condition, the other one is glued sideways on a rectangular card and is moldy. The male specimens are conspecific. Given that Rondani in the original description clearly refers to a single male specimen that appears to be in good condition, we consider the pinned male to be the holotype of *P. tacchetti*.

[Ptilocheta tachettii]: Herting (1993: 114); incorrect subsequent spelling of *P. tacchetti* Rondani, 1865 [*teste* this work].

Distribution. Palaearctic – Croatia, Italy (mainland), Malta.

***palermitana*** Cerretti & Pape, 2007

*Stevenia palermitana*Cerretti and Pape 2007: 32. Type locality: Italy, Sicily, Palermo Province, Bosco della Ficuzza. Holotype male in MZUR.

Distribution. Palaearctic – Italy (Sicily).

***pannonica*** Villeneuve, 1919

*Stevenia pannonica*Villeneuve 1919: 265. Type locality: Romania, Herkulesbad [= Băile Herculane] and Orsova and Mehadia. Syntypes, males, in IRSNB.

Distribution. Palaearctic – Romania.

***sardoa*** Villeneuve, 1920, stat. rev.

*Stevenia sardoa*Villeneuve 1920: 200. Type locality: Italy, Sardinia. Syntypes, males and females, 2 males and 1 female in IRSNB.

Remarks. *Stevenia sardoa* Villeneuve, 1920 was treated as a synonym of *Rhinophora deceptoria* Loew, 1847 by Herting (1961: 24, 1993: 112), but it is here recognized as a valid species based on examination of the three syntypes in IRSNB. Using the key of Cerretti and Pape (2007), specimens of *Stevenia sardoa* run to *S. deceptoria* (couplet 19), with which it shares narrow basal bands of weak microtomentum confined to the anterior 1/4–1/3 of abdominal tergites 3 and 4. However, *S. sardoa* differs from *S. deceptoria* in having the fore and hind femora at least partly red.

Distribution. Palaearctic – Italy (Sardinia).

***signata*** (Mik, 1866)

*Rhinophora signata*Mik 1866: 307. Type locality: Italy, Mt Czavn nr Gorizia [“Berges Czavn bei Görz”]. Holotype male not located (possibly in NHMW).

Distribution. Palaearctic – Albania, Croatia, Greece, Italy (mainland), Russia (S European territory [environs of Astrakhan]), Turkey [**new record**].

***socotrensis*** Crosskey, 1977

*Stevenia socotrensis*Crosskey 1977: 49. Type locality: Socotra, Adho, Diemellus, 1100 m. Holotype male in NHMUK.

Distribution. Afrotropical – Socotra.

***subalbida*** (Villeneuve, 1911), stat. rev.

*Rhinophora subalbida*Villeneuve 1911: 121. Type locality: France, Corsica, Bonifacio. Syntypes, males and females, 5 males in IRSNB.

Remarks. *Rhinophora subalbida* Villeneuve, 1911 was treated as a junior synonym of *Rhinophora deceptoria* Loew, 1847 by Herting (1961: 24, 1993: 112), but it is here recognised as a valid species based on examination of the five syntypes in IRSNB. Specimens of *Stevenia subalbida* run to *S. deceptoria* in the key of Cerretti and Pape (2007) (couplet 19) but they differ from *S. deceptoria* in having a thicker cover of greyish reflecting microtomentum on the scutum, abdominal syntergite 1+2 dorsally almost entirely covered with greyish microtomentum and abdominal tergites 3 and 4 with wide bands of microtomentum on basal 2/5–1/2.

Distribution. Palaearctic – France (Corsica).

***triangulata*** (Loew, 1847)

*Rhinophora triangulata*Loew 1847: 263. Type locality: Greece, Rhodes. Syntypes, male(s) and female(s), not located (possibly in ZMHB).

Distribution. Palaearctic – Greece.

***umbratica*** (Fallén, 1820)

*Ocyptera umbratica*Fallén 1820: 7. Type locality: Sweden: Skåne and Öland. Syntypes, males and 1 female, in NHRS.

*Rhinophora bicincta*Meigen 1838: 210. Type locality: Spain, Andalusia. Type(s), female, not located.

*Rhinophora hyalinata*Zetterstedt 1844: 1231. Type locality: Sweden, Lund. Holotype male in MZLU.

*Rhinophora lugubris*Zetterstedt 1855: 4706. Type locality: Sweden, Skåne, Gualöv. Type(s), female, in MZLU.

*Rhinophora simplicissima*Loew 1847: 270. Type locality: Poland, Poznan [“Die Posener Gegend”]. Type(s), female, not located (possibly in ZMHB).

*Phyto rondanii*Pandellé 1896: 131. Type locality: Italy: Apennines nr Parma and Sicily. Type(s), male, in MZF.

*Rhinophora distans*Czerny and Strobl 1909: 225. Nomen nudum.

Distribution. Palaearctic – Austria, Belgium, Czech Republic (Bohemia), France (mainland), Germany, Hungary, Italy (mainland), Norway, Poland, Portugal, Slovakia, Spain (mainland), Sweden, Switzerland, Tunisia [**new record**], Ukraine (Cherkasy, Kyiv, Odesa).

**Doubtful species in *Stevenia***

*Rhinophora caucasica*Portschinsky 1875: 28. Type locality: Caucasus. Type(s), female, not located.

*Rhinophora distinguenda*Mik 1866: 308. Type locality: Italy, between Trieste and Miramare [“an der Strasse zwischen Triest und Miramare”]. Holotype male not located (possibly in ZMHB).

*Rhinophora laeviventris*Loew 1847: 268. Type locality: Greece, Rhodes. Type(s), male, not located (possibly in ZMHB).

*Rhinophora obliqua*Macquart 1855b: 188. Type locality: Europe [Not given.]. Type(s), male, not located.

*Rhinophora pallidicornis*Loew 1847: 270. Type locality: Turkey, Makri [“Die Gegend von Makri in Kleinasien”]. Type(s), female, not located (possibly in ZMHB).

*Rhinophora perpendicularis*Macquart 1855b: 188. Type locality: France, Pas-de-Calais, Lestrem. Syntypes, male(s) and female(s), not located.

### Genus *Tricogena* Rondani, 1856

***Tricogena***Rondani 1856: 88. Type species: *Tricogena truquii* Rondani, 1856 [= *Tachina rubricosa* Meigen, 1824], by original designation.

[Tricogenia]: Rondani (1856: 225); incorrect original spelling of *Tricogena* Rondani, 1856 [*teste*Neave (1950: 276)].

*Talmonia*Robineau-Desvoidy 1863: 704. Type species: *Talmonia tibialis* Robineau-Desvoidy, 1863 [= *Tachina rubricosa* Meigen, 1824], by original designation.

*Frauenfeldia*Egger 1865: 297. Type species: *Tachina rubricosa* Meigen, 1824, by monotypy.

[Trichogena]: Brauer and Bergenstamm (1889: 124); incorrect subsequent spelling of *Tricogena* Rondani, 1856.

[Thricogena]: Neave (1940: 470); incorrect subsequent spelling of *Tricogena* Rondani, 1856.

Remarks: Neave incorrectly considered *Thricogena* a spelling of *Tricogena*, whereas it is an unjustified emendation of *Thrychogena* Rondani, 1856 [senior but invalid synonym of *Loewia* Egger, 1856 (Tachinidae), *teste*O’Hara et al. (2011)].

***rubricosa*** (Meigen, 1824)

*Tachina rubricosa*Meigen 1824: 305. Type locality: not given, probably Germany. Type(s), male, in MNHN.

*Tachina trilineata*Meigen 1824: 281. Type locality: not given, probably Germany. Type(s), female, in MNHN.

*Tachina barbata* Meigen 1830: 371. Type locality: Germany, Stolberg. Type(s), female, in MNHN.

*Tachina genibarbis* Meigen 1830: 372. Type locality: Germany, Berlin. Type(s), male, not located (possibly lost).

*Tachina hirticornis*Zetterstedt 1844: 1172. Type locality: Sweden: Öland and Gotland and Östergötland, Gusum and Bohuslän, Marstrand. Syntypes, males, in MZLU.

*Tachina nigritarsis*Zetterstedt 1844: 1169. Type locality: Sweden: Skåne, Esperöd and Skåne, Mellby and Östergötland, Vadstena and Öland. Syntypes, females, in MZLU.

*Tachina tarsalis*Zetterstedt 1844: 1170. Nomen nudum (ascribed to Boheman).

*Dexia tachiniformis* Zetterstedt, 1844: 1280. Type locality: Sweden, Öland. Holotype male in MZLU.

*Tricogena truquii*Rondani 1856: 88. Type locality: Italy, Piemonte alps [“in Pedemontii alpibus”]. Holotype male in MZF.

*Talmonia tibialis*Robineau-Desvoidy 1863: 705. Type locality: France, near Paris. Holotype male not located (possibly lost).

*Frauenfeldia monticola*Brauer and Bergenstamm 1891: 413. Nomen nudum [as “*monticola* Schum. litt.”].

[Tricogena lumbricosa]: Verves (2012: 31); incorrect subsequent spelling of *Tricogena rubricosa* (Meigen, 1824).

Distribution. Palaearctic – Belgium, Czech Republic (Bohemia, Moravia), Denmark, Finland, France (mainland), Germany, Ireland, Italy (mainland), Morocco, Norway, Poland, Portugal, Russia (NW European part [Leningrad Region]), Slovakia, Spain (mainland), Sweden, Switzerland, Tunisia, Ukraine (Kyiv, Zhytomyr), United Kingdom (England).

### Genus *Tromodesia* Rondani, 1856

***Tromodesia***Rondani 1856: 87. Type species: *Tromodesia vibripennis* Rondani, 1856, by original designation.

*Mimodexia*Rohdendorf 1935: 96. Type species: *Mimodexia magnifica* Rohdendorf, 1935, by original designation. Syn. nov.

*Callidesia*Kugler 1978: 75. Type species: *Callidesia pictipennis* Kugler, 1978, by original designation.

***angustifrons*** Kugler, 1978

*Tromodesia angustifrons*Kugler 1978: 102. Type locality: Israel, Carmel, Yoqneam. Holotype male in TAU.

Distribution. Palaearctic – Greece, Israel, Turkey.

***guzari*** (Rohdendorf, 1935), comb. nov.

*Mimodexia guzari*Rohdendorf 1935: 98. Type locality: Uzbekistan, Bukhara, Guzar. Holotype female in ZIN.

Distribution. Palaearctic – Uzbekistan.

***intermedia*** (Rohdendorf, 1935), comb. nov.

*Mimodexia intermedia*Rohdendorf 1935: 98. Type locality: Turkmenia, Ashkabad and Uzbekistan, Bukhara, Chargush. Syntypes, 2 males, possibly in ZIN.

Distribution. Palaearctic – Turkmenia, Uzbekistan.

***lindneriana*** (Rohdendorf, 1961), comb. nov.

*Mimodexia lindneriana*Rohdendorf 1961: 10. Type locality: Iran, Baluchistan, Makran, Gozomir 50 km NW Geh. Syntypes, 2 males, in SMNS.

Distribution. Palaearctic – Iran.

***magnifica*** (Rohdendorf, 1935), comb. nov.

*Mimodexia magnifica*Rohdendorf 1935: 99. Type locality: Uzbekistan, Bukhara, Bag-Absal 50 km NE Staraja Buchara. Syntypes, 10 males, possibly in ZIN and ZMUM.

Distribution. Palaearctic – Uzbekistan.

***obscurior*** (Rohdendorf, 1935), comb. nov.

*Mimodexia obscurior*Rohdendorf 1935: 100. Type locality: Turkmenia, Kara-Kala. Syntypes, 1 male and 1 female, possibly in ZIN.

Distribution. Palaearctic – Turkmenia.

***pallidissima*** (Rohdendorf, 1935), comb. nov.

*Mimodexia pallidissima*Rohdendorf 1935: 101. Type locality: Uzbekistan, Bukhara, Yargak nr Khatyrchi. Syntypes, 1 male and 2 females, possibly in ZIN.

Distribution. Palaearctic – Uzbekistan.

***pictipennis*** (Kugler, 1978)

*Callidesia pictipennis*Kugler 1978: 76. Type locality: Israel, Mt Hermon. Holotype male in TAU.

Distribution. Palaearctic – Israel, Turkey.

***setiventris*** (Rohdendorf, 1935), comb. nov.

*Mimodexia setiventris*Rohdendorf 1935: 101. Type locality: Turkmenia, Tashauz. Holotype male, possibly in ZIN.

Distribution. Palaearctic – Pakistan [**new record**], Turkmenia.

***shachrudi*** (Rohdendorf, 1935), comb. nov.

*Mimodexia shachrudi*Rohdendorf 1935: 102. Type locality: Iran, Shahrud. Holotype female, possibly in ZIN.

Distribution. Palaearctic – Armenia, Iran.

***vibripennis*** Rondani, 1856

*Tromodesia vibripennis*Rondani 1856: 87. Type locality: Italy, Parma. Lectotype female (by designation of Pape 1989: 357) in MZF.

Morinia (Melanomya) trifasciataStrobl 1901: 223. Austria, Kärnten, Federaun. Holotype male in NMBA.

*Morinia tricingulata*Strobl 1902: 490. New name for *Morinia trifasciata* Strobl, 1901.

[vitripennis] Strobl (1902: 490); incorrect subsequent spelling of *vibripennis* Rondani, 1856.

Distribution. Palaearctic – Austria, Italy (mainland).

### Genus *Trypetidomima* Townsend, 1935

***Trypetidomima***Townsend 1935: 68. Type species *Trypetidomima lutea* Townsend, 1935, by original designation.

***fusca*** Nihei & Andrade, 2014

*Trypetidomima fusca*Nihei and Andrade 2014: 729. Type locality: Brazil, São Paulo, Santo André, Paranapiacaba District. Holotype male in MZSP.

Distribution. Neotropical – Brazil (São Paulo).

***lutea*** Townsend, 1935

*Trypetidomima lutea*Townsend 1935: 68. Type locality: Brazil, São Paulo, Itaquaquecetuba. Holotype male in USNM (unique specimen identifier: USNMENT01519738).

Distribution. Neotropical – Brazil (Paraná, Rio de Janeiro, São Paulo).

### Genus *Ventrops* Crosskey, 1977

***Ventrops***Crosskey 1977: 20. Type species: *Ventrops milichioides* Crosskey, 1977, by original designation.

***aethiopicus*** Cerretti & Pape, 2012

*Ventrops aethiopicus*Cerretti and Pape 2012: 276. Type locality: Ethiopia, Shewa, Akaki, 20 km SE Addis Abeba, 8°54'N, 38°47'E [as 38°47''E]. Holotype male in TAU.

Distribution. Afrotropical – Ethiopia.

***freidbergi*** Cerretti & Pape, 2012

*Ventrops freidbergi*Cerretti and Pape 2012: 280. Type locality: Tanzania, Usambara Mts, Gologolo. Holotype male in TAU.

Distribution. Afrotropical – Tanzania.

***hannemariae*** Pape, 1987

*Ventrops hannemariae*Pape 1987b: 544. Type locality: South Africa, KwaZulu-Natal, 20 mi N Jozini. Holotype male in NMDA.

Distribution. Afrotropical – South Africa (KwaZulu-Natal).

***incisus*** Pape, 1987

*Ventrops incisus*Pape 1987b: 547. Type locality: Tanzania, East Usambara, Amani, 1000 m. Holotype male in NHMD.

Distribution. Afrotropical – Tanzania.

***intermedius*** Pape, 1987

*Ventrops intermedius*Pape 1987b: 546. Type locality: Tanzania, Udzungwa, Mwanihana forest above Sanje. Holotype male in NHMD.

Distribution. Afrotropical – Tanzania.

***milichioides*** Crosskey, 1977

*Ventrops milichioides*Crosskey 1977: 20. Type locality: Zimbabwe, N Vumba. Holotype male in NMDA.

Distribution. Afrotropical – Kenya, Tanzania, South Africa, Zimbabwe.

***stuckenbergi*** Cerretti & Pape, 2012

*Ventrops stuckenbergi*Cerretti and Pape 2012: 283. Type locality: Namibia, Rundu District, 18°18'22"S, 19°15'24"E. Holotype male in NMNW.

Distribution. Afrotropical – Namibia, South Africa.

***vikhrevi*** Cerretti, Ziegler & Pape, 2015

*Ventrops vikhrevi*Cerretti et al. 2015: 580. Type locality: Ethiopia, Amhara, Zengena L., 2530 m, 10.910N 36.965E. Holotype male in ZMUM.

Distribution. Afrotropical – Ethiopia.

### Nominal taxa unplaced within Rhinophoridae

**Genera**

*Cassidaemyia*Macquart 1835:162. Type species: *Tachina gagatina* Meigen, 1824, by designation of Westwood, 1840: 139. Nomen dubium in Rhinophoridae [*teste*Herting (1993: 117)].

*Cassidemya*Rondani 1861: 85. Unjustified emendation of *Cassidaemyia* Macquart, 1835 [*teste* O’Hara et al. (2001: 47)].

**Species**

*Tachina expetita*Walker 1853: 36. Type locality: England. Type(s), unspecified sex, lost [*teste*Crosskey (1974: 298)].

*Tachina interlatens*Walker 1853: 35. Type locality: England. Type(s), unspecified sex, lost [*teste*Crosskey (1974: 299)].

*Tachina gagatina*Meigen 1824: 287. Type locality: France, Vaucluse, Carpentras. Syntypes, male(s) and female(s), not located [*teste* this work].

*Melanophora pygmaea* Macquart 1855: 203. Type locality: France, Landes, Mont-de-Marsan. Type(s), male, not located. [Possibly Tachinidae, *teste*Herting (1993: 117)].

*Melanophora tombae*Gistel 1857: 40 [as “*Melanophorae*”]. “Algarbia”. Syntypes, females, not located. [Possibly not Rhinophoridae, *teste* this work].

*Melanophora urnae*Gistel 1857: 40. “Algarbia”. Syntypes, females, not located. [Possibly not Rhinophoridae, *teste* this work].

### Review of changes since Crosskey (1977) and Herting (1993)

**Family re-assignments – exclusions**

*Alvamaja*Rognes 2010: 4. Valid genus in the family Polleniidae [*teste*Cerretti et al. (2019)].

*Angioneura*Brauer and Bergenstamm 1893: 187. Valid genus in the family Calliphoridae [*teste*Pape (1986)], subfamily Melanomyinae [*teste*Rognes (1991, 1997, 2011)].

*Leucostoma nervosa*Stephens 1829a: 59; 1829b: 300. Nomen nudum in the subfamily Melanomyinae [= *Melanomya nana* (Meigen, 1826) (Calliphoridae), *teste*Crosskey (1974: 298)].

*Melanomya*Rondani 1856: 88. Valid genus in the family Calliphoridae [*teste*Pape (1986) and Rognes (1986), see also Downes (1986)], subfamily Melanomyinae [*teste*Rognes (1991, 1997, 2011)].

*Morinia*Robineau-Desvoidy 1830: 264. Valid genus in the family Calliphoridae [*teste*Pape (1986)], subfamily Polleniinae [*teste*Rognes (1991, 1997, 2011)]; family Polleniidae [*teste*Cerretti et al. (2019)].

*Phyto carinata* Pape 1987: 378. Transferred to the genus *Morinia* Robineau-Desvoidy in the family Polleniidae [*teste*Cerretti et al. (2019)].

*Phyto lactineala*Pape 1997: 160. Transferred to the genus *Morinia* Robineau-Desvoidy in the family Polleniidae [*teste*Cerretti et al. (2019)].

*Phyto longirostris*Crosskey 1977: 44. Transferred to the genus *Morinia* Robineau-Desvoidy in the family Polleniidae [*teste*Cerretti et al. (2019)].

*Phyto royi*Pape 1997: 163. Transferred to the genus *Morinia* Robineau-Desvoidy in the family Polleniidae [*teste*Cerretti et al. (2019)].

*Phyto stuckenbergi*Crosskey 1977: 44. Transferred to the genus *Morinia* Robineau-Desvoidy in the family Polleniidae [*teste*Cerretti et al. (2019)].

*Termitoloemus*Baranov 1936: 647. Valid genus in the family Calliphoridae [*teste*Pape (1986)], subfamily Bengaliinae [*teste*Rognes (2011) and Singh and Rognes (2015)].

**Family re-assignments – inclusions**

*Aporeomyia*Pape and Shima 1993: 77. Transferred to Rhinophoridae from Tachinidae [*teste* this work].

*Axinia* Colless 1994: 484. Transferred to Rhinophoridae from Axiniidae [*teste*Pape (1998a)].

*Bezzimyia*Townsend 1919a: 591. Transferred to Rhinophoridae from Tachinidae [*teste*Pape and Arnaud (2001)].

*Malayia*Malloch 1926: 510. Transferred to Rhinophoridae from Tachinidae [*teste*Pape and Shima (1993)].

*Shannoniella*Townsend 1939: 249. Transferred to Rhinophoridae from Tachinidae [*teste*Pape (1998a)].

*Trypetidomima*Townsend 1935: 68. Transferred to Rhinophoridae from Tachinidae [*teste*Pape (1998a)].

**Changes within the Rhinophoridae (except new taxa and new combinations)**

*Bequaertiana*Curran 1929: 14. Junior synonym of *Melanophora* Meigen [*teste*Cerretti and Pape (2009)]. Treated as a valid genus by Crosskey (1977).

*Barrinea*Colless 1994a: 511. Junior synonym of *Axinia* Colless [*teste*Peris and González-Mora (2007)].

*Chirops*Colless 1994a: 516. Junior synonym of *Axinia* Colless [*teste*Peris and González-Mora (2007)].

*Cirillia*Rondani 1856: 80. Junior synonym of *Phyto* Robineau-Desvoidy [*teste*Pape (1986: 23)]. Treated as a valid genus by Herting (1961: 19, 1993: 110).

*Frauenfeldia caucasica*Villeneuve 1908: 287. Valid specie of *Acompomintho* Villeneuve [*teste* this work]. Treated as unplaced to genus by Herting (1961: 34, 1993: 117).

*Frauenfeldia sinensis*Villeneuve 1936: 7. Valid specie of *Acompomintho* Villeneuve [*teste* this work]. Treated a nomen dubium by Herting (1961: 34, 1993: 117).

*Hoplisa caesia*Villeneuve 1911. Valid species of *Oplisa* Rondani [*teste* Pape (2004), see Pape et al. (2015)]. Treated as a nomen dubium by Herting (1993: 117).

*Mimodexia*Rohdendorf 1935: 96. Junior synonym of *Tromodesia* Rondani [*teste* this work]. Treated as a valid genus by Herting (1993: 106).

*Ptilocheta bertei*Rondani 1865: 227. Valid species of *Stevenia* Robineau-Desvoidy [*teste* this work]. Treated as a nomen dubium by Herting (1993: 114).

*Ptilocheta tacchetti*Rondani 1865: 227. Junior synonym of *Stevenia obscuripennis* (Loew) [*teste* this work]. Treated as a nomen dubium by Herting (1993: 114, as *P. tacchettii*).

*Stevenia sardoa* Villeneuve, 1920: 200. Valid species of *Stevenia* Robineau-Desvoidy [*teste* this work]. Treated as a junior synonym of *Stevenia deceptoria* (Loew, 1847) by Herting (1961: 24, 1993: 112).

*Rhinophora subalbida*Villeneuve 1911: 121. Valid species of *Stevenia* Robineau-Desvoidy [*teste* this work]. Treated as a junior synonym of *Stevenia deceptoria* (Loew, 1847) by Herting (1961: 24, 1993: 112).

*Tachina nana* Stephens 1829. Nomen nudum [= *Rhinophora lepida* (Meigen, 1824), *teste*Crosskey (1974: 299)].

*Ptilocheta bertolinii*Rondani 1862: 140. Junior synonym of *Stevenia atramentaria* (Meigen, 1824) [*teste*Pape (1989: 356)]. Treated as a junior synonym of *Stevenia deceptoria* (Loew, 1847) by Herting (1961: 24); treated as a nomen dubium in *Stevenia* by Herting (1993: 114).

*Stevenia florentina*Rondani 1861: 146. Junior synonym of *Paykullia partenopea* (Rondani, 1861) [*teste*Pape (1989: 356)]. Treated as a nomen dubium in *Paykullia* by Herting (1993: 116).

## Supplementary Material

XML Treatment for Maurhinophora

XML Treatment for Maurhinophora indoceanica

XML Treatment for Marshallicona

XML Treatment for Marshallicona quitu

XML Treatment for Neotarsina

XML Treatment for Neotarsina andina

XML Treatment for Neotarsina caraibica

XML Treatment for Kinabalumyia

XML Treatment for Kinabalumyia pinax

XML Treatment for Kinabalumyia

XML Treatment for Kinabalumyia

## Footnotes

1 If both thorax and abdomen are black, and abdomen is without median discal setae, run also from couplet 41

2 in part, except P. pauciseta species group

3R. sarcophagina (Schiner), in part

4 (in part, M. argyriventris species group)

5 in part, M. roralis species group

6 (in part, V. milichioides species group)

7 (in part, V. hannemariae and V. aethiopicus species groups)
